# Supplementary material for: Multimorbidity patterns in low-middle and high income regions: a multiregion latent class analysis using ATHLOS harmonised cohorts
Source: BMJ Open. 2020 Jul 19;10(7):e034441. doi: 10.1136/bmjopen-2019-034441 (PMC7371222; doi:10.1136/bmjopen-2019-034441)
Supplement: Supplementary data [file bmjopen-2019-034441supp001.pdf]

Table S1. Proportion of missingness in the variables of interest in Mexico

(a) Indicators of multimorbidity classes	
	n (%) missingness
Diabetes	2814 (51.65)
Hypertension	2815 (51.67)
Joint disorders	2814 (51.65)
Asthma	2814 (51.65)
Chronic lung disease	2814 (51.65)
Myocardial infarction – Angina	2814 (51.65)
Stroke	2814 (51.65)
Depression	2815 (51.67)
(b) Variables used in the regression model	
Sex	2706 (49.67)
Age	2707 (49.69)
Country	0 (0.00)
Study	0 (0.00)
Marital status	2811 (51.60)
Education	3264 (59.91)
Wealth	1922 (35.28)
Loneliness	2819 (51.74)
Ever smoked	2814 (51.65)
Vigorous exercise in last 2 weeks	2814 (51.65)
Self-rated health	2811 (51.6)
ADL – Using the toilet	2814 (51.65)
ADL – Bathing or showering	2815 (51.67)
ADL – Getting dressed	0 (0.00)
ADL - Eating	2814 (51.65)
ADL – Getting in or out of bed	2813 (51.63)
ADL – Moving around the house	2817 (51.71)
Memory: Immediate recall	2854 (52.39)
Memory: Delayed recall	2854 (52.39)
Verbal fluency	2852 (52.35)

Table S2. Overall proportion of missingness for indicators of latent classes and variables included in the imputation model

(a) Indicators of multimorbidity classes	
	n (%) missingness
Diabetes	62 (0.08)
Hypertension	154 (0.21)
Joint disorders	31 (0.04)
Asthma	82 (0.11)
Chronic lung disease	51 (0.07)
Myocardial infarction – Angina	63 (0.08)
Stroke	41 (0.05)
Depression	1233 (1.70)
(b) Variables used in the regression model	
Sex	0 (0.00)
Age	0 (0.00)
Country	0 (0.00)
Study	0 (0.00)
Marital status	106 (0.14)
Education	11475 (15.91)
Wealth	844 (1.17)
Loneliness	10985 (15.23)
Ever smoked	201 (0.27)
Vigorous exercise in last 2 weeks	258 (0.35)
Self-rated health	218 (0.30)
ADL – Using the toilet	355 (0.49)
ADL – Bathing or showering	340 (0.47)
ADL – Getting dressed	304 (0.42)
ADL - Eating	400 (0.55)
ADL – Getting in or out of bed	310 (0.42)
ADL – Moving around the house	345 (0.47)
Memory: Immediate recall	1442 (1.99)
Memory: Delayed recall	1448 (2.00)
Verbal fluency	1641 (2.27)

Table S3. Proportion of missingness for indicators of latent classes and variables included in the imputation model for region 1 (SAGE study - Africa: Ghana, South Africa)

(a) Indicators of multimorbidity classes	
	n (%) missingness
Diabetes	1 (0.01)
Hypertension	1 (0.01)
Joint disorders	3 (0.03)
Asthma	1 (0.01)
Chronic lung disease	1 (0.01)
Myocardial infarction – Angina	3 (0.03)
Stroke	1 (0.01)
Depression	17 (0.21)
(b) Variables used in the regression model	
Sex	0 (0.00)
Age	0 (0.00)
Country	0 (0.00)
Study	0 (0.00)
Marital status	89 (1.11)
Education	3705 (46.6)
Wealth	23 (0.28)
Loneliness	48 (0.60)
Ever smoked	7 (0.08)
Vigorous exercise in last 2 weeks	12 (0.15)
Self-rated health	15 (0.18)
ADL – Using the toilet	40 (0.50)
ADL – Bathing or showering	32 (0.40)
ADL – Getting dressed	27 (0.33)
ADL - Eating	31 (0.38)
ADL – Getting in or out of bed	32 (0.40)
ADL – Moving around the house	35 (0.44)
Memory: Immediate recall	36 (0.45)
Memory: Delayed recall	31 (0.38)
Verbal fluency	9 (0.11)

Table S4. Proportion of missingness for indicators of latent classes and variables included in the imputation model for region 2 (SAGE study - Eastern Asia: China)

(a) Indicators of multimorbidity classes	
	n (%) missingness
Diabetes	35 (0.27)
Hypertension	127 (0.98)
Joint disorders	2 (0.01)
Asthma	53 (0.41)
Chronic lung disease	23 (0.17)
Myocardial infarction – Angina	34 (0.26)
Stroke	13 (0.10)
Depression	146 (1.13)
(b) Variables used in the regression model	
Sex	0 (0.00)
Age	0 (0.00)
Country	0 (0.00)
Study	0 (0.00)
Marital status	8 (0.06)
Education	3105 (24.18)
Wealth	18 (0.14)
Loneliness	74 (0.57)
Ever smoked	2 (0.01)
Vigorous exercise in last 2 weeks	45 (0.35)
Self-rated health	16 (0.12)
ADL – Using the toilet	51 (0.39)
ADL – Bathing or showering	52 (0.40)
ADL – Getting dressed	41 (0.31)
ADL - Eating	46 (0.35)
ADL – Getting in or out of bed	36 (0.28)
ADL – Moving around the house	48 (0.37)
Memory: Immediate recall	317 (2.46)
Memory: Delayed recall	399 (3.10)
Verbal fluency	335 (2.60)

Table S5. Proportion of missingness for indicators of latent classes and variables included in the imputation model for region 2 (SAGE study - Southern Asia: India)

(a) Indicators of multimorbidity classes	
	n (%) missingness
Diabetes	0 (0.00)
Hypertension	1 (0.01)
Joint disorders	1 (0.01)
Asthma	0 (0.00)
Chronic lung disease	0 (0.00)
Myocardial infarction – Angina	0 (0.00)
Stroke	1 (0.01)
Depression	1 (0.01)
(b) Variables used in the regression model	
Sex	0 (0.00)
Age	0 (0.00)
Country	0 (0.00)
Study	0 (0.00)
Marital status	0 (0.00)
Education	0 (0.00)
Wealth	38 (0.57)
Loneliness	10 (0.15)
Ever smoked	1 (0.01)
Vigorous exercise in last 2 weeks	0 (0.00)
Self-rated health	0 (0.00)
ADL – Using the toilet	19 (0.28)
ADL – Bathing or showering	12 (0.18)
ADL – Getting dressed	11 (0.16)
ADL - Eating	73 (1.11)
ADL – Getting in or out of bed	7 (0.10)
ADL – Moving around the house	24 (0.36)
Memory: Immediate recall	88 (1.34)
Memory: Delayed recall	88 (1.34)
Verbal fluency	71 (1.08)

Table S6. Proportion of missingness for indicators of latent classes and variables included in the imputation model for region 2 (SAGE study - Eastern Europe: the Russian Federation)

(a) Indicators of multimorbidity classes	
	n (%) missingness
Diabetes	5 (0.12)
Hypertension	4 (0.10)
Joint disorders	3 (0.07)
Asthma	6 (0.15)
Chronic lung disease	5 (0.12)
Myocardial infarction – Angina	5 (0.12)
Stroke	5 (0.12)
Depression	14 (0.36)
(b) Variables used in the regression model	
Sex	0 (0.00)
Age	0 (0.00)
Country	0 (0.00)
Study	0 (0.00)
Marital status	6 (0.15)
Education	41 (1.05)
Wealth	5 (0.12)
Loneliness	79 (2.03)
Ever smoked	1 (0.02)
Vigorous exercise in last 2 weeks	0 (0.00)
Self-rated health	5 (0.12)
ADL – Using the toilet	47 (1.20)
ADL – Bathing or showering	46 (1.18)
ADL – Getting dressed	27 (0.69)
ADL - Eating	52 (1.33)
ADL – Getting in or out of bed	37 (0.95)
ADL – Moving around the house	40 (1.02)
Memory: Immediate recall	153 (3.93)
Memory: Delayed recall	81 (2.08)
Verbal fluency	208 (5.35)

Table S7. Proportion of missingness for indicators of latent classes and variables included in the imputation model for region 2 (ELSA study- England)

(a) Indicators of multimorbidity classes	
	n (%) missingness
Diabetes	2 (0.01)
Hypertension	2 (0.01)
Joint disorders	3 (0.02)
Asthma	3 (0.02)
Chronic lung disease	3 (0.02)
Myocardial infarction – Angina	2 (0.01)
Stroke	2 (0.01)
Depression	372 (3.23)
(b) Variables used in the regression model	
Sex	0 (0.00)
Age	0 (0.00)
Country	0 (0.00)
Study	0 (0.00)
Marital status	0 (0.00)
Education	1023 (8.88)
Wealth	330 (2.86)
Loneliness	356 (3.09)
Ever smoked	173 (1.50)
Vigorous exercise in last 2 weeks	176 (1.52)
Self-rated health	175 (1.51)
ADL – Using the toilet	173 (1.50)
ADL – Bathing or showering	173 (1.50)
ADL – Getting dressed	173 (1.50)
ADL - Eating	173 (1.50)
ADL – Getting in or out of bed	173 (1.50)
ADL – Moving around the house	173 (1.50)
Memory: Immediate recall	360 (3.12)
Memory: Delayed recall	373 (3.23)
Verbal fluency	362 (3.14)

Table S8. Proportion of missingness for indicators of latent classes and variables included in the imputation model for region 2 (SHARE study - Northern Europe: Denmark, Sweden)

(a) Indicators of multimorbidity classes	
	n (%) missingness
Diabetes	2 (0.04)
Hypertension	2 (0.04)
Joint disorders	2 (0.04)
Asthma	2 (0.04)
Chronic lung disease	2 (0.04)
Myocardial infarction – Angina	2 (0.04)
Stroke	2 (0.04)
Depression	78 (1.70)
(b) Variables used in the regression model	
Sex	0 (0.00)
Age	0 (0.00)
Country	0 (0.00)
Study	0 (0.00)
Marital status	0 (0.00)
Education	52 (1.13)
Wealth	6 (0.13)
Loneliness	1384 (30.26)
Ever smoked	4 (0.08)
Vigorous exercise in last 2 weeks	6 (0.13)
Self-rated health	2 (0.04)
ADL – Using the toilet	3 (0.06)
ADL – Bathing or showering	3 (0.06)
ADL – Getting dressed	3 (0.06)
ADL - Eating	3 (0.06)
ADL – Getting in or out of bed	3 (0.06)
ADL – Moving around the house	3 (0.06)
Memory: Immediate recall	68 (1.48)
Memory: Delayed recall	66 (1.44)
Verbal fluency	83 (1.81)

Table S9. Proportion of missingness for indicators of latent classes and variables included in the imputation model for region 2 (SHARE study - Southern Europe: Greece, Italy, Spain)

(a) Indicators of multimorbidity classes	
	n (%) missingness
Diabetes	12 (0.16)
Hypertension	12 (0.16)
Joint disorders	12 (0.16)
Asthma	12 (0.16)
Chronic lung disease	12 (0.16)
Myocardial infarction – Angina	12 (0.16)
Stroke	12 (0.16)
Depression	194 (2.59)
(b) Variables used in the regression model	
Sex	0 (0.00)
Age	0 (0.00)
Country	0 (0.00)
Study	0 (0.00)
Marital status	1 (0.01)
Education	20 (0.26)
Wealth	95 (1.27)
Loneliness	2715 (36.37)
Ever smoked	3 (0.04)
Vigorous exercise in last 2 weeks	4 (0.05)
Self-rated health	0 (0.00)
ADL – Using the toilet	3 (0.04)
ADL – Bathing or showering	3 (0.04)
ADL – Getting dressed	3 (0.04)
ADL - Eating	3 (0.04)
ADL – Getting in or out of bed	3 (0.04)
ADL – Moving around the house	3 (0.04)
Memory: Immediate recall	125 (1.67)
Memory: Delayed recall	125 (1.67)
Verbal fluency	176 (2.35)

Table S10. Proportion of missingness for indicators of latent classes and variables included in the imputation model for region 2 (SHARE study - Western Europe: Austria, Belgium, France, Germany, Israel, Netherlands, Switzerland)

(a) Indicators of multimorbidity classes	
	n (%) missingness
Diabetes	5 (0.02)
Hypertension	5 (0.02)
Joint disorders	5 (0.02)
Asthma	5 (0.02)
Chronic lung disease	5 (0.02)
Myocardial infarction – Angina	5 (0.02)
Stroke	5 (0.02)
Depression	411 (2.36)
(b) Variables used in the regression model	
Sex	0 (0.00)
Age	0 (0.00)
Country	0 (0.00)
Study	0 (0.00)
Marital status	2 (0.01)
Education	165 (0.95)
Wealth	329 (1.89)
Loneliness	6319 (36.42)
Ever smoked	10 (0.05)
Vigorous exercise in last 2 weeks	15 (0.08)
Self-rated health	5 (0.02)
ADL – Using the toilet	19 (0.10)
ADL – Bathing or showering	19 (0.10)
ADL – Getting dressed	19 (0.10)
ADL - Eating	19 (0.10)
ADL – Getting in or out of bed	19 (0.10)
ADL – Moving around the house	19 (0.10)
Memory: Immediate recall	295 (1.7)
Memory: Delayed recall	285 (1.64)
Verbal fluency	397 (2.28)
