# Supplementary material for: Multimorbidity patterns in low-middle and high income regions: a multiregion latent class analysis using ATHLOS harmonised cohorts
Source: BMJ Open. 2020 Jul 19;10(7):e034441. doi: 10.1136/bmjopen-2019-034441 (PMC7371222; doi:10.1136/bmjopen-2019-034441)
Supplement: Supplementary data [file bmjopen-2019-034441supp002.pdf]

Table S1. Association between latent multimorbidity membership and outcomes in individuals aged 50-64 and ≥65

Outcomes[a]	Aged 50-64		Aged ≥65	
	"Cardio-metabolic" class	"Respiratory-mental-articular" class	"Cardio-metabolic" class	"Respiratory-mental-articular" class
Sex				
Woman	1.0	1.0	1.0	1.0
Male	0.9 (0.8-1.0)	0.6 (0.5-0.7)	0.7 (0.7-0.8)	0.9 (0.8-1.0)
Age (years)	1.1 (1.1-1.1)	1.1 (1.0-1.1)	1.0 (1.0-1.0)	1.0 (1.0-1.0)
Marital status				
Single	1.0	1.0	1.0	1.0
Married	0.9 (0.8-1.1)	0.7 (0.5-1.0)	1.2 (1.00-1.4)	1.2 (0.9-1.6)
Divorced	1.1 (0.9-1.4)	1.7 (1.2-2.4)	1.1 (1.0-1.4)	1.8 (1.2-2.6)
Widowed	1.4 (1.1-1.7)	1.1 (0.8-1.6)	1.5 (1.3-1.8)	1.6 (1.2-2.3)
Education level				
Primary or less	1.0	1.0	1.0	1.0
Secondary	0.9 (0.9-1.0)	0.7 (0.6-0.8)	1.0 (0.9-1.0)	0.7 (0.6-0.8)
Tertiary	0.8 (0.7-0.9)	0.6 (0.5-0.7)	0.9 (0.8-1.0)	0.6 (0.5-0.7)
Wealth				
1st (worse)	1.0	1.0	1.0	1.0
2nd	1.0 (0.9-1.1)	0.8 (0.6-1.0)	1.1 (1.0-1.2)	1.1 (0.9-1.2)
3rd	0.9 (0.9-1.1)	0.7 (0.5-0.8)	1.1 (1.0-1.2)	1.0 (0.9-1.2)
4th	0.9 (0.8-1.0)	0.6 (0.5-0.8)	1.1 (1.0-1.2)	0.8 (0.7-1.0)
5th (best)	0.8 (0.7-0.9)	0.5 (0.4-0.6)	1.2 (1.0-1.2)	0.7 (0.6-0.9)
Region				
Africa	1.0	1.0	1.0	1.0
China	0.9 (0.8-1.0)	0.3 (0.2-0.5)	1.8 (1.6-2.1)	4.1 (3.0-5.7)
India	0.9 (0.8-1.1)	2.2 (1.5-3.2)	0.9 (0.8-1.0)	2.9 (2.0-4.2)
Russia	3.6 (3.0-4.2)	2.2 (1.4-3.5)	8.0 (7.0-9.2)	14.5 (10.3-20.3)
England	1.1 (0.9-1.3)	5.6 (4.1-7.7)	2.2 (2.0-2.5)	6.2 (4.5-8.5)
Northern Europe	1.3 (1.1-1.5)	2.8 (1.9-4.1)	1.9 (1.6-2.2)	4.2 (2.9-6.1)
Southern Europe	1.5 (1.3-1.8)	1.7 (1.2-2.5)	2.8 (2.5-3.2)	6.5 (4.7-9.0)
Western Europe	1.5 (1.3-1.7)	1.9 (1.3-2.6)	2.1 (1.9-2.3)	3.6 (2.6-5.0)
Loneliness (yes/no)	1.9 (1.8-2.1)	2.5 (2.1-3.0)	1.4 (1.4-1.6)	1.9 (1.7-2.2)
Ever smoked (yes/no)	1.0 (0.9-1.1)	1.7 (1.5-2.0)	0.8 (0.8-0.8)	1.4 (1.3-1.6)
Physical activity (yes/no)	0.5 (0.5-0.5)	0.6 (0.5-0.7)	0.5 (0.5-0.6)	0.5 (0.4-0.6)
Limitations in ADL (yes/no)	2.5 (2.3-2.7)	3.2 (2.7-3.7)	1.8 (1.7-1.9)	2.8 (2.5-3.1)
Self-rated health				
Good	1.0	1.0	1.0	1.0
Moderate	3.7 (3.3-3.9)	2.5 (2.1-2.9)	2.5 (2.3-2.7)	3.9 (3.3-4.6)
Poor	8.5 (7.7-9.4)	7.4 (6.1-8.8)	4.3 (4.6)	10.9 (9.3-12.8)
Memory: Immediate recall	0.9 (0.9-0.9)	0.9 (0.9-1.0)	0.9 (0.9-0.9)	0.9 (0.9-0.9)
Memory: Delayed recall	0.9 (0.9-0.9)	0.9 (0.8-0.9)	0.9 (0.9-0.9)	0.9 (0.9-0.9)
Verbal fluency	1.0 (1.0-1.0)	1.0 (1.0-1.0)	1.0 (0.9-1.00)	1.0 (0.9-1.0)

Note: The reference group for the multimorbidity group variable was the "Healthy" class
Relative Risk Ratios (95% confidence interval) from multinomial logistic regression models
Models were run in 100 imputed datasets and results combined using Rubin's rules
ADL Activities of Daily Living
[a]Unadjusted analyses.
