# Supplementary material for: Multimorbidity patterns in low-middle and high income regions: a multiregion latent class analysis using ATHLOS harmonised cohorts
Source: BMJ Open. 2020 Jul 19;10(7):e034441. doi: 10.1136/bmjopen-2019-034441 (PMC7371222; doi:10.1136/bmjopen-2019-034441)
Supplement: Author's manuscript [file bmjopen-2019-034441.draft_revisions.pdf]

BMJ Open

BMJ Open is committed to open peer review. As part of this commitment we make the peer review history of every article we publish publicly available.

When an article is published we post the peer reviewers' comments and the authors' responses online. We also post the versions of the paper that were used during peer review. These are the versions that the peer review comments apply to.

The versions of the paper that follow are the versions that were submitted during the peer review process. They are not the versions of record or the final published versions. They should not be cited or distributed as the published version of this manuscript.

BMJ Open is an open access journal and the full, final, typeset and author-corrected version of record of the manuscript is available on our site with no access controls, subscription charges or pay-per-view fees (http://bmjopen.bmj.com).

If you have any questions on BMJ Open's open peer review process please email info.bmjopen@bmj.com

BMJ Open

Multimorbidity patterns in low-middle and high income regions: A multi-region latent class analysis using ATHLOS harmonized cohorts.

Journal:	*BMJ Open*
Manuscript ID	bmjopen-2019-034441
Article Type:	Original research
Date Submitted by the Author:	19-Sep-2019
Complete List of Authors:	Bayes-Marin, Ivet; Parc Sanitari Sant Joan de Déu, Research, Innovation and Teaching Unit; Centro de Investigación Biomédica en Red de Salud Mental, CIBERSAM<br>Sanchez-Niubo, Albert ; Parc Sanitari Sant Joan de Déu, Research, Innovation and Teaching Unit; Centro de Investigación Biomédica en Red de Salud Mental, CIBERSAM<br>Egea-Cortés, Laia; Parc Sanitari Sant Joan de Déu, Research, Innovation and Teaching Unit<br>Nguyen, Hai; King's College London, Health Service and Population Research Department<br>Prina, A.Matthew; King's College London,<br>Haro, Josep; Parc Sanitari Sant Joan de Deu, CIBERSAM, University of Barcelona; King Sand University<br>Olaya, Beatriz; Parc Sanitari Sant Joan de Deu, Research and Development Unit
Keywords:	multimorbidity, non-communicable diseases (NCDs), low- and middle-income countries (LMICs), high-income country (HICs), Latent class analysis (LCA)

SCHOLARONE™
Manuscripts

I, the Submitting Author has the right to grant and does grant on behalf of all authors of the Work (as defined in the below author licence), an exclusive licence and/or a non-exclusive licence for contributions from authors who are: i) UK Crown employees; ii) where BMJ has agreed a CC-BY licence shall apply, and/or iii) in accordance with the terms applicable for US Federal Government officers or employees acting as part of their official duties; on a worldwide, perpetual, irrevocable, royalty-free basis to BMJ Publishing Group Ltd ("BMJ") its licensees and where the relevant Journal is co-owned by BMJ to the co-owners of the Journal, to publish the Work in this journal and any other BMJ products and to exploit all rights, as set out in our licence.

The Submitting Author accepts and understands that any supply made under these terms is made by BMJ to the Submitting Author unless you are acting as an employee on behalf of your employer or a postgraduate student of an affiliated institution which is paying any applicable article publishing charge ("APC") for Open Access articles. Where the Submitting Author wishes to make the Work available on an Open Access basis (and intends to pay the relevant APC), the terms of reuse of such Open Access shall be governed by a Creative Commons licence – details of these licences and which Creative Commons licence will apply to this Work are set out in our licence referred to above.

Other than as permitted in any relevant BMJ Author's Self Archiving Policies, I confirm this Work has not been accepted for publication elsewhere, is not being considered for publication elsewhere and does not duplicate material already published. I confirm all authors consent to publication of this Work and authorise the granting of this licence.

For peer review only

Original Research Article

Manuscript title:

Multimorbidity patterns in low-middle and high income regions: A multi-region latent class analysis using ATHLOS harmonized cohorts.

Author's full names and affiliations:

Ivet Bayes-Marin, [a,b] Albert Sanchez-Niubo, [a,b] Laia Egea-Cortés, [a] Hai Nguyen, [c] Matthew Prina, [c] Josep Maria Haro, [a,b] and Beatriz Olaya [a,b]

From the [a]Research, Innovation and Teaching Unit, Parc Sanitari Sant Joan de Déu, Sant Boi de Llobregat, Spain, [b]Centro de Investigación Biomédica en Red de Salud Mental, CIBERSAM, Madrid, Spain, [c]Health Service and Population Research Department, Institute of Psychiatry, Psychology and Neuroscience, King's College London, London, United Kingdom

Corresponding author details:

Correspondence: Albert Sanchez- Niubo. Research, Innovation and Teaching Unit, Parc Sanitari Sant Joan de Déu, Dr Antoni Pujadas 42, Sant Boi de Llobregat, Barcelona 08830, Spain. E-mail: albert.sanchez@pssjd.org

ORCID of the authors:

Ivet Bayes-Marin - 0000-0002-3816-5244

Albert Sanchez-Niubo - 0000-0003-0309-181X

Laia Egea-Cortés - 0000-0003-4002-8254

Hai Nguyen - 0000-0003-2171-1955

Matthew Prina - 0000-0001-6698-3263

For peer review only - http://bmjopen.bmj.com/site/about/guidelines.xhtml

Josep Maria Haro - 0000-0002-3984-277X

Beatriz Olaya - 0000-0003-2046-3929

Word count (excluding title page, abstract, references, figures and tables): 4108

Abstract

Objectives: Our aim was to determine clusters of non-communicable diseases (NCDs) in a very large, population-based sample of middle-aged and older adults from low- and middle-income and high-income regions. Additionally, we explored the associations with several covariates.

Design: The total sample was 72 140 people aged 50+ from three population-based studies (ELSA, SHARE and SAGE) included in the ATHLOS project and representing eight regions with high and low income countries. Variables were previously harmonized using an ex-post strategy. Eight NCDs were used in latent class analysis. Multinomial models were made to calculate associations with covariates. All the analyses were stratified by age (50-64 and 65+ years old).

Results: Three clusters were identified: “cardio-metabolic” (8.93% in participants aged 50-64 and 27.22% in those aged 65+), “respiratory-mental-articular” (3.91% and 5.27%) and “healthy” (87.16% and 67.51%). In the youngest group, Russia presented the highest prevalence of the “cardio-metabolic” group (18.8%) and England the “respiratory-mental-articular” (5.1%). In the older group, Russia had the highest proportion of both classes (48.3% and 9%). Both the youngest and older African participants presented the highest proportion of the “healthy” class. Older age, being female, widowed, and with low levels of education and income were related to an increased risk of multimorbidity. Physical activity was a protective factor in both age groups and smoking a risk factor for the “respiratory-mental-articular”.

Conclusions: Multimorbidity is common worldwide, especially in HICs and Russia. Health policies in each country addressing coordination and support are needed to face the complexity of a pattern of growing multimorbidity.

For peer review only - http://bmjopen.bmj.com/site/about/guidelines.xhtml

Keywords: multimorbidity, non-communicable diseases (NCDs), low- and middle- income countries (LMICs), high-income countries (HICs), latent class analysis (LCA).

Strengths and limitations of this study

- This study used a large, harmonized, multi-regional database, which allowed us to compare two age groups (50-64 and 65 or older) as well as disease prevalence and clusters of conditions in regions with differing incomes.
- The presence or absence of the non-communicable diseases was based on self-reported measures, and thus might be affected by measurement errors or lack of accuracy.
- Only diseases that were common across studies were included in the analyses. This might have led to a smaller number of latent classes, or to different patterns of multimorbidity.
- When performing Latent Class Analysis, the 3-class solution was forced as we aimed to do comparisons among age subsamples and regions in terms of disease prevalence as well as protective and risk factors. Invariance analysis should have been performed.
- The use of multiple imputations for missing data in the covariates could carry some bias.

Background

By 2050, the population aged 60 years and older is expected to reach 2 billion worldwide, compared to 900 million in 2015 [1]. Along with this rapid increase, the incidence of chronic conditions or non-communicable diseases (NCDs) is also on the rise, having become the leading cause of morbidity and disability worldwide [2].

Multimorbidity, defined as the co-existence of two or more chronic conditions, is more common in older adults and is often more prevalent in people of lower socioeconomic status [3]. Multimorbidity is thought to account for 65% of total health care expenses in high-income countries (HICs) because of the huge associated healthcare utilization [4]. Due to the increasing prevalence of multimorbidity,

For peer review only - http://bmjopen.bmj.com/site/about/guidelines.xhtml

For peer review only

the managing of multiple conditions has become an unavoidable international research priority, because of the high impact on the quality of life of patients and caregivers and on healthcare systems [3].

Most studies on the prevalence of multimorbidity in older people come from HICs, while data from middle-aged adults and LMICs are much more limited [5–8]. LMICs are experiencing an increase in life expectancy that, together with changes in lifestyle and environment exposures, are triggering changes in their disease burden profile [3,9]. Few studies have compared patterns of multimorbidity between HICs and LMICs. Afshar et al. [10] used population-based chronic disease data from the World Health Survey to compare multimorbidity prevalence across 27 LMICs and one HIC, and used gross domestic product (GDP) to study inter-country socioeconomic differences. They found high multimorbidity prevalence in all the studied countries, and a positive but non-linear relationship between country GDP and multimorbidity prevalence, suggesting the influence of other factors, such as lifestyles, social conditions, and differences among health systems.

The lack of study of differences in multimorbidity between HICs and LMICs may be due to the use of different methodologies, which might hinder comparisons of prevalence and multimorbidity patterns across countries. The integration of data from different studies would allow us to determine differences across regions and cohorts, as well as to explore risk and protective factors involved in the clustering of chronic conditions, thereby improving our understanding of the problem and the creation of adapted medical guidelines.

This study aimed to: a) identify multimorbidity clusters in middle-aged (50-64) and older adults (+65) from different regions, classified as LMICs and HICs; b) investigate the associations between multimorbidity clusters and sociodemographic, economic, lifestyles and health status variables; and c) explore differences across regions.

For peer review only - http://bmjopen.bmj.com/site/about/guidelines.xhtml

Methods

Study design and data extraction

The present study used data from the Ageing Trajectories of Health: Longitudinal Opportunities and Synergies (ATHLOS) project [11]. Longitudinal data from 17 international cohort studies related to health and ageing were harmonized with the aim of obtaining an integrated dataset and achieving a better understanding of ageing and health processes.

We selected three studies due to their inclusion of the variables of interest and the possibility of comparing HICs and LMICs. Baseline samples of the following studies were included in the analyses: the World Health Organization's Study on Global Ageing and Adult Health (SAGE) [12], the English Longitudinal Study of Ageing (ELSA)[13], and the Survey of Health, Ageing and Retirement in Europe Study (SHARE) [14]. These panel studies included non-institutionalized people aged 50 years and older. SAGE comprises six LMICs according to The World Bank Classification [15], namely Ghana, South Africa, Mexico, India, China and Russia; ELSA includes the English population and SHARE covers eleven countries of the European Union and Israel at baseline, considered as HICs [15].

The analyses presented focused on people aged 50 years of age and older who were part of the core sample of each study and who completed a non-proxy interview at baseline. Mexico was excluded from the analyses due the high percentage of missingness (62.08%) in the variables of interest.

Variables

The following variables were the result of a stringent, ex-post harmonization process using systematic harmonization methodology and tools from Maelstrom Research [16].

Eight NCDs were used to conduct the analysis, including those that were available in the three studies: diabetes, hypertension, asthma, chronic lung disease, joint disorders (arthritis, rheumatism or osteoarthritis), angina or myocardial infarction, stroke, and depression. The presence or absence of these conditions was self-reported and based on a medical diagnosis. Depression was assessed with standardized tools, such as the Composite International Diagnostic Interview (CIDI) in the SAGE

For peer review only - http://bmjopen.bmj.com/site/about/guidelines.xhtml

study, the Center for Epidemiologic Studies Depression Scale (CES-D) in ELSA, and the EURO-D in SHARE [17–19]. A dichotomous variable (yes/no) was created using the proper cut-off score for each tool and population.

Self-reported demographic variables included age, gender, level of education (primary or less, secondary, and tertiary), marital status (single, married or currently cohabiting, separated or divorced, and widowed) and quintiles of household wealth (first quintile indicating lowest level). Life-styles and health behaviors were 'ever smoked' any type of tobacco and physical activity referring to the practice of vigorous exercise during the last two weeks, both coded as *yes* or *no*. Other health related variables were self-rated health (good, moderate, or poor), presence or absence of loneliness feelings in the last week, difficulties in activities of daily living (ADL), cognitive performance, and number of diseases.

To assess difficulties in ADL, we used a set of daily self-care activities such as problems in using the toilet, bathing or showering, getting dressed, eating, moving, or getting in or out of bed. Each of the ADL difficulties was coded into a *yes/no* if the person answered 'severe' or 'extreme/cannot do it'. To build the set of ADL difficulties, we coded *yes* if the person reported at least one difficulty in any of the five items.

Immediate and delayed recall was assessed using the 10-word learning list task, and verbal fluency utilizing the animal naming test [20]. Continuous total scores were used to perform the analyses. Number of diseases was built by adding up the occurrences of all the above-mentioned NCDs.

Finally, a 7-level regional membership variable was created in order to analyze regional differences, based on the World Health Organization (WHO) and the United Nations Statistical Division (UNSD) regional classification [21,22]. Moreover, the World Bank Classification was used to classify these regions into HICs or LMICs [15]. SAGE includes Africa (Ghana and South Africa), China and India, all of them considered as LMICs. SHARE countries were grouped into three regions: Northern Europe (Denmark, Sweden), Southern Europe (Greece, Italy, and Spain) and Western Europe (Austria, Belgium, France, Germany, Israel, Netherlands, and Switzerland). ELSA and SHARE regions were

For peer review only - http://bmjopen.bmj.com/site/about/guidelines.xhtml

considered as HICs. Ghana and South Africa were grouped together and named as Africa for practical purposes as well as due to their smaller sample size. These countries are not necessarily representative of the whole continent.

Statistical analysis

Descriptive statistics were used to summarize information regarding sociodemographic economic variables and disease prevalence among regions. Confidence intervals (IC95%) were calculated for categorical variables in order to make comparisons across regions.

Latent Class Analysis (LCA) was conducted stratified by age (50-64, +65). Eight NCDs (diabetes, hypertension, asthma, chronic lung disease, joint disorders, angina-myocardial infarction, stroke, and depression) were used as observed indicators. Region was used as cluster when conducting LCA in order to accurately describe disease proportions, indicating that the subjects were not independent random draws, but rather were nested within clusters [23] .

The optimal number of latent classes was determined using the adjusted Bayesian Information Criterion (aBIC), the consistent Akaike Information Criterion (CAIC), and the Entropy Index. Lower values of aBIC and CAIC indicate better fit, whereas entropy index values higher than 0.80 indicate that the latent classes are highly discriminating [24]. The average posterior probability indicates how well a model classifies individuals into their most likely class. Values higher than 0.70 indicate well-identified classes [25]. Additionally, interpretability and clinical judgment were used.

Missing data in one of the indicators was handled with the full-information maximum likelihood (FIML) technique, assuming missing-at-random (MAR) [26]. Missing data in the covariates was handled using multiple imputation by chained equations (MICE) assuming missing-at-random (MAR) [26]. The imputation model included the outcome (group membership in one of the latent classes) and all the variables used in the regression models (See Additional file 1: Table S1).

Multinomial logistic regression models were used to assess the association of each multimorbidity class with several outcomes adjusted for gender, age, marital status, education level, wealth, and

For peer review only - http://bmjopen.bmj.com/site/about/guidelines.xhtml

region. Regression models were conducted separately in one hundred imputed datasets and results combined using Rubin's rules [27].

All analyses were conducted with Stata SE version 13.1 (College Station, TX). LCA analyses were performed using a Stata plugin [23].

Results

Descriptive analysis

People with missing values in sex and age were excluded, resulting in a final sample of 72 140 individuals aged 50 years old (Table 1). The mean age ranged from 62 (SD=9.02) in Southern Asia to 65 years (10.18) in Russia and (10.26) in England. Some 54% were females, 72% were married or cohabitating, and 39% had secondary education. Russia presented the highest number of conditions (mean 1.66) compared to Africa (0.64), China (0.80), and India (0.72).

The most prevalent conditions in the total sample were hypertension (31.2%, 95%CI = 30.9-31.6) and joint disorders (22.4%, 95%CI = 22.0-22.7).

Hypertension was particularly high in Russia (56.5%, 95%CI = 54.9-58.1) compared to the other regions. Diabetes prevalence was greater in Southern (11.9%, 95%CI = 11.2-12.7) and Western Europe (10.4%, 95%CI = 9.9-10.9), whereas Africa and China presented the lowest proportions. Similarly, myocardial infarction–angina was highly prevalent in Russia (33.1%, 95%CI = 31.6-34.6), followed by countries of Northern (13.8%, 95%CI = 12.8-14.8), Southern (11.7%, 95%CI = 11.0-12.4) and Western Europe (13.1%, 95%CI =12.6-13.6); England, Africa, and India presented the lowest proportion of diabetes.

For peer review only - http://bmjopen.bmj.com/site/about/guidelines.xhtml

Table 1 Main characteristics of the total sample and by regions

Region	Total	Africa[a]	China[b]	India[b]	Russia[b]	England[c]	Northern Europe[d]	Southern Europe[e]	Western Europe[f]
	N=72140	n=7950	n=12840	n=6558	n=3887	n=11 517	n=4573	n=7465	n=17 350
**Age, mean (SD)**	64.05 (9.96)	63.60 (10.26)	63.07 (9.31)	61.80 (9.02)	65.06 (10.18)	65.06 (10.26)	64.78 (10.34)	65.02 (10.17)	64.34 (9.96)
**Female, % (IC 95%)**	54.0 (53.6-54.4)	52.3 (51.2-53.4)	52.9 (52.1-54.8)	49.6 (48.4-50.8)	64.6 (63.0-66.1)	54.6 (53.7-55.5)	53.2 (51.8-54.7)	55.4 (54.2-56.5)	54.1 (53.4-54.9)
**Marital status, % (IC 95%)**									
Single	4.1 (4.0-4.3)	6.7 (6.2-7.3)	0.9 (0.7-1.6)	1.0 (0.8-1.2)	2.8 (2.3-3.4)	5.0 (4.6-5.4)	5.5 (4.8-6.2)	6.3 (5.8-6.9)	4.9 (4.6-5.3)
Married	71.5 (71.1-72.0)	54.5 (53.4-55.6)	83.5 (82.8-84.1)	74.1 (73.1-75.2)	56.1 (54.5-57.7)	69.1 (68.2-69.9)	72.4 (71.0-73.7)	73.3 (72.3-74.3)	73.3 (72.7-74.0)
Divorced	5.9 (5.7-6.2)	10.4 (9.7-11.1)	1.7 (1.5-2.0)	0.6 (0.5-0.9)	8.3 (7.4-9.2)	9.0 (8.5-9.5)	9.6 (8.8-10.5)	2.6 (2.3-3.0)	6.8 (6.5-7.2)
Widowed	18.4 (18.1-18.6)	27.3 (26.3-28.3)	13.9 (13.3-14.6)	24.3 (23.2-25.3)	32.7 (31.2-34.2)	16.9 (16.3-17.6)	12.5 (11.6-13.5)	17.8 (16.9-18.6)	14.9 (14.4-15.4)
**Education level % (IC 95%)**									
Primary or less	33.4 (33.0-33.7)	30.5 (29.5-31.5)	37.9 (37.1-38.8)	25.5 (24.5-26.6)	9.9 (9.0-10.9)	42.4 (41.5-43.4)	29.6 (28.3-30.9)	59.4 (58.3-60.5)	23.2 (22.6-23.8)
Secondary	38.7 (38.4-39.1)	19.0 (18.2-19.9)	33.2 (32.4-34.0)	18.2 (17.3-19.2)	69.3 (67.9-70.8)	37.5 (36.7-38.4)	45.1 (43.6-46.5)	31.3 (30.2-32.3)	55.1 (54.3-55.8)
Tertiary	12.0 (11.9-12.2)	3.9 (3.5-4.3)	4.7 (4.3-5.0)	5.0 (4.4-5.5)	19.7 (18.5-21.0)	11.1 (10.6-11.7)	24.2 (23.0-25.5)	9.1 (8.4-9.7)	20.8 (20.2-21.4)
**Wealth (quintiles% (IC 95%)**									
1st (worse)	19.3 (19.1-19.6)	19.3 (18.4-20.2)	19.8 (19.1-20.5)	16.2 (15.3-17.1)	18.2 (17.0-19.5)	19.0 (18.3-19.7)	20.7 (19.6-21.9)	20.3 (19.4-21.2)	19.9 (19.3-20.5)
2nd	19.7 (19.4-20.0)	19.7 (18.4-20.2)	19.7 (19.0-20.4)	18.6 (17.6-19.5)	19.8 (18.5-21.1)	19.3 (18.6-20.1)	20.4 (19.3-21.6)	20.4 (19.5-21.3)	19.9 (19.4-20.6)
3rd	19.7 (19.4-19.9)	19.8 (18.9-20.7)	20.1 (19.4-20.8)	18.4 (17.5-19.4)	20.3 (19.0-21.6)	19.7 (18.9-20.4)	20.1 (19.0-21.3)	19.8 (18.9-20.7)	19.5 (18.9-20.1)
4th	19.9 (19.7-20.3)	20.5 (19.6-21.4)	20.5 (19.8-21.2)	21.5 (20.5-22.5)	20.0 (18.8-21.3)	19.6 (18.9-20.3)	19.7 (18.5-20.8)	19.4 (18.6-20.4)	19.3 (18.8-19.9)
5th (best)	20.1 (19.8-20.4)	20.4 (19.5-21.3)	19.7 (19.0-20.4)	24.8 (23.8-25.9)	21.6 (20.3-22.9)	19.6 (18.9-20.3)	18.9 (17.8-20.1)	18.9 (18.0-19.8)	19.4 (18.8-20.0)
**Nº diseases, *mean (SD)***	1.02 (1.14)	0.64 (0.94)	0.80 (0.99)	0.72 (0.97)	1.66 (1.38)	1.19 (1.13)	1.02 (1.10)	1.28 (1.25)	1.10 (1.16)
**Diseases, % (IC 95%)**									
Diabetes	8.5 (8.3-8.7)	6.6 (6.1-7.2)	6.5 (6.1-7.0)	7.3 (6.7-7.9)	9.0 (8.1-10.0)	7.4 (6.9-7.9)	8.2 (7.4-9.0)	11.9 (11.2-12.7)	10.4 (9.9-10.9)
Hypertension	31.2 (30.9-31.6)	21.5 (20.6-22.4)	27.4 (26.6-28.2)	17.5 (16.6-18.5)	56.5 (54.9-58.1)	37.8 (36.9-38.7)	29.3 (28.0-30.7)	35.6 (34.5-36.7)	32.3 (31.6-33.0)
Joint disorders	22.4 (22.0-22.7)	17.8 (16.9-18.6)	22.1 (21.4-22.8)	17.9 (17.0-18.9)	35.2 (33.7-36.7)	32.5 (31.6-33.3)	15.7 (14.6-16.8)	26.3 (25.3-27.3)	16.8 (16.3-17.4)
Asthma	5.5 (5.3-5.6)	4.1 (3.7-4.5)	2.4 (2.2-2.7)	6.9 (6.3-7.6)	3.4 (2.9-4.0)	11.7 (11.1-12.3)	7.6 (6.8-8.4)	4.1 (3.7-4.6)	4.1 (3.8-4.4)
Chronic lung disease	6.1 (5.9-6.3)	1.5 (1.2-1.7)	8.6 (8.1-9.1)	4.1 (3.6-4.6)	17.9 (16.8-19.2)	6.5 (6.1-7.0)	4.5 (3.9-5.2)	5.4 (4.9-6.0)	4.9 (4.6-5.3)
MI - Angina	10.0 (9.8-10.3)	4.5 (4.1-5.0)	8.8 (8.3-9.3)	4.9 (4.4-5.5)	33.1 (31.6-34.6)	3.3 (3.0-3.6)	13.8 (12.8-14.8)	11.7 (11.0-12.4)	13.1 (12.6-13.6)
Stroke	3.8 (3.7-3.9)	3.2 (2.8-3.6)	3.5 (3.2-3.8)	2.2 (1.9-2.6)	6.0 (5.3-6.8)	4.5 (4.1-4.9)	5.0 (4.4-5.7)	3.1 (2.8-3.6)	3.9 (3.7-4.2)
Depression	15.3 (15.0-15.5)	5.8 (5.3-6.3)	1.2 (1.1-1.4)	12.1 (11.3-12.9)	5.2 (4.5-5.9)	16.5 (15.8-17.2)	18.8 (17.7-20.0)	31.7 (30.6-32.7)	25.0 (24.3-25.6)

Note. [a]SAGE study - Africa: Ghana, South Africa; [b]SAGE study; [c]ELSA study- England; [d]SHARE study - Northern Europe: Denmark, Sweden; [e]SHARE study - Southern Europe: Greece, Italy, Spain; [f]SHARE study - Western Europe: Austria, Belgium, France, Germany, Israel, Netherlands, Switzerland.

The analyses were performed before multiple imputation procedure

For peer review only

For peer review only - http://bmjopen.bmj.com/site/about/guidelines.xhtml

Joint disorders were more prevalent in Russia (35.2%, 95%CI = 33.7-36.7) and England (32.5%, 95%CI =31.6-33.3). The prevalence of asthma was greater in England than other regions (11.7%, 95%CI = 11.1-12.3) and chronic lung disease was greater in Russia (17.9%, 95%CI = 16.8-19.2).

As for the prevalence of depression, European countries presented the highest values, especially in Southern (31.7%, 95%CI = 30.6-32.7) and Western Europe (25.0%, 95%CI = 24.3-25.6), whereas LMICs showed very low proportions, especially in China, where only 1.2% of people aged 50+ presented depression.

Multimorbidity patterns

Table 2 displays the aBIC, CAIC, and entropy values, proportions, and average posterior probability of each latent class, for a two- to five-class model in both age subsamples. In the younger subsample (50-64), the five-class solution yielded the lowest aBIC and CAIC values and the highest entropy value (0.67). However, it was dismissed because one of the latent classes was very infrequent and the posterior probabilities were far below .70. Similarly, the four-class model was rejected for an inadequate posterior probability value in one of the classes (0.52). The model finally selected was the 3-class model. The three-class solution was also chosen for the older age group because of lower posterior probability values in the four- and five-class models in spite of lower aBIC and CAIC values.

We named each latent class according to the most prevalent diseases within each latent class. Figure 1 shows the distribution of each condition across latent classes in the total sample and by regions. The "cardio-metabolic" class presented excess prevalence of diabetes, hypertension, myocardial infarction or angina, and stroke, comprising 8.93% of the total sample in the younger group and 27.22% in the older group. The "respiratory-mental-articular" class, which comprised 3.91% and 5.27% of each sample, respectively, showed greater prevalence of joint disorders, asthma, chronic lung diseases, and depression. Finally, the "healthy" class presented low prevalence of conditions, comprising 87.16% of the sample in the first age group and 67.51% in the second group.

For peer review only - http://bmjopen.bmj.com/site/about/guidelines.xhtml

Table 2 Comparison between models in individuals aged 50-64 and +65

	Aged 50-64					Aged ≥65				
No. of latent classes	Information Criteria Indices		Classification Quality	Latent classes, n (%)	Average posterior probability	Information Criteria Indices		Classification Quality	Latent classes, n (%)	Average posterior probability
	aBIC	CAIC	Entropy			aBIC	CAIC	Entropy		
2	1512.91	1583.94	0.51	33023 (82.15)<br>7177 (17.85)	.88<br>.75	1603.30	1674.33	0.39	21113 (66.10)<br>10827 (33.90)	.83<br>.75
**3**	**875.23**	**983.86**	**0.63**	**3589 (8.93)**<br>**1571 (3.91)**<br>**35040 (87.16)**	**.76**<br>**.67**<br>**.87**	**1032.83**	**1141.46**	**0.50**	**8693 (27.22)**<br>**1684 (5.27)**<br>**21563 (67.51)**	**.71**<br>**.68**<br>**.81**
4	777.14	923.37	0.43	25701 (63.93)<br>626 (1.56)<br>7754 (19.29)<br>6119 (15.22)	.52<br>.72<br>.80<br>.68	817.60	963.83	0.63	9557 (29.92)<br>1474 (4.61)<br>17220 (53.91)<br>3689 (11.55)	.65<br>.78<br>.86<br>.77
5	661.04	844.87	0.67	4578 (11.39)<br>247 (0.61)<br>32423 (80.65)<br>1359 (3.38)<br>1593 (3.96)	.64<br>.67<br>.85<br>.48<br>.64	689.22	873.05	0.59	11094 (34.73)<br>1094 (3.43)<br>14155 (44.32)<br>1148 (3.59)<br>4449 (13.93)	.77<br>.71<br>.76<br>.63<br>.72

Note: Boldface indicates the final selected model. *aBIC* adjusted Bayesian Information Criterion, *CAIC* Consistent Akaike Information Criterion

For peer review only

For peer review only - http://bmjopen.bmj.com/site/about/guidelines.xhtml

Differences in the proportions of multimorbidity classes were found across regions (Figure 1). The "cardio-metabolic" class (18.8%, 95%CI =17.1-20.6) was significantly greater in Russia than in other regions, and England (5.1%, 95%CI =4.5-5.7) showed a higher proportion of individuals classified into the "respiratory-mental-articular" class. The "healthy" class was higher in Africa (91.5%, 95%CI = 90.7-92.3), China (90.8%, 95%CI = 90.1-91.4), and India (89.5%, 95%CI = 88.5-90.4), and remarkably lower in Russia (71.6%, 95%CI = 69.5-73.6) compared to other regions.

----- FIGURE 1 HERE -----

Similar results were found for the older group (Figure 2). In Russia, the "cardio-metabolic" class was significantly higher than in other regions (48.3%, 95%CI = 46.1-50.6) whereas the "healthy" class was the least frequent class compared with the rest of regions (38.4%, 95%CI = 36.2-40.6), followed by Southern Europe (52.6%, 95%CI = 50.9-54.2). Africa and India showed lower proportions of individuals classified into the "cardio-metabolic" class (12.9%, 95%CI = 11.8-14.1 and 11.2%, 95%CI = 10.0-12.6, respectively).

----- FIGURE 2 HERE -----

Association between multimorbidity classes and covariates

Table 3 shows the unadjusted and adjusted relative risk ratios (RRR) for the younger sample. The "healthy" class was used as the reference group. Both multimorbidity classes (cardio-metabolic and respiratory-mental-articular) were associated with all the covariates, except for smoking status in the "cardio-metabolic" class. Compared with the "healthy" class, individuals classified into the "cardio-metabolic" and "respiratory-mental-articular" classes were more likely to be older (RRR = 1.09, 95%CI = 1.08-1.10; RRR = 1.06, 95%CI = 1.04-1.07, respectively) and being widowed (RRR = 1.4, 95%CI = 1.1-1.7) and divorced in the "respiratory-mental-articular" class (RRR = 1.7, 95%CI = 1.2-2.4). Being

For peer review only - http://bmjopen.bmj.com/site/about/guidelines.xhtml

a male, having tertiary education, and high levels of wealth had a protective effect for being in both multimorbidity groups, compared with the "healthy" class.

Taking Africa as the reference category, participants from Russia were more likely to be classified into the "cardio-metabolic" class (RRR = 3.6, 95%CI = 3.0-4.2), whereas individuals from England (RRR = 5.6, 95%CI = 4.1-7.7), Northern Europe (RRR = 2.8, 95%CI = 1.9-4.1) and India (RRR = 2.2, 95%CI = 1.5-3.2) showed higher risk of being in the "respiratory-mental-articular" class.

After adjusting for covariates, both latent classes were more likely to be associated with the presence of feelings of loneliness (RRR = 1.8, 95%CI = 1.7-2.0,; RRR = 2.5, 95%CI = 2.0-3.0), limitations in ADL (RRR = 3.2, 95%CI = 2.9-3.6; RRR = 3.9, 95%CI = 3.3-4.7) and worse health status (RRR = 12.8, 95%CI = 11.3-14.4; RRR = 12.9, 95%CI = 10.5-16.0). Physical activity had a protective effect for being in these classes and having smoked was a risk factor only for being classified into the "respiratory-mental-articular" class (RRR = 1.5, 95%CI = 1.2-1.7). Better performance in verbal memory was significantly associated with less risk of being classified into the two multimorbidity classes. Similarly, higher scores in verbal fluency (RRR = 0.96, 95%CI = 0.96-0.97; RRR = 0.89, 95%CI = 0.97-0.99) were a protective factor for multimorbidity, compared with the healthy individuals group.

For peer review only

For peer review only - http://bmjopen.bmj.com/site/about/guidelines.xhtml

Table 3 Association between latent multimorbidity membership and outcomes in individuals aged 50-64

	Aged 50-64			
	Unadjusted		Adjusted[a]	
Outcomes	"Cardio-metabolic" class	"Respiratory-mental-articular" class	"Cardio-metabolic" class	"Respiratory-mental-articular" class
Gender				
Female	1.0	1.0		
Male	0.9 (0.8-1.0)	0.6 (0.5-0.7)		
Age (years)	1.1 (1.1-1.1)	1.1 (1.0-1.1)		
Marital status				
Single	1.0	1.0		
Married	0.9 (0.8-1.1)	0.7 (0.5-1.0)		
Divorced	1.1 (0.9-1.4)	1.7 (1.2-2.4)		
Widowed	1.4 (1.1-1.7)	1.1 (0.8-1.6)		
Education level				
Primary or less	1.0	1.0		
Secondary	0.9 (0.9-1.0)	0.7 (0.6-0.8)		
Tertiary	0.8 (0.7-0.9)	0.6 (0.5-0.7)		
Wealth				
1st (worse)	1.0	1.0		
2nd	1.0 (0.9-1.1)	0.8 (0.6-1.0)		
3rd	0.9 (0.9-1.1)	0.7 (0.5-0.8)		
4th	0.9 (0.8-1.0)	0.6 (0.5-0.8)		
5th (best)	0.8 (0.7-0.9)	0.5 (0.4-0.6)		
Region				
Africa	1.0	1.0		
China	0.9 (0.8-1.0)	0.3 (0.2-0.5)		
India	0.9 (0.8-1.1)	2.2 (1.5-3.2)		
Russia	3.6 (3.0-4.2)	2.2 (1.4-3.5)		
England	1.1 (0.9-1.3)	5.6 (4.1-7.7)		
Northern Europe	1.3 (1.1-1.5)	2.8 (1.9-4.1)		
Southern Europe	1.5 (1.3-1.8)	1.7 (1.2-2.5)		
Western Europe	1.5 (1.3-1.7)	1.9 (1.3-2.6)		
Loneliness (yes/no)	1.9 (1.8-2.1)	2.5 (2.1-3.0)	1.8 (1.7-2.0)	2.5 (2.0-3.0)
Ever smoked (yes/no)	1.0 (0.9-1.1)	1.7 (1.5-2.0)	1.1 (1.0-1.2)	1.5 (1.2-1.7)
Physical activity (yes/no)	0.5 (0.5-0.5)	0.6 (0.5-0.7)	0.4 (0.4-0.5)	0.5 (0.5-0.6)
Limitations in ADL (yes/no)	2.5 (2.3-2.7)	3.2 (2.7-3.7)	3.2 (2.9-3.6)	3.9 (3.3-4.7)
Self-rated health				
Good	1.0	1.0	1.0	1.0
Moderate	3.7 (3.3-3.9)	2.5 (2.1-2.9)	4.8 (4.3-5.2)	3.9 (3.2-4.7)
Poor	8.5 (7.7-9.4)	7.4 (6.1-8.8)	12.8 (11.3-14.4)	12.9 (10.5-16.0)
Memory: Immediate recall	0.9 (0.9-0.9)	0.9 (0.9-1.0)	0.9 (0.9-0.9)	0.9 (0.9-1.0)
Memory: Delayed recall	0.9 (0.9-0.9)	0.9 (0.8-0.9)	0.9 (0.9-0.9)	0.9 (0.9-1.0)
Verbal fluency	1.0 (1.0-1.0)	1.0 (1.0-1.0)	1.0 (1.0-1.0)	1.0 (1.0-1.0)

Note: The reference group for the multimorbidity group variable was the "Healthy" class
Relative Risk Ratios (95% confidence interval) from multinomial logistic regression models
Models were run in 100 imputed datasets and results combined using Rubin's rules
ADL Activities of Daily Living
[a]Adjusted for gender, age, marital status, education level, wealth and region

For peer review only - http://bmjopen.bmj.com/site/about/guidelines.xhtml

Table 4 shows the RRR for the older subsample. Individuals in lower wealth quintiles had greater risk of being classified into the "cardio-metabolic" class (RRR = 1.1, 95%CI = 1.0-1.2), and individuals who were in the fourth and fifth quintile (RRR = 0.8, 95%CI = 0.7-1.0) were less likely to be classified into the "respiratory-mental-articular" class, compared with the "healthy" group. Compared with African participants, all regions had greater risk of being classified into the "respiratory-mental-articular" class, compared to the "healthy" class, especially participants from Russia (RRR = 14.5, 95%CI = 10.3-20.3), compared to Africa.

Likewise, individuals who had ever smoked had a higher risk of being in the "respiratory-mental-articular" group (RRR = 1.8, 95%CI = 1.5-2.0) and a lower risk of being classified into the "cardio-metabolic" class (RRR = 1.0, 95%CI = 0.9-1.0). Better scores in verbal memory and verbal fluency were significantly associated with less risk of being classified into the multimorbidity groups compared with the class of the "healthy" individuals.

Discussion

To the best of our knowledge, this is the first multi-region study to use harmonized data to compare multimorbidity patterns across different regions from three distinct population-based cohorts. We identified three latent classes of multimorbidity based on the presence of eight NCDs: the "cardio-metabolic", the "respiratory-mental-articular" and the "healthy" class. The same clusters were identified in another study using SAGE original data, applying exploratory factor analysis (EFA) in a sample of 41 909 individuals aged 50 years or older [28]. Similarly, a study of a representative sample of Spanish community-dwelling adults over 50 years old also found three latent classes using eleven chronic conditions, showing similar diseases distributions among the multimorbidity clusters [29].

For peer review only - http://bmjopen.bmj.com/site/about/guidelines.xhtml

Table 4 Association between latent multimorbidity membership and outcomes in individuals aged ≥65

Outcomes	Aged ≥65			
	Unadjusted		Adjusted[a]	
	"Cardio-metabolic" class	"Respiratory-mental-articular" class	"Cardio-metabolic" class	"Respiratory-mental-articular" class
Gender				
Female	1.0	1.0		
Male	0.7 (0.7-0.8)	0.9 (0.8-1.0)		
Age (years)	1.0 (1.0-1.0)	1.0 (1.0-1.0)		
Marital status				
Single	1.0	1.0		
Married	1.2 (1.00-1.4)	1.2 (0.9-1.6)		
Divorced	1.1 (1.0-1.4)	1.8 (1.2-2.6)		
Widowed	1.5 (1.3-1.8)	1.6 (1.2-2.3)		
Education level				
Primary or less	1.0	1.0		
Secondary	1.0 (0.9-1.0)	0.7 (0.6-0.8)		
Tertiary	0.9 (0.8-1.0)	0.6 (0.5-0.7)		
Wealth				
1st (worse)	1.0	1.0		
2nd	1.1 (1.0-1.2)	1.1 (0.9-1.2)		
3rd	1.1 (1.0-1.2)	1.0 (0.9-1.2)		
4th	1.1 (1.0-1.2)	0.8 (0.7-1.0)		
5th (best)	1.2 (1.0-1.2)	0.7 (0.6-0.9)		
Region				
Africa	1.0	1.0		
China	1.8 (1.6-2.1)	4.1 (3.0-5.7)		
India	0.9 (0.8-1.0)	2.9 (2.0-4.2)		
Russia	8.0 (7.0-9.2)	14.5 (10.3-20.3)		
England	2.2 (2.0-2.5)	6.2 (4.5-8.5)		
Northern Europe	1.9 (1.6-2.2)	4.2 (2.9-6.1)		
Southern Europe	2.8 (2.5-3.2)	6.5 (4.7-9.0)		
Western Europe	2.1 (1.9-2.3)	3.6 (2.6-5.0)		
Loneliness (yes/no)	1.4 (1.4-1.6)	1.9 (1.7-2.2)	1.3 (1.2-1.5)	1.9 (1.7-2.3)
Ever smoked (yes/no)	0.8 (0.8-0.8)	1.4 (1.3-1.6)	1.0 (0.9-1.0)	1.8 (1.5-2.0)
Physical activity (yes/no)	0.5 (0.5-0.6)	0.5 (0.4-0.6)	0.5 (0.5-0.6)	0.4 (0.4-0.5)
Limitations in ADL (yes/no)	1.8 (1.7-1.9)	2.8 (2.5-3.1)	2.3 (2.1-2.5)	4.0 (3.5-4.6)
Self-rated health				
Good	1.0	1.0	1.0	1.0
Moderate	2.5 (2.3-2.7)	3.9 (3.3-4.6)	3.1 (2.9-3.4)	5.7 (4.8-6.8)
Poor	4.3 (4.6)	10.9 (9.3-12.8)	6.2 (5.6-6.9)	19.4 (16.2-23.4)
Memory: Immediate recall	0.9 (0.9-0.9)	0.9 (0.9-0.9)	0.9 (0.9-0.9)	0.9 (0.9-1.0)
Memory: Delayed recall	0.9 (0.9-0.9)	0.9 (0.9-0.9)	0.9 (0.9-0.9)	0.9 (0.9-1.0)
Verbal fluency	1.0 (0.9-1.00)	1.0 (0.9-1.0)	0.9 (0.9-1.0)	1.0 (1.0-1.0)

Note: The reference group for the multimorbidity group variable was the "Healthy" class
Relative Risk Ratios (95% confidence interval) from multinomial logistic regression models
Models were run in 100 imputed datasets and results combined using Rubin's rules
ADL Activities of Daily Living
[a]Adjusted for gender, age, marital status, education level and wealth

For peer review only - http://bmjopen.bmj.com/site/about/guidelines.xhtml

In our study, for both age groups the majority of the sample was classified into the "healthy" class, 87.16% and 67.51%, respectively. This latent group has previously been described in studies which applied LCA [29–32]. Likewise, the other two identified classes are similar to those reported in a systematic review based on 14 studies of multimorbidity patterns [33]. In this review, the most prevalent diseases in the "cardio-metabolic" group were diabetes, hypertension, heart diseases, hyperlipidemia, and obesity; and in the second group conditions such as mental disorders, thyroid disease, neurological disease, pain, asthma or chronic lung diseases, musculoskeletal disorders, obesity, and gastroesophageal reflux disease were included. Despite the fact that we included a smaller number of diseases, we found analogous patterns. In our study, 8.93% of the younger group (50-64) and 27.22% of the older were classified into the "cardio-metabolic" class, including individuals with higher prevalence of diabetes, hypertension, myocardial infarction or angina, and stroke. This clustering of diseases is similar to the metabolic syndrome, which has metabolically-related cardiovascular risk factors and greater risk of stroke and diabetes [34]. Lastly, the least prevalent group was the "respiratory-mental-articular" class, consisting of greater prevalence of joint disorders, asthma, chronic lung diseases, and depression. Association between depression and arthritis has commonly been reported, with socioeconomic and disease factors reported as being involved in its association, as well as systemic inflammation mechanisms [3]. Nevertheless, the links between depression and chronic lung diseases, and chronic lung diseases and arthritis, despite having been studied, remain unclear [34,35].

Analogous latent multimorbidity classes have been found among both age groups. Despite this, certain aspects should be pointed out. As expected, the proportion of participants classified into the "healthy" class was greater in participants aged 50-64 (87.16%) compared to those aged +65 years old (67.51%), illustrating higher multimorbidity in elderly individuals. The distribution of chronic conditions was also less clear in the older subsample. For example, both joint disorders and angina–myocardial infarction were similarly present in the "cardio-metabolic" and "respiratory-mental-

For peer review only - http://bmjopen.bmj.com/site/about/guidelines.xhtml

articular" categories, whereas in the youngest participant (50-64) subsample we observed a more differentiated profile of those chronic conditions that cluster into one latent class. For example, respiratory-related diseases (asthma, chronic lung diseases) are highly presented in the "respiratory-mental-articular" class, while very infrequent among middle-aged people classified into the "cardio-metabolic" group. It is worth mentioning that although depression is frequently observed among participants classified into the "respiratory-mental-articular" class, it is not infrequent among people within the "cardio-vascular" class. This may be due to the relationship between mental and medical disorders, which has frequently been reported, suggesting a bidirectional association between them [36]. On the one hand, medical conditions could be accompanied by a high symptom burden, leading to depression, and, on the other, depression could be a risk factor for medical conditions, since depressive symptoms could increase the incidence of behaviors, such as smoking, alcohol intake, poor diet, or physical inactivity, which are risk factors for NCDs [3,36].

One important implication of our findings is the relatively high proportion of people aged 50-64 with multimorbidity. Thus, preventive and intervention programs are also needed for this population to mitigate the multimorbidity burden.

Our results show that these multimorbidity patterns are qualitatively different, but only if compared to the "healthy" class in terms of sociodemographic and economic characteristics, lifestyles, and health status variables. As has been reported in the literature, being older, female, widowed, with a lower level of education and lower socioeconomic status are related to an increased risk of multimorbidity [3]. In addition, those individuals with multiple chronic conditions were more likely to have limitations in ADL, especially those classified into the "respiratory-mental-articular" group, similar to what was found in another study of multimorbidity [29]. Physical activity seems to be a protective factor for being classified into the "respiratory-mental-articular" class, whereas smokers were more likely to be classified into the "respiratory-mental-articular" class, but not the "cardio-metabolic" class. This is inconsistent with the literature, since cigarette smoking is considered a

For peer review only - http://bmjopen.bmj.com/site/about/guidelines.xhtml

major cause of cardiovascular diseases (CVDs). However, smoking is probably the most complex and least understood risk factor for CVDs [37].

One interesting finding is the association between cognition outcomes and multimorbidity in both age subsamples. Better performance in verbal memory and fluency was related to less risk of being classified into the multimorbidity groups, with similar results among latent classes. Impaired cognition has been associated with conditions such as arthritis [38], depression [39], and respiratory diseases [40], cardiovascular conditions, diabetes [41], hypertension [42], and coronary heart diseases [43].

Concerning the regional distribution of multimorbidity, Russia accounted for the highest burden as opposed to Africa, China, and India. The "cardio-metabolic" class is especially common in this country, with a prevalence of 18.82% in the youngest and 48.34% in the older subsample. Prevalence of CVDs, such as hypertension, myocardial infarction or angina, and stroke, was also higher in Russia. This high proportion could be related to the high rate of alcohol consumption and rapid societal changes experienced in this country which might account for increased risk of circulatory diseases [44,45]. Followed by Russia, European regions showed higher rates of multimorbidity. NCDs such as hypertension, joint disorders, respiratory diseases, and depression were highly prevalent, especially in England and Southern Europe, where the "respiratory-mental-articular" class was highly prevalent in both age subsamples. The relationship between mood disorders such as depression and joint disorders has been previously reported in other studies, though the underlying cause remains unclear [28,29,33]. Notwithstanding, previous studies suggested that the emotional burden of joint disorders may contribute to the onset of psychiatric disorders [28,46].

LMICs such as Africa, China, and India showed lower rates of multimorbidity compared to Russia and other HICs. However, there was a wide variation in terms of some diseases, such as respiratory diseases and depression. Asthma and chronic lung diseases were highly prevalent in India and China, influenced by factors such as increasing smoking rates, air pollution, and occupational lung diseases

For peer review only - http://bmjopen.bmj.com/site/about/guidelines.xhtml

in these countries [47]. As reported in previous studies [48], depression was remarkably prevalent in India, whereas the lowest prevalence was observed in China. This is in line with previous epidemiological studies on the prevalence of depression in Chinese older people, suggesting differences in diagnostic criteria that make depression less diagnosed; somatic symptoms are more prevalent in this population instead of sadness, and lack of interest and energy. Moreover, stigma and prejudice in Chinese population might also contribute to under-reporting depressive symptoms [49,50].

The highest burden of multimorbidity in HICs could be explained by an increased level of development in the HICs. Notwithstanding, LMICs are experiencing a change in lifestyle and environmental exposures which contributes, as in HICs, to multimorbidity. Thus, the increased burden of NCDs, in addition to the existing burden of infectious diseases such as HIV/AIDS, worsens multimorbidity management [3] .

Strengths and limitations

A major strength of this study is the use of a large, harmonized, multi-regional database. Research on multimorbidity has typically been hampered by several factors, such as the exclusion of patients with multimorbidity from participation, targeting of research mostly on elderly individuals, and a shortage of studies focusing on LMICs. The ATHLOS study allowed us to compare two age groups (50-64 and 65 or older) as well as disease prevalence and clusters of conditions in regions with differing incomes in a very large, diverse population-based study of middle-aged and older adults.

Some limitations should be considered when interpreting our findings. First, the presence or absence of the NCDs was based on self-reported measures, and thus might be affected by measurement errors or lack of accuracy. Nevertheless, some authors sustain self-reported diagnostics as a well-established method for the measurement of multimorbidity in population-based studies [51–54]. Second, we could only focus on those diseases that were common across studies. Conditions such as

For peer review only - http://bmjopen.bmj.com/site/about/guidelines.xhtml

obesity, cancer, kidney disease, and neurological illness were not evaluated. This might have led to a smaller number of latent classes, or to different patterns of multimorbidity.

Third, when performing LCA, the 3-class solution was forced. In order to determine whether the latent classes were equivalent, invariance analysis should have been performed [55]. Nevertheless, this solution was forced as we aimed to do comparisons among age subsamples and regions in terms of disease prevalence as well as protective and risk factors.

Finally, the use of multiple imputations could carry some bias. Despite this, the use of multiple imputation procedures is widely advocated when missing data occur in one or more covariates in a regression model and under a MAR assumption [56,57].

For peer review only

Acknowledgments

- The English Longitudinal Study of Ageing (ELSA):

ELSA is supported by the U.S. National Institute of Aging, the National Centre for Social Research, the University College London (UCL), and the Institute for Fiscal Studies. The authors gratefully acknowledge the UK Data Service and UCL who provided data for this paper.

- The Study on Global Ageing and Adult Health (SAGE):

The SAGE study is funded by the U.S. National Institute on Aging and has received financial support through Interagency Agreements (OGHA 04034785; YA1323-08-CN-0020; Y1-AG-1005-01) and Grants (R01-AG034479; IR21-AG034263-0182). The authors gratefully acknowledge the World Health Organization who provided data for this paper.

- The Survey of Health, Ageing and Retirement in Europe (SHARE):

The SHARE study is funded by the European Commission through FP5 (QLK6-CT-2001-00360), FP6 (SHARE-I3: RII-CT-2006-062193, COMPARE: CIT5-CT-2005-028857, SHARELIFE: CIT4-CT-2006-028812) and FP7 (SHARE-PREP: N°211909, SHARE-LEAP: N°227822, SHARE M4: N°261982). Additional funding

For peer review only - http://bmjopen.bmj.com/site/about/guidelines.xhtml

from the German Ministry of Education and Research, the Max Planck Society for the Advancement of Science, the U.S. National Institute on Aging (U01_AG09740-13S2, P01_AG005842, P01_AG08291, P30_AG12815, R21_AG025169, Y1-AG-4553-01, IAG_BSR06-11, OGHA_04-064, HHSN271201300071C) and from various national funding sources is gratefully acknowledged (see www.share-project.org).

Funding: This work was supported by the five-year Ageing Trajectories of Health: Longitudinal Opportunities and Synergies (ATHLOS) project. The ATHLOS project has received funding from the European Union's Horizon 2020 research and innovation program under grant agreement No 635316.

Competing interests: The authors report no conflicts of interest.

Patient consent for publication: not required

Ethics approval: The study protocol was approved by the Committee on the Ethics of Clinical Research, CEIC Fundació Sant Joan de Déu (Protocol No: PIC-22-15). All data were anonymized and EHR confidentially was respected in accordance with national and international law.

Data sharing statement: The original studies data are available on their respective websites: the Study on Global Ageing and Adult Health - SAGE (https://www.who.int/healthinfo/sage/en/), the English Longitudinal Study of Ageing – ELSA (https://www.elsa-project.ac.uk/), and the Survey of Health, Ageing and Retirement in Europe – SHARE (http://www.share-project.org/home0.html). R codes for harmonizing the included variables, as well as the STATA codes for the performed analysis are available on https://github.com/athlosproject/athlos-project.github.io

Additional files: Additional file 1: Table S1Proportion of missingness for indicators of latent classes and variables included in the imputation model.

For peer review only - http://bmjopen.bmj.com/site/about/guidelines.xhtml

Author contributions:

IB: Participated in the database management, drafted the paper, carried out the statistical analyses and worked on the interpretation of data. She also gave final approval of the version to be published and agreed to be accountable for all aspects of the work in ensuring that questions related to the accuracy or integrity of any part of the work are appropriately investigated and resolved; **AS:** Participated in the study design, database management, statistical support and critical revision of the paper. He also gave final approval of the version to be published and agreed to be accountable for all aspects of the work in ensuring that questions related to the accuracy or integrity of any part of the work are appropriately investigated and resolved; **LE:** Participated in the interpretation of data and critical revision of the paper; **HN:** She participated in critical revision of the paper and gave final approval of the version to be published and agreed to be accountable for all aspects of the work in ensuring that questions related to the accuracy or integrity of any part of the work are appropriately investigated and resolved; **MP:** Participated in the study design, and critical revision of the paper. He also gave final approval of the version to be published and agreed to be accountable for all aspects of the work in ensuring that questions related to the accuracy or integrity of any part of the work are appropriately investigated and resolved; **JMH:** Participated in the study design, acquisition of data, interpretation of data and critical revision of the paper. He also gave final approval of the version to be published and agreed to be accountable for all aspects of the work in ensuring that questions related to the accuracy or integrity of any part of the work are appropriately investigated and resolved; **BO:** Participated in the acquisition of data, study design, database management, and critical revision of the paper. She also gave final approval of the version to be published and agreed to be accountable for all aspects of the work in ensuring that questions related to the accuracy or integrity of any part of the work are appropriately investigated and resolved.

For peer review only - http://bmjopen.bmj.com/site/about/guidelines.xhtml

References

1 WHO. Ageing and Health. https://www.who.int/news-room/fact-sheets/detail/ageing-and-health (11 December 2018, date last accessed)

2 Wang H, Naghavi M, Allen C, *et al.* Global, regional, and national life expectancy, all-cause mortality, and cause-specific mortality for 249 causes of death, 1980–2015: a systematic analysis for the Global Burden of Disease Study 2015. *Lancet* 2016;**388**:1459–544. doi:10.1016/S0140-6736(16)31012-1

3 Multimorbidity: a priority for global health research. 2018.

4 Parekh AK, Barton MB. The challenge of multiple comorbidity for the us health care system. *JAMA - J Am Med Assoc* 2010;**303**:1303–4. doi:10.1001/jama.2010.381

5 Mugisha J, Rosenbaum S, Ward PB, *et al.* Chronic physical conditions, multimorbidity and physical activity across 46 low- and middle-income countries. *Int J Behav Nutr Phys Act* 2017;**14**. doi:10.1186/s12966-017-0463-5

6 Chatterji S, Byles J, Cutler D, *et al.* Health, functioning, and disability in older adults - Present status and future implications. *Lancet* 2015;**385**:563–75. doi:10.1016/S0140-6736(14)61462-8

7 Arokiasamy P, Uttamacharya U, Jain K, *et al.* The impact of multimorbidity on adult physical and mental health in low- and middle-income countries: What does the study on global ageing and adult health (SAGE) reveal? *BMC Med* 2015;**13**:1–16. doi:10.1186/s12916-015-0402-8

8 Bao J, Chua KC, Prina M, *et al.* Multimorbidity and care dependence in older adults: A longitudinal analysis of findings from the 10/66 study. *BMC Public Health* 2019;**19**:1–10. doi:10.1186/s12889-019-6961-4

9 Maximova T, Pati S, Biritwum RB, *et al.* The impact of multimorbidity on adult physical and

For peer review only - http://bmjopen.bmj.com/site/about/guidelines.xhtml

mental health in low- and middle-income countries: what does the study on global ageing and adult health (SAGE) reveal? *BMC Med* 2015;**13**:1–16. doi:10.1186/s12916-015-0402-8

10 Afshar S, Roderick PJ, Kowal P, *et al. Applied demography and public health in the 21st century*. Cham, Switzerland: : Springer International Publishing AG 2016. doi:10.1249/mss.0000000000001652

11 Sanchez-Niubo A, Egea-Cortés L, Olaya B et al. Cohort profile: the ageing trajectories of health - longitudinal ppportunities and synergies (ATHLOS) project. *Int J Epidemiol* 2019;**0**:1–11. doi:org/10.1093/ije/dyz077

12 Kowal P, Chatterji S, Naidoo N, *et al.* Data resource profile: The World Health Organization study on global AGEing and adult health (SAGE). *Int J Epidemiol* 2013;**41**:1639–49. doi:10.1093/ije/dys210

13 Steptoe A, Breeze E, Banks J, *et al.* Cohort profile: The English Longitudinal Study of Ageing. *Int J Epidemiol* 2013;**42**:1640–8. doi:10.1093/ije/dys168

14 Börsch-Supan A, Brandt M, Hunkler C, *et al.* Data resource profile: The survey of health, ageing and retirement in europe (SHARE). *Int J Epidemiol* 2013;**42**:992–1001. doi:10.1093/ije/dyt088

15 IDA TWBI. World Bank Open Data. https://data.worldbank.org/ (5 March 2019, date last accessed)

16 Fortier I, Raina P, Van den Heuvel ER, Griffith LE, Craig C, Saliba M, Doiron D, Stolk RP, Knoppers BM, Ferretti V, Granda P BP. Maelstrom Research guidelines for rigorous retrospective data harmonization. 2017;:103–5. doi:10.1093/ije/dyw075

17 Ronald C. Kessler TBÜ. The World Mental Health (WMH) survey initiative version of the World Health Organization (WHO) Composite Diagnostic Interview (CIDI). *Int J Methods Psychiatr*

For peer review only - http://bmjopen.bmj.com/site/about/guidelines.xhtml

For peer review only

Res;**13**:93–121. doi:org/10.1002/mpr.168

18 Radloff L S. The CES-D scale: A self report depression scale for research in the general population. *Appl Psychol Meas* 1977;**1**:385–401. doi:10.1002/ardp.19552881111

19 Reischies F, Lobo A, Turrina C, *et al.* Development of the EURO–D scale – a European Union initiative to compare symptoms of depression in 14 European centres. *Br J Psychiatry* 2008;**174**:330–8. doi:10.1192/bjp.174.4.330

20 Morris JC, Heyman A, Mohs RC, Hughes JP, van Belle G, Fillenbaum G et al. The consortium to establish a registry for Alzheimer's disease (CERAD). Part I. Clinical and neuropsychological assesment of Alzheimer's disease. *Neurology* 1989;**39**:1159–65. doi:10.1212/wnl.39.9.1159

21 WHO. Country groupings. https://www.who.int/quantifying_ehimpacts/global/ebdcountgroup/en/ (7 February 2019, date last accessed)

22 United Nations Statistical Division (UNSD). Countries or areas/geographical regions. https://unstats.un.org/unsd/methodology/m49/ (7 February 2019, date last accessed)

23 Lanza ST, Dziak JJ, Huang L WA, Collins L. *LCA Stata plugin users ' guide (version 1.2.1)*. Penn State: : University Park: The Methodology Center 2018.

24 Tein JY, Coxe S, Cham H. Statistical power to detect the correct number of classes in latent profile analysis. *Struct Equ Model* 2013;**20**:640–57. doi:10.1080/10705511.2013.824781

25 Nylund-Gibson K, Choi AY. Ten frequently asked questions about latent class analysis. *Transl Issues Psychol Sci* 2018;**4**:440–61. doi:10.1037/tps0000176

26 StataCorp. *Stata: Release 13. Statistical Software*. College St. 2013.

27 Rubin DB. *Multiple Imputation for nonresponse in surveys*. United States of America: : John

For peer review only - http://bmjopen.bmj.com/site/about/guidelines.xhtml

Wiley & Sons 1987. doi:10.2307/3172772

28 Garin N, Olaya B, Perales J, *et al.* Multimorbidity patterns in a national representative sample of the Spanish adult population. *PLoS One* 2014;**9**:e84794. doi:10.1371/journal.pone.0084794

29 Olaya B, Moneta MV, Caballero FF, *et al.* Latent class analysis of multimorbidity patterns and associated outcomes in Spanish older adults: A prospective cohort study. *BMC Geriatr* 2017;**17**. doi:10.1186/s12877-017-0586-1

30 Yen L, Valderas JM, McRae IS, *et al.* Multimorbidity and comorbidity of chronic diseases among the senior Australians: prevalence and patterns. *PLoS One* 2014;**9**:e83783. doi:10.1371/journal.pone.0083783

31 Larsen FB, Pedersen MH, Friis K, *et al.* A Latent class analysis of multimorbidity and the relationship to socio-demographic factors and health-related quality of life. A national population-based study of 162,283 Danish Adults. *PLoS One* 2017;**12**:1–17. doi:10.1371/journal.pone.0169426

32 Whitson HE, Johnson KS, Sloane R, *et al.* Identifing patterns of multimorbidity in older americans: application of latent class analysis. *J Am Geriatr Soc* 2016;**64**:1668–73. doi:10.1111/jgs.14201.Identifying

33 Prados-Torres A, Calderón-Larrañaga A, Hancco-Saavedra J, *et al.* Multimorbidity patterns: A systematic review. *J Clin Epidemiol* 2014;**67**:254–66. doi:10.1016/j.jclinepi.2013.09.021

34 Alberti KGM, Zimmet P, Shaw J. Metabolic syndrome -a new world-wide definition. A consensus statement from the International Diabetes Federation. *Abstr Appl Anal* 2006;**23**:469–80. doi:10.1155/2010/307409

35 Margaretten M, Julian L, Katz P, *et al.* Depression in patients with rheumatoid arthritis: description, causes and mechanisms. *Int J Clin Rheumtol* 2011;**6**:617–23.

For peer review only

doi:10.2217/IJR.11.6.Depression

36 Druss BG, Walker ER. Mental disorders and medical comorbidity. 2011.

37 Barbara Messner DB. Smoking and cardiovascular disease: Mechanisms of endothelial dysfunction and early atherogenesis. 2014;**34**:509–15. doi:10.1161/ATVBAHA.113.300156

38 Meade T, Manolios N, Cumming SR, *et al.* Cognitive impairment in rheumatoid arthritis: A systematic review. *Arthritis Care Res* 2018;**70**:39–52. doi:10.1002/acr.23243

39 Lam RW, Kennedy SH, McIntyre RS, *et al.* Cognitive dysfunction in major depressive disorder: effects on psychosocial functioning and implications for treatment. *Can J Psychiatry* 2014;**59**:649–54. doi:10.1177/070674371405901206

40 Dodd JW. Lung disease as a determinant of cognitive decline and dementia. *Alzheimer's Res Ther* 2015;**7**:1–8. doi:10.1186/s13195-015-0116-3

41 Rose Mayeda E, Whitmer RA, Yaffe K. Diabetes and cognition. *Clin Geriatr Med* 2015;**31**:101–ix. doi:10.1016/j.cger.2014.08.021

42 Walker KA, Power MC, Gottesman RF. Defining the relationship between hypertension, cognitive decline, and dementia: a review. 2018;**19**:24. doi:10.1007/s11906-017-0724-3

43 Deckers K, Schievink SHJ, Rodriquez MMF, *et al.* Coronary heart disease and risk for cognitive impairment or dementia: Systematic review and meta-analysis. *PLoS One* 2017;**12**:1–15. doi:10.1371/journal.pone.0184244

44 Leon DA, Shkolnikov VM, McKee M, *et al.* Alcohol increases circulatory disease mortality in Russia: Acute and chronic effects or misattribution of cause? *Int J Epidemiol* 2010;**39**:1279–90. doi:10.1093/ije/dyq102

45 Petrukhin IS, Lunina EY. Cardiovascular disease risk factors and mortality in Russia: Challenges

For peer review only - http://bmjopen.bmj.com/site/about/guidelines.xhtml

and barriers. *Public Health Rev* 2012;**33**:436–49. doi:10.1007/BF03391645

46 Y. He, M. Zhang, E. H. B. Lin et al. Mental disorders among persons with arthritis: results from the World Mental Health Surveys. 2008;**38**:1639–50. doi:10.1017/S0033291707002474

47 Jamrozik E, Musk AW. Respiratory health issues in the Asia-Pacific region: An overview. *Respirology* 2011;**16**:3–12. doi:10.1111/j.1440-1843.2010.01844.x

48 Sandeep Grover, Alakananda Dutt AA. An overview of Indian research in depression. *Indian J Psychiatry* 2010;**52**:178–88. doi:10.4103/0019-5545.69231

49 Gu L, Xie J, Long J, *et al.* Epidemiology of major depressive disorder in Mainland China: A Systematic review. *PLoS One* 2013;**8**:e65356. doi:10.1371/journal.pone.0065356

50 Hou Z, Jiang W, Yin Y, *et al.* The current situation on major depressive disorder in China: Research on mechanisms and clinical practice. *Neurosci Bull* 2016;**32**:389–97. doi:10.1007/s12264-016-0037-6

51 Huntley AL, Johnson R, Purdy S, *et al.* Measures of multimorbidity and morbidity burden for use in primary care and comminuty settings: A systematic review and guide. *Ann Fam Med* 2012;**10**:134–41. doi:10.1370/afm.1363

52 Dal Grande E, Fullerton S, Taylor AW. Reliability of self-reported health risk factors and chronic conditions questions collected using the telephone in South Australia, Australia. *BMC Med Res Methodol* 2012;**12**. doi:10.1186/1471-2288-12-108

53 Ortolá-Devesa JB, Egea-Caparrós JM, Huerta JM, *et al.* Accuracy of self-reported diabetes, hypertension, and hyperlipidemia in the adult spanish population. DINO study findings. *Rev Española Cardiol (English Ed* 2009;**62**:143–52. doi:10.1016/s1885-5857(09)71532-4

54 Martin LM, Leff M, Calonge N, *et al.* Validation of self-reported chronic conditions and health services in a managed care population. *Am J Prev Med* 2000;**18**:215–8. doi:10.1016/S0749-

For peer review only - http://bmjopen.bmj.com/site/about/guidelines.xhtml

For peer review only

3797(99)00158-0

55 Finch H. A comparison of statistics for assessing model invariance in latent class analysis. *Open J Stat* 2015;**5**:191–210. doi:10.4236/ojs.2015.53022

56 White IR, Carlin JB. Bias and efficiency of multiple imputation compared with complete-case analysis for missing covariate values. *Stat Med* 2010;**29**:2920–31. doi:10.1002/sim.3944

57 Sterne JAC, White IR, Carlin JB, *et al.* Multiple imputation for missing data in epidemiological and clinical research: Potential and pitfalls. *BMJ* 2009;**339**:157–60. doi:10.1136/bmj.b2393

For peer review only

Figure 1 Prevalence of diseases in the three latent classes in the total sample and by regions (subsample 50-64 years)

Figure 2 Prevalence of diseases in the three latent classes in the total sample and by regions (subsample +65 years)

For peer review only - http://bmjopen.bmj.com/site/about/guidelines.xhtml

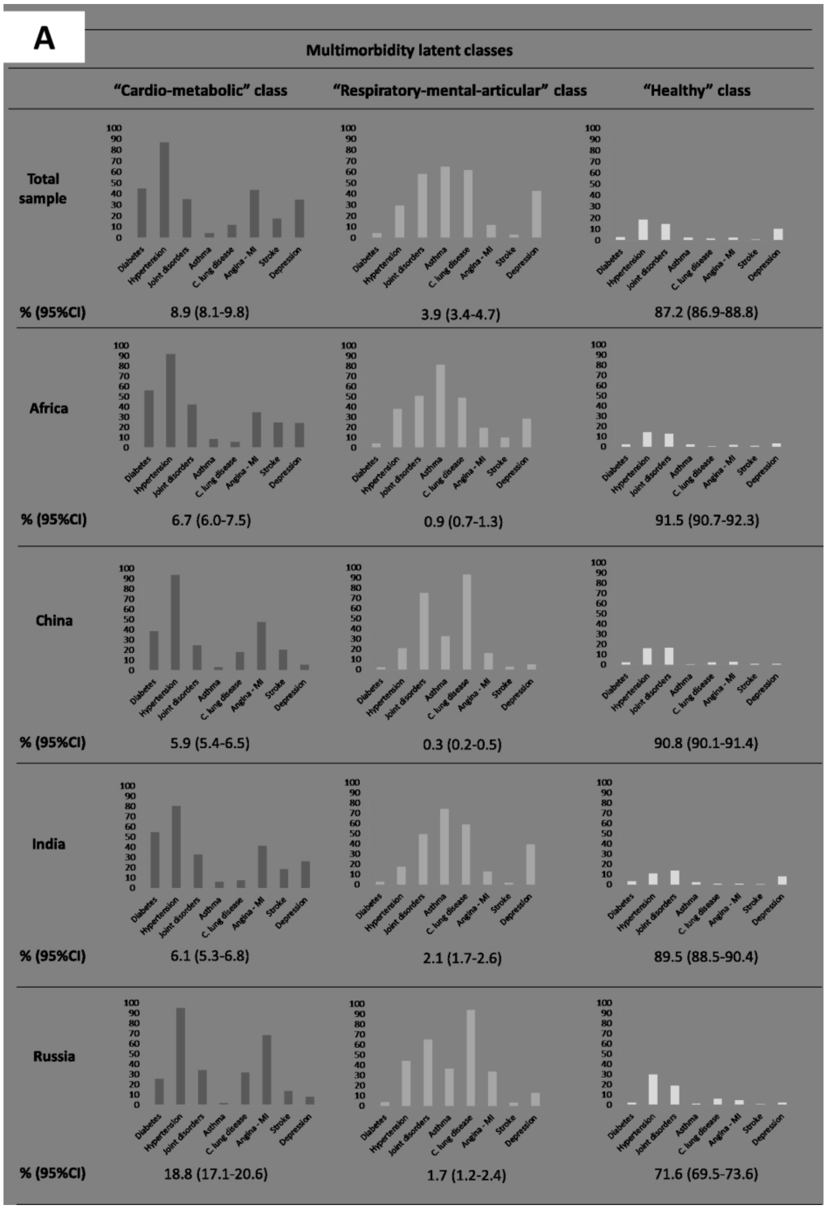

Figure 1 Prevalence of diseases in the three latent classes in the total sample and by regions (subsample 50-64 years). Part a

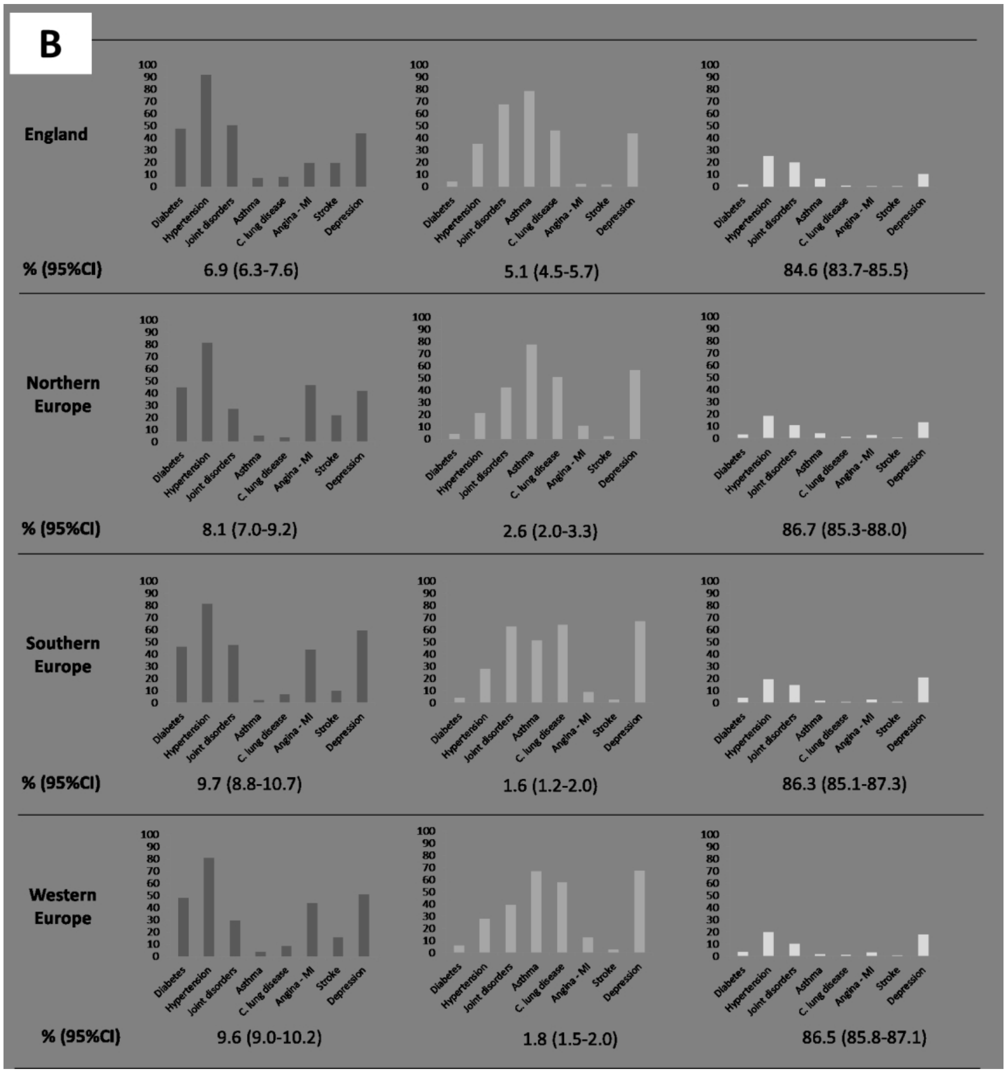

Figure 1 Prevalence of diseases in the three latent classes in the total sample and by regions (subsample 50-64 years). Part b

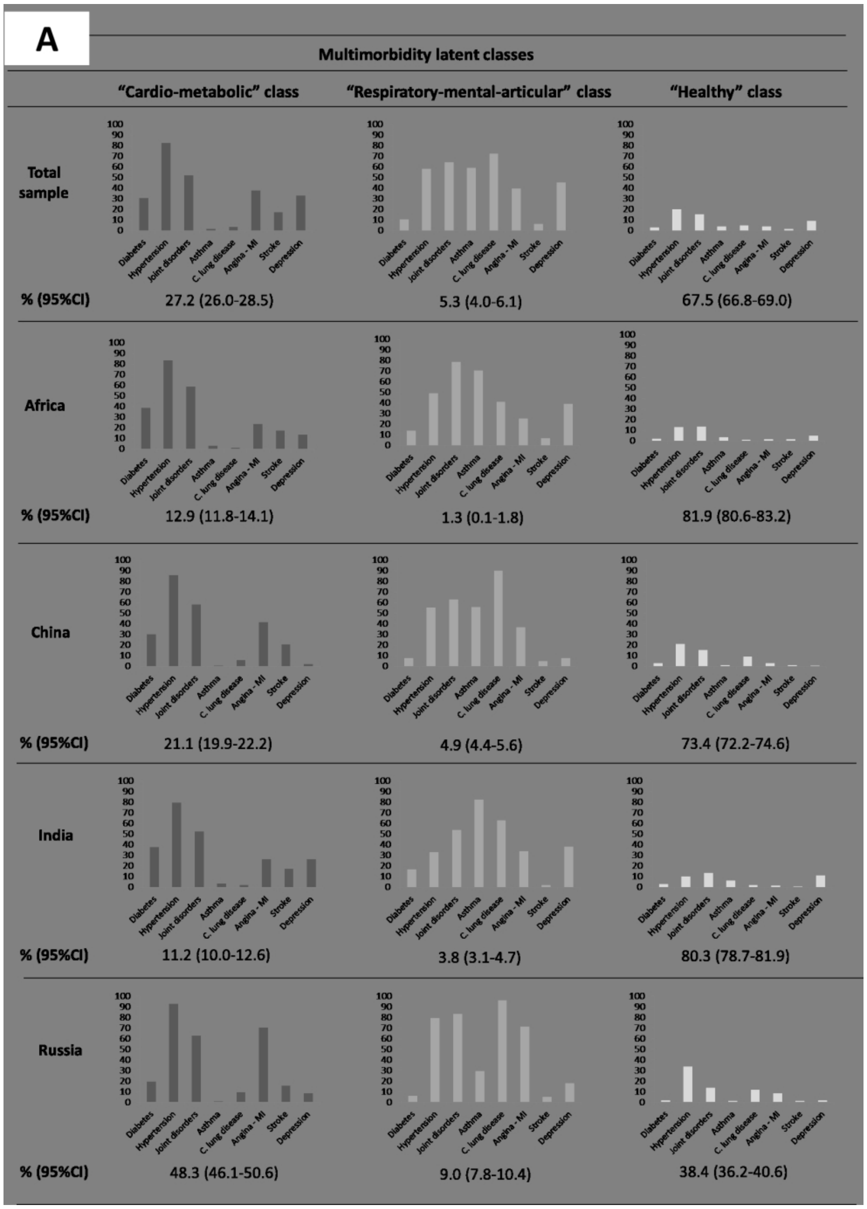

Figure 2 Prevalence of diseases in the three latent classes in the total sample and by regions (subsample +65 years). Part a

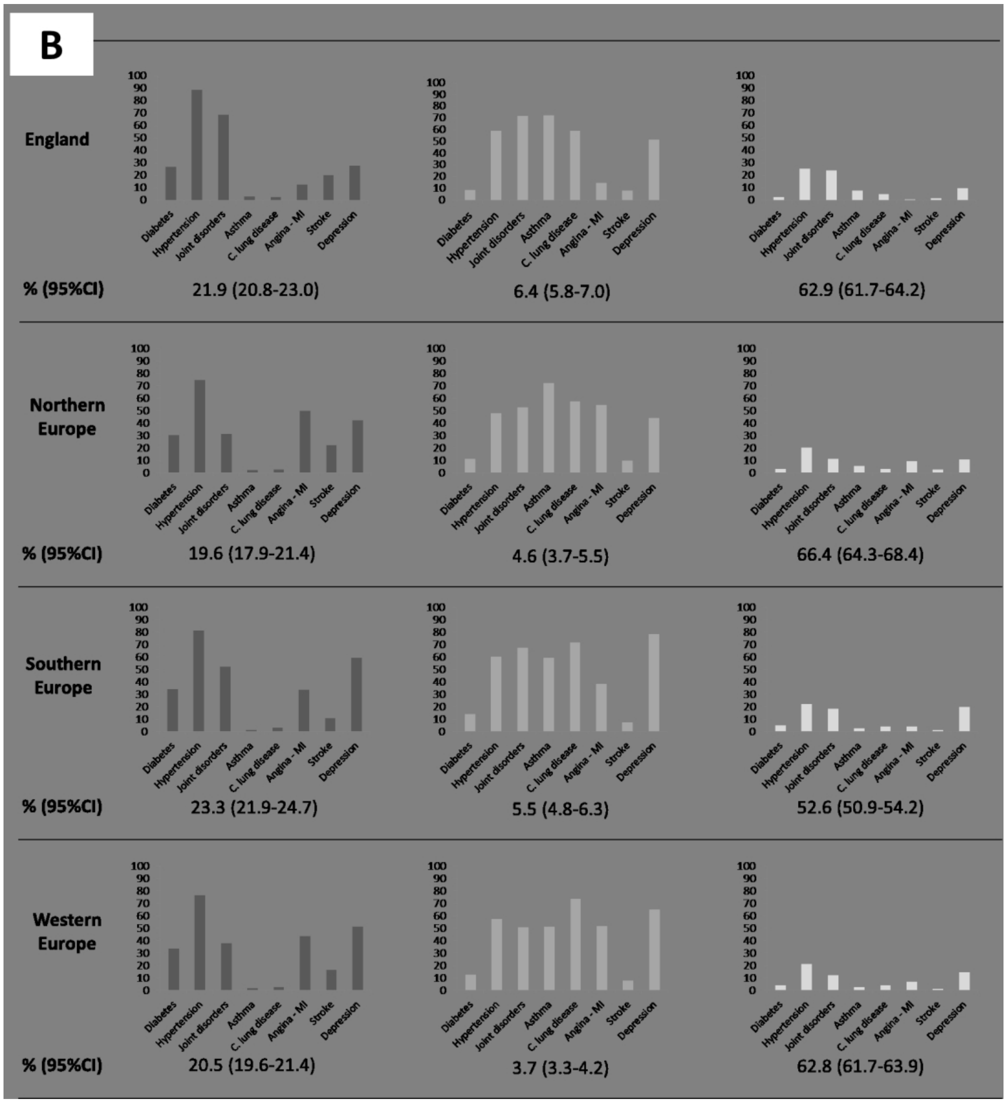

Figure 2 Prevalence of diseases in the three latent classes in the total sample and by regions (subsample +65 years). Part b

Table S1. Proportion of missingness for indicators of latent classes and variables included in the imputation model

(a) Indicators of multimorbidity classes	
	n (%) missingness
Diabetes	62 (0.08)
Hypertension	154 (0.21)
Joint disorders	31 (0.04)
Asthma	82 (0.11)
Chronic lung disease	51 (0.07)
MI - Angina	63 (0.08)
Stroke	41 (0.05)
Depression	1233 (1.70)
(b) Variables used in the regression model	
Sex	0
Age	0
Country	0
Study	0
Marital status	106 (0.14)
Education	11475 (15.91)
Wealth	844 (1.17)
Loneliness	10985 (15.23)
Ever smoked	201 (0.27)
Physical activity	258 (0.35)
Self-rated health	218 (0.30)
ADL – Using the toilet	355 (0.49)
ADL – Bathing or showering	340 (0.47)
ADL – Getting dressed	304 (0.42)
ADL - Eating	400 (0.55)
ADL – Getting in or out of bed	310 (0.42)
ADL – Moving around the house	345 (0.47)
Memory: Immediate recall	1442 (1.99)
Memory: Delayed recall	1448 (2.00)
Verbal fluency	1641 (2.27)

For peer review only

BMJ Open

Multimorbidity patterns in low-middle and high income regions: A multi-region latent class analysis using ATHLOS harmonized cohorts.

Journal:	*BMJ Open*
Manuscript ID	bmjopen-2019-034441.R1
Article Type:	Original research
Date Submitted by the Author:	17-Apr-2020
Complete List of Authors:	Bayes-Marin, Ivet; Parc Sanitari Sant Joan de Déu, Research, Innovation and Teaching Unit; Centro de Investigación Biomédica en Red de Salud Mental, CIBERSAM<br>Sanchez-Niubo, Albert ; Parc Sanitari Sant Joan de Déu, Research, Innovation and Teaching Unit; Centro de Investigación Biomédica en Red de Salud Mental, CIBERSAM<br>Egea-Cortés, Laia; Parc Sanitari Sant Joan de Déu, Research, Innovation and Teaching Unit<br>Nguyen, Hai; King’s College London, Health Service and Population Research Department<br>Prina, A.Matthew; King's College London,<br>Fernández, Daniel; Parc Sanitari Sant Joan de Déu, Research, Innovation and Teaching Unit; CIBERSAM<br>Haro, Josep; Parc Sanitari Sant Joan de Deu, CIBERSAM, University of Barcelona; King Sand University<br>Olaya, Beatriz; Parc Sanitari Sant Joan de Deu, Research and Development Unit
**Primary Subject Heading**:	Epidemiology
Secondary Subject Heading:	Global health
Keywords:	multimorbidity, non-communicable diseases (NCDs), low- and middle-income countries (LMICs), high-income country (HICs), Latent class analysis (LCA)

SCHOLARONE™
Manuscripts

I, the Submitting Author has the right to grant and does grant on behalf of all authors of the Work (as defined in the below author licence), an exclusive licence and/or a non-exclusive licence for contributions from authors who are: i) UK Crown employees; ii) where BMJ has agreed a CC-BY licence shall apply, and/or iii) in accordance with the terms applicable for US Federal Government officers or employees acting as part of their official duties; on a worldwide, perpetual, irrevocable, royalty-free basis to BMJ Publishing Group Ltd ("BMJ") its licensees and where the relevant Journal is co-owned by BMJ to the co-owners of the Journal, to publish the Work in this journal and any other BMJ products and to exploit all rights, as set out in our licence.

The Submitting Author accepts and understands that any supply made under these terms is made by BMJ to the Submitting Author unless you are acting as an employee on behalf of your employer or a postgraduate student of an affiliated institution which is paying any applicable article publishing charge ("APC") for Open Access articles. Where the Submitting Author wishes to make the Work available on an Open Access basis (and intends to pay the relevant APC), the terms of reuse of such Open Access shall be governed by a Creative Commons licence – details of these licences and which Creative Commons licence will apply to this Work are set out in our licence referred to above.

Other than as permitted in any relevant BMJ Author's Self Archiving Policies, I confirm this Work has not been accepted for publication elsewhere, is not being considered for publication elsewhere and does not duplicate material already published. I confirm all authors consent to publication of this Work and authorise the granting of this licence.

For peer review only

Original Research Article

Manuscript title:

Multimorbidity patterns in low-middle and high income regions: A multi-region latent class analysis using ATHLOS harmonized cohorts.

Author's full names and affiliations:

Ivet Bayes-Marin, [a,b] Albert Sanchez-Niubo, [a,b] Laia Egea-Cortés, [a] Hai Nguyen, [c] Matthew Prina, [c] Daniel Fernández, [a,b,d] Josep Maria Haro, [a,b] and Beatriz Olaya [a,b]

From the [a]Research, Innovation and Teaching Unit, Parc Sanitari Sant Joan de Déu, Sant Boi de Llobregat, Spain, [b]Centro de Investigación Biomédica en Red de Salud Mental, CIBERSAM, Madrid, Spain, [c]Health Service and Population Research Department, Institute of Psychiatry, Psychology and Neuroscience, King's College London, London, United Kingdom, [d]Serra Húnter fellow. Department of Statistics and Operations Research, Polytechnic University of Catalonia, Barcelona, Spain.

Corresponding author details:

Correspondence: Albert Sanchez- Niubo. Research, Innovation and Teaching Unit, Parc Sanitari Sant Joan de Déu, Dr Antoni Pujadas 42, Sant Boi de Llobregat, Barcelona 08830, Spain. E-mail: albert.sanchez@pssjd.org

ORCID of the authors:

Ivet Bayes-Marin - 0000-0002-3816-5244

Albert Sanchez-Niubo - 0000-0003-0309-181X

Laia Egea-Cortés - 0000-0003-4002-8254

Hai Nguyen - 0000-0003-2171-1955

For peer review only - http://bmjopen.bmj.com/site/about/guidelines.xhtml

Matthew Prina - 0000-0001-6698-3263

Daniel Fernández - 0000-0003-0012-2094

Josep Maria Haro - 0000-0002-3984-277X

Beatriz Olaya - 0000-0003-2046-3929

Word count (excluding title page, abstract, references, figures and tables): 5081

For peer review only

Abstract

Objectives: Our aim was to determine clusters of non-communicable diseases (NCDs) in a very large, population-based sample of middle-aged and older adults from low- and middle-income (LMICs) and high-income (HICs) regions. Additionally, we explored the associations with several covariates.

Design: The total sample was 72 140 people aged 50+ from three population-based studies (ELSA, SHARE and SAGE) included in the ATHLOS project and representing eight regions with LMICs and HICs. Variables were previously harmonized using an ex-post strategy. Eight NCDs were used in latent class analysis. Multinomial models were made to calculate associations with covariates. All the analyses were stratified by age (50-64 and 65+ years old).

Results: Three clusters were identified: "cardio-metabolic" (8.93% in participants aged 50-64 and 27.22% in those aged 65+), "respiratory-mental-articular" (3.91% and 5.27%) and "healthy" (87.16% and 67.51%). In the younger group, Russia presented the highest prevalence of the "cardio-metabolic" group (18.8%) and England the "respiratory-mental-articular" (5.1%). In the older group, Russia had the highest proportion of both classes (48.3% and 9%). Both the younger and older African participants presented the highest proportion of the "healthy" class. Older age, being woman, widowed, and with low levels of education and income were related to an increased risk of multimorbidity. Physical activity was a protective factor in both age groups and smoking a risk factor for the "respiratory-mental-articular".

Conclusions: Multimorbidity is common worldwide, especially in HICs and Russia. Health policies in each country addressing coordination and support are needed to face the complexity of a pattern of growing multimorbidity.

Keywords: multimorbidity, non-communicable diseases (NCDs), low- and middle- income countries (LMICs), high-income countries (HICs), latent class analysis (LCA).

For peer review only - http://bmjopen.bmj.com/site/about/guidelines.xhtml

Strengths and limitations of this study

- This study used a large, harmonized, multi-regional database, which allowed us to compare two age groups as well as disease prevalence in regions with differing incomes.
- The presence or absence of the non-communicable diseases was based on self-reported measures, and thus might be affected by measurement errors or lack of accuracy.
- Only common diseases across studies were included in the analyses, so this might have led to a smaller number of latent classes, or to different clusters.
- When performing Latent Class Analysis, we forced the solution as we aimed to do comparisons among age subsamples and regions in terms of disease prevalence as well as protective and risk factors.
- The use of multiple imputations for missing data in the covariates could carry some bias.

For peer review only

Background

By 2050, the population aged 60 years and older is expected to reach 2 billion worldwide, compared to 900 million in 2015 [1]. Along with this rapid increase, the incidence of chronic conditions (CC) or non-communicable diseases (NCDs) is also on the rise, having become the leading cause of morbidity and disability worldwide [2].

Multimorbidity, defined as the co-existence of two or more CC, is more common in older adults and is often more prevalent in people of lower socioeconomic status [3]. Multimorbidity is thought to account for 65% of total health care expenses in high-income countries (HICs) because of the huge associated healthcare utilization [4]. Due to the increasing prevalence of multimorbidity, the managing of multiple conditions has become an unavoidable international research priority, because of the high impact on the quality of life of patients and caregivers and on healthcare systems [3].

Most studies on the prevalence of multimorbidity in older people come from HICs, while data from middle-aged adults and low- and middle- income countries (LMICs) are much more limited [5–8]. LMICs are experiencing an increase in life expectancy that, together with changes in lifestyle and environment exposures, are triggering changes in their disease burden profile [3,9]. Few studies have compared patterns of multimorbidity between HICs and LMICs. Afshar et al. [10] used population-based chronic disease data from the World Health Survey to compare multimorbidity prevalence across 27 LMICs and one HIC, and used gross domestic product (GDP) to study inter-country socioeconomic differences. They found high multimorbidity prevalence in all countries, and a positive but non-linear relationship between country GDP and multimorbidity prevalence, suggesting the influence of other factors, such as lifestyles, social conditions, and differences across health systems. Four latent classes were identified in a cross-sectional sample of Australian seniors aged 50 years and over, using self-reported diagnosis of eleven conditions, including cancer and Parkinson's disease [11]. Another study, focusing on complex health care needs of Italian elderly people, found five clusters using 15 diseases [12]. A study conducted in a sample of 162,283 people from a survey of Danish population identified seven latent classes considering 15 chronic diseases and seven age

For peer review only - http://bmjopen.bmj.com/site/about/guidelines.xhtml

groups, ranging from 16 to 104 years [13]. These differences could be explained in light of variations in collection methods, data sources, populations, diseases included, and the analysis performed [11,14,15].

Similarly, the lack of study of differences in multimorbidity between HICs and LMICs may be due to the use of different methodologies, which might hinder comparisons of prevalence and multimorbidity patterns across countries. The integration of data from different studies would allow us to determine differences across regions and cohorts, as well as to explore risk and protective factors involved in the clustering of CC, thereby improving our understanding of the problem and the creation of adapted medical guidelines.

This study aimed to: a) identify multimorbidity clusters in middle-aged (50-64) and older adults (+65) from different regions, classified as LMICs and HICs; b) investigate the associations between multimorbidity clusters and sociodemographic, economic, lifestyles and health status variables; and c) explore differences across regions.

Methods

Study design and data extraction

The present study used data from the Ageing Trajectories of Health: Longitudinal Opportunities and Synergies (ATHLOS) project [16]. Longitudinal data from 17 international cohort studies related to health and ageing were harmonized with the aim of obtaining an integrated dataset and achieving a better understanding of ageing and health processes.

We selected three studies due to their inclusion of the variables of interest and the possibility of comparing HICs and LMICs. Baseline samples of the following studies were included in the analyses: the World Health Organization's Study on Global Ageing and Adult Health (SAGE) [17], the English Longitudinal Study of Ageing (ELSA)[18], and the Survey of Health, Ageing and Retirement in Europe Study (SHARE) [19]. These panel studies included non-institutionalized people aged 50 years and

For peer review only - http://bmjopen.bmj.com/site/about/guidelines.xhtml

older. SAGE comprises six LMICs according to The World Bank Classification [20], namely Ghana, South Africa, Mexico, India, China and Russia; ELSA includes the English population and SHARE covers eleven countries of the European Union and Israel at baseline, considered as HICs [20].

The analyses presented focused on people aged 50 years of age and older who were part of the core sample of each study and who completed a non-proxy interview at baseline. We excluded from the analyses those participants who participated via proxy due to cognitive problems or severe physical limitations. Moreover, people with missing values in sex and age were excluded, resulting in a final sample of 72 140 individuals. Mexico was excluded from the analyses due the high percentage of missingness in the variables of interest (See Additional file 1: Table S1).

Patient and public involvement

No patient involved.

Variables

The following variables were the result of a stringent, ex-post harmonization process using systematic harmonization methodology and tools from Maelstrom Research [21].

Eight NCDs were used to conduct the analysis, including those that were available in the three studies: diabetes, hypertension, asthma, chronic lung disease, joint disorders (arthritis, rheumatism or osteoarthritis), angina or myocardial infarction, stroke, and depression. The presence or absence of these conditions was self-reported and based on a medical diagnosis. Depression was assessed with standardized tools, such as the Composite International Diagnostic Interview (CIDI) in the SAGE study, the Center for Epidemiologic Studies Depression Scale (CES-D) in ELSA, and the EURO-D in SHARE [22–24]. A dichotomous variable (yes/no) was created using the indicated cut-off score for each tool and population based on previous studies [22,25,26].

Self-reported demographic variables included age, sex, level of education (primary or less, secondary, and tertiary), marital status (single, married or currently cohabiting, separated or divorced, and

For peer review only - http://bmjopen.bmj.com/site/about/guidelines.xhtml

widowed) and quintiles of household wealth (first quintile indicating lowest level). Life-styles and health behaviors were 'ever smoked' any type of tobacco and physical activity referring to the practice of vigorous exercise during the last two weeks, both coded as *yes* or *no*. Other health-related variables were self-rated health (good, moderate, or poor), presence or absence of loneliness feelings in the last week, difficulties in activities of daily living (ADL), cognitive performance, and number of diseases.

To assess difficulties in ADL, we used a set of daily self-care activities, which were common across studies, such as problems in using the toilet, bathing or showering, getting dressed, eating, moving, or getting in or out of bed. Each of the ADL difficulties was coded into a *yes/no* if the person answered 'severe' or 'extreme/cannot do it'. To build the set of ADL difficulties, we coded *yes* if the person reported at least one difficulty in any of the six items.

Immediate and delayed recall was assessed using the 10-word learning list task, and verbal fluency utilizing the animal naming test [27]. Continuous total scores were used to perform the analyses. Number of diseases was built by adding up the occurrences of all the above-mentioned NCDs.

Finally, a 7-level regional membership variable was created in order to analyze regional differences, based on the World Health Organization (WHO) and the United Nations Statistical Division (UNSD) regional classification [28,29]. Moreover, the World Bank Classification was used to classify these regions into HICs or LMICs [20]. SAGE includes Africa (Ghana and South Africa), China and India, all of them considered as LMICs. SHARE countries were grouped into three regions: Northern Europe (Denmark, Sweden), Southern Europe (Greece, Italy, and Spain) and Western Europe (Austria, Belgium, France, Germany, Israel, Netherlands, and Switzerland). ELSA and SHARE regions were considered as HICs. Ghana and South Africa were grouped together and named as Africa for practical purposes as well as due to their smaller sample size. These countries are not necessarily representative of the whole continent.

For peer review only - http://bmjopen.bmj.com/site/about/guidelines.xhtml

Statistical analysis

All the analyses were performed using data from the baseline. Descriptive statistics were used to summarize information regarding sociodemographic economic variables and disease prevalence among regions. Confidence intervals (95%IC) were calculated for categorical variables in order to make comparisons across regions.

Latent Class Analysis (LCA) was conducted stratified by age (50-64, +65). Eight NCDs (diabetes, hypertension, asthma, chronic lung disease, joint disorders, angina-myocardial infarction, stroke, and depression) were used as observed indicators without using covariates since we aimed to identify latent classes only based on disease variables. Region was used as cluster when conducting LCA in order to accurately describe disease proportions, indicating that the subjects were not independent random draws, but rather were nested within clusters [30] .

The optimal number of latent classes was determined using the adjusted Bayesian Information Criterion (aBIC), the consistent Akaike Information Criterion (CAIC), and the Entropy Index. Lower values of aBIC and CAIC indicate better fit, whereas entropy index values higher than 0.80 indicate that the latent classes are highly discriminating [31]. The average posterior probability indicates how well a model classifies individuals into their most likely class. Values higher than 0.70 indicate well-identified classes [32]. Additionally, interpretability and clinical judgment were used [32,33].

Missing data in one of the indicators was handled with the full-information maximum likelihood (FIML) technique, assuming missing-at-random (MAR) [34]. Missing data in the covariates was handled using multiple imputation by chained equations (MICE) assuming MAR [34]. The imputation model included the outcome (group membership in one of the latent classes) and all the variables used in the regression models. In the Additional file 1: Table S2-10 there is a report of those variables and the percentage of missingness of each region in the variables of interest.

Multinomial logistic regression models were used to assess the association of each multimorbidity class with several outcomes adjusted for sex, age, marital status, education level, wealth, and region at baseline. Due to potential collinearity between income and education, we checked the significance

For peer review only - http://bmjopen.bmj.com/site/about/guidelines.xhtml

and magnitude of the correlation between both variables. The association was small, and thus both covariates were included as separate variables in the models. Regression models were conducted separately in one hundred imputed datasets and results combined using Rubin's rules [35].

All analyses were conducted with Stata SE version 13.1 (College Station, TX). LCA analyses were performed using a Stata plugin [30].

Results

Descriptive analysis

In Table 1 are presented the main characteristics of the sample by region. The mean age ranged from 62 (SD=9.02) in Southern Asia to 65 years (10.18) in Russia and (10.26) in England. Some 54% were women, 72% were married or cohabitating, and 39% had secondary education. Russia presented the highest number of conditions (mean 1.66) compared to Africa (0.64), China (0.80), and India (0.72).

The most prevalent conditions in the total sample were hypertension (31.2%, 95%CI = 30.9-31.6) and joint disorders (22.4%, 95%CI = 22.0-22.7). Hypertension was particularly high in Russia (56.5%, 95%CI = 54.9-58.1) compared to the other regions. Diabetes prevalence was greater in Southern (11.9%, 95%CI = 11.2-12.7) and Western Europe (10.4%, 95%CI = 9.9-10.9), whereas Africa and China presented the lowest proportions. Similarly, myocardial infarction–angina was highly prevalent in Russia (33.1%, 95%CI = 31.6-34.6), followed by countries of Northern (13.8%, 95%CI = 12.8-14.8), Southern (11.7%, 95%CI = 11.0-12.4) and Western Europe (13.1%, 95%CI =12.6-13.6).

For peer review only

Table 1 Main characteristics of the total sample and by regions

**Region**	Total	Africa[a]	China[b]	India[b]	Russia[b]	England[c]	Northern Europe[d]	Southern Europe[e]	Western Europe[f]
	N=72140	n=7950	n=12840	n=6558	n=3887	n=11 517	n=4573	n=7465	n=17 350
**Age, mean (SD)**	64.05 (9.96)	63.60 (10.26)	63.07 (9.31)	61.80 (9.02)	65.06 (10.18)	65.06 (10.26)	64.78 (10.34)	65.02 (10.17)	64.34 (9.96)
**Female, % (95% CI)**	54.0 (53.6-54.4)	52.3 (51.2-53.4)	52.9 (52.1-54.8)	49.6 (48.4-50.8)	64.6 (63.0-66.1)	54.6 (53.7-55.5)	53.2 (51.8-54.7)	55.4 (54.2-56.5)	54.1 (53.4-54.9)
**Marital status, % (95% CI)**									
Single	4.1 (4.0-4.3)	6.7 (6.2-7.3)	0.9 (0.7-1.6)	1.0 (0.8-1.2)	2.8 (2.3-3.4)	5.0 (4.6-5.4)	5.5 (4.8-6.2)	6.3 (5.8-6.9)	4.9 (4.6-5.3)
Married	71.5 (71.1-72.0)	54.5 (53.4-55.6)	83.5 (82.8-84.1)	74.1 (73.1-75.2)	56.1 (54.5-57.7)	69.1 (68.2-69.9)	72.4 (71.0-73.7)	73.3 (72.3-74.3)	73.3 (72.7-74.0)
Divorced	5.9 (5.7-6.2)	10.4 (9.7-11.1)	1.7 (1.5-2.0)	0.6 (0.5-0.9)	8.3 (7.4-9.2)	9.0 (8.5-9.5)	9.6 (8.8-10.5)	2.6 (2.3-3.0)	6.8 (6.5-7.2)
Widowed	18.4 (18.1-18.6)	27.3 (26.3-28.3)	13.9 (13.3-14.6)	24.3 (23.2-25.3)	32.7 (31.2-34.2)	16.9 (16.3-17.6)	12.5 (11.6-13.5)	17.8 (16.9-18.6)	14.9 (14.4-15.4)
**Education level % (95% CI)**									
Primary or less	33.4 (33.0-33.7)	30.5 (29.5-31.5)	37.9 (37.1-38.8)	25.5 (24.5-26.6)	9.9 (9.0-10.9)	42.4 (41.5-43.4)	29.6 (28.3-30.9)	59.4 (58.3-60.5)	23.2 (22.6-23.8)
Secondary	38.7 (38.4-39.1)	19.0 (18.2-19.9)	33.2 (32.4-34.0)	18.2 (17.3-19.2)	69.3 (67.9-70.8)	37.5 (36.7-38.4)	45.1 (43.6-46.5)	31.3 (30.2-32.3)	55.1 (54.3-55.8)
Tertiary	12.0 (11.9-12.2)	3.9 (3.5-4.3)	4.7 (4.3-5.0)	5.0 (4.4-5.5)	19.7 (18.5-21.0)	11.1 (10.6-11.7)	24.2 (23.0-25.5)	9.1 (8.4-9.7)	20.8 (20.2-21.4)
**Wealth (quintiles% (95% CI)**									
1st (worse)	19.3 (19.1-19.6)	19.3 (18.4-20.2)	19.8 (19.1-20.5)	16.2 (15.3-17.1)	18.2 (17.0-19.5)	19.0 (18.3-19.7)	20.7 (19.6-21.9)	20.3 (19.4-21.2)	19.9 (19.3-20.5)
2nd	19.7 (19.4-20.0)	19.7 (18.4-20.2)	19.7 (19.0-20.4)	18.6 (17.6-19.5)	19.8 (18.5-21.1)	19.3 (18.6-20.1)	20.4 (19.3-21.6)	20.4 (19.5-21.3)	19.9 (19.4-20.6)
3rd	19.7 (19.4-19.9)	19.8 (18.9-20.7)	20.1 (19.4-20.8)	18.4 (17.5-19.4)	20.3 (19.0-21.6)	19.7 (18.9-20.4)	20.1 (19.0-21.3)	19.8 (18.9-20.7)	19.5 (18.9-20.1)
4th	19.9 (19.7-20.3)	20.5 (19.6-21.4)	20.5 (19.8-21.2)	21.5 (20.5-22.5)	20.0 (18.8-21.3)	19.6 (18.9-20.3)	19.7 (18.5-20.8)	19.4 (18.6-20.4)	19.3 (18.8-19.9)
5th (best)	20.1 (19.8-20.4)	20.4 (19.5-21.3)	19.7 (19.0-20.4)	24.8 (23.8-25.9)	21.6 (20.3-22.9)	19.6 (18.9-20.3)	18.9 (17.8-20.1)	18.9 (18.0-19.8)	19.4 (18.8-20.0)
**Nº diseases, *mean (SD)***	1.02 (1.14)	0.64 (0.94)	0.80 (0.99)	0.72 (0.97)	1.66 (1.38)	1.19 (1.13)	1.02 (1.10)	1.28 (1.25)	1.10 (1.16)
**Diseases, % (95% CI)**									
Diabetes	8.5 (8.3-8.7)	6.6 (6.1-7.2)	6.5 (6.1-7.0)	7.3 (6.7-7.9)	9.0 (8.1-10.0)	7.4 (6.9-7.9)	8.2 (7.4-9.0)	11.9 (11.2-12.7)	10.4 (9.9-10.9)
Hypertension	31.2 (30.9-31.6)	21.5 (20.6-22.4)	27.4 (26.6-28.2)	17.5 (16.6-18.5)	56.5 (54.9-58.1)	37.8 (36.9-38.7)	29.3 (28.0-30.7)	35.6 (34.5-36.7)	32.3 (31.6-33.0)
Joint disorders	22.4 (22.0-22.7)	17.8 (16.9-18.6)	22.1 (21.4-22.8)	17.9 (17.0-18.9)	35.2 (33.7-36.7)	32.5 (31.6-33.3)	15.7 (14.6-16.8)	26.3 (25.3-27.3)	16.8 (16.3-17.4)
Asthma	5.5 (5.3-5.6)	4.1 (3.7-4.5)	2.4 (2.2-2.7)	6.9 (6.3-7.6)	3.4 (2.9-4.0)	11.7 (11.1-12.3)	7.6 (6.8-8.4)	4.1 (3.7-4.6)	4.1 (3.8-4.4)
Chronic lung disease	6.1 (5.9-6.3)	1.5 (1.2-1.7)	8.6 (8.1-9.1)	4.1 (3.6-4.6)	17.9 (16.8-19.2)	6.5 (6.1-7.0)	4.5 (3.9-5.2)	5.4 (4.9-6.0)	4.9 (4.6-5.3)
MI - Angina	10.0 (9.8-10.3)	4.5 (4.1-5.0)	8.8 (8.3-9.3)	4.9 (4.4-5.5)	33.1 (31.6-34.6)	3.3 (3.0-3.6)	13.8 (12.8-14.8)	11.7 (11.0-12.4)	13.1 (12.6-13.6)
Stroke	3.8 (3.7-3.9)	3.2 (2.8-3.6)	3.5 (3.2-3.8)	2.2 (1.9-2.6)	6.0 (5.3-6.8)	4.5 (4.1-4.9)	5.0 (4.4-5.7)	3.1 (2.8-3.6)	3.9 (3.7-4.2)
Depression	15.3 (15.0-15.5)	5.8 (5.3-6.3)	1.2 (1.1-1.4)	12.1 (11.3-12.9)	5.2 (4.5-5.9)	16.5 (15.8-17.2)	18.8 (17.7-20.0)	31.7 (30.6-32.7)	25.0 (24.3-25.6)

Note. [a]SAGE study - Africa: Ghana, South Africa; [b]SAGE study; [c]ELSA study- England; [d]SHARE study - Northern Europe: Denmark, Sweden; [e]SHARE study - Southern Europe: Greece, Italy, Spain; [f]SHARE study - Western Europe: Austria, Belgium, France, Germany, Israel, Netherlands, Switzerland.

The analyses were performed before multiple imputation procedure

For peer review only - http://bmjopen.bmj.com/site/about/guidelines.xhtml

Joint disorders were more prevalent in Russia (35.2%, 95%CI = 33.7-36.7) and England (32.5%, 95%CI =31.6-33.3). The prevalence of asthma was greater in England than other regions (11.7%, 95%CI = 11.1-12.3) and chronic lung disease was greater in Russia (17.9%, 95%CI = 16.8-19.2).

As for the prevalence of depression, European countries presented the highest values, especially in Southern (31.7%, 95%CI = 30.6-32.7) and Western Europe (25.0%, 95%CI = 24.3-25.6), whereas LMICs showed very low proportions, especially in China, where only 1.2% of people aged 50+ presented depression.

Multimorbidity patterns

Table 2 displays the aBIC, CAIC, and entropy values, proportions, and average posterior probability of each latent class, for a two- to five-class model in both age subsamples. In the younger subsample (50-64), the five-class solution yielded the lowest aBIC and CAIC values and the highest entropy value (0.67). However, it was dismissed because one of the latent classes was very infrequent and the posterior probabilities were far below 0.70. Similarly, the four-class model was rejected for an inadequate posterior probability value in one of the classes (0.52). The model finally selected was the three-class model. The three-class solution was also chosen for the older age group because of lower posterior probability values in the four- and five-class models in spite of lower aBIC and CAIC values.

We named each latent class according to the most prevalent diseases within each latent class. Figure 1 shows the distribution of each condition across the three latent classes ("cardio-metabolic", "respiratory-mental-articular", and "healthy" class) in the total sample and by regions. The "cardio-metabolic" class presented excess prevalence of diabetes, hypertension, myocardial infarction or angina, and stroke, comprising 8.93% of the total sample in the younger group and 27.22% in the older group. The "respiratory-mental-articular" class, which comprised 3.91% and 5.27% of each sample, respectively, showed greater prevalence of joint disorders, asthma, chronic lung diseases,

For peer review only - http://bmjopen.bmj.com/site/about/guidelines.xhtml

and depression. Finally, the “healthy” class presented low prevalence of conditions, comprising 87.16% of the sample in the first age group and 67.51% in the second group.

For peer review only

For peer review only - http://bmjopen.bmj.com/site/about/guidelines.xhtml

Table 2 Comparison between models in individuals aged 50-64 and +65

	Aged 50-64					Aged ≥65				
No. of latent classes	Information Criteria Indices		Classification Quality	Latent classes, n (%)	Average posterior probability	Information Criteria Indices		Classification Quality	Latent classes, n (%)	Average posterior probability
	aBIC	CAIC	Entropy			aBIC	CAIC	Entropy		
2	1512.91	1583.94	0.51	33023 (82.15)<br>7177 (17.85)	.88<br>.75	1603.30	1674.33	0.39	21113 (66.10)<br>10827 (33.90)	.83<br>.75
**3**	**875.23**	**983.86**	**0.63**	**3589 (8.93)**<br>**1571 (3.91)**<br>**35040 (87.16)**	**.76**<br>**.67**<br>**.87**	**1032.83**	**1141.46**	**0.50**	**8693 (27.22)**<br>**1684 (5.27)**<br>**21563 (67.51)**	**.71**<br>**.68**<br>**.81**
4	777.14	923.37	0.43	25701 (63.93)<br>626 (1.56)<br>7754 (19.29)<br>6119 (15.22)	.52<br>.72<br>.80<br>.68	817.60	963.83	0.63	9557 (29.92)<br>1474 (4.61)<br>17220 (53.91)<br>3689 (11.55)	.65<br>.78<br>.86<br>.77
5	661.04	844.87	0.67	4578 (11.39)<br>247 (0.61)<br>32423 (80.65)<br>1359 (3.38)<br>1593 (3.96)	.64<br>.67<br>.85<br>.48<br>.64	689.22	873.05	0.59	11094 (34.73)<br>1094 (3.43)<br>14155 (44.32)<br>1148 (3.59)<br>4449 (13.93)	.77<br>.71<br>.76<br>.63<br>.72

Note: Boldface indicates the final selected model. *aBIC* adjusted Bayesian Information Criterion, *CAIC* Consistent Akaike Information Criterion

For peer review only

For peer review only - http://bmjopen.bmj.com/site/about/guidelines.xhtml

Differences in the proportions of multimorbidity classes were found across regions (Figure 1). The "cardio-metabolic" class (18.8%, 95%CI =17.1-20.6) was significantly greater in Russia than in other regions, and England (5.1%, 95%CI =4.5-5.7) showed a higher proportion of individuals classified into the "respiratory-mental-articular" class. The "healthy" class was higher in Africa (91.5%, 95%CI = 90.7-92.3), China (90.8%, 95%CI = 90.1-91.4), and India (89.5%, 95%CI = 88.5-90.4), and remarkably lower in Russia (71.6%, 95%CI = 69.5-73.6) compared to other regions.

----- FIGURE 1 HERE -----

Similar results were found for the older group (Figure 2). In Russia, the "cardio-metabolic" class was significantly higher than in other regions (48.3%, 95%CI = 46.1-50.6) whereas the "healthy" class was the least frequent class compared with the rest of regions (38.4%, 95%CI = 36.2-40.6), followed by Southern Europe (52.6%, 95%CI = 50.9-54.2). Africa and India showed lower proportions of individuals classified into the "cardio-metabolic" class (12.9%, 95%CI = 11.8-14.1 and 11.2%, 95%CI = 10.0-12.6, respectively).

----- FIGURE 2 HERE -----

Association between multimorbidity classes and covariates

In the Additional file 2: Table S1 are presented the unadjusted relative risk ratios (RRR) for both age subsamples. The "healthy" class was used as the reference group. In the case of the younger subsample, and compared with the "healthy" class, individuals classified into the "cardio-metabolic" and "respiratory-mental-articular" classes were more likely to be older (RRR = 1.09, 95%CI = 1.08-1.10; RRR = 1.06, 95%CI = 1.04-1.07, respectively) and being widowed (RRR = 1.4, 95%CI = 1.1-1.7) and divorced in the "respiratory-mental-articular" class (RRR = 1.7, 95%CI = 1.2-2.4). Being a man, having tertiary education, and high levels of wealth had a protective effect for being in both multimorbidity groups, compared with the "healthy" class. Similarly, those individuals from the older

For peer review only - http://bmjopen.bmj.com/site/about/guidelines.xhtml

subsample who were in the fourth and fifth quintile (RRR = 0.8, 95%CI = 0.7-1.0) were less likely to be classified into the "respiratory-mental-articular" class, compared with the "healthy" group.

Regarding the association of regions and multimorbidity groups, some differences were found in the younger subsample. Taking Africa as the reference category, participants from Russia were more likely to be classified into the "cardio-metabolic" class (RRR = 3.6, 95%CI = 3.0-4.2), whereas individuals from England (RRR = 5.6, 95%CI = 4.1-7.7), Northern Europe (RRR = 2.8, 95%CI = 1.9-4.1) and India (RRR = 2.2, 95%CI = 1.5-3.2) showed higher risk of being in the "respiratory-mental-articular" class. In the case of the older subsample, all regions had greater risk of being classified into the "respiratory-mental-articular" class, compared to the "healthy" class, especially participants from Russia (RRR = 14.5, 95%CI = 10.3-20.3), compared to Africa.

Table 3 shows the adjusted relative risk ratios (RRR) for both age subsamples, taking the "healthy" class as the reference group. Both multimorbidity classes (cardio-metabolic and respiratory-mental-articular) were associated with all the covariates in the younger group, except for smoking status in the "cardio-metabolic" class.

In the younger individuals subsample, both latent classes were more likely to be associated with the presence of feelings of loneliness (RRR = 1.8, 95%CI = 1.7-2.0; RRR = 2.5, 95%CI = 2.0-3.0), limitations in ADL (RRR = 3.2, 95%CI = 2.9-3.6; RRR = 3.9, 95%CI = 3.3-4.7) and worse health status (RRR = 12.8, 95%CI = 11.3-14.4; RRR = 12.9, 95%CI = 10.5-16.0). Physical activity had a protective effect for being in these classes and having smoked was a risk factor only for being classified into the "respiratory-mental-articular" class (RRR = 1.5, 95%CI = 1.2-1.7). Conversely, those older individuals who had ever smoked had a higher risk of being in the "respiratory-mental-articular" group (RRR = 1.8, 95%CI = 1.5-2.0) and a lower risk of being classified into the "cardio-metabolic" class (RRR = 1.0, 95%CI = 0.9-1.0). For both age subsamples, better performance in verbal memory was significantly associated with less

For peer review only - http://bmjopen.bmj.com/site/about/guidelines.xhtml

risk of being classified into the two multimorbidity classes. Similarly, higher scores in verbal fluency were a protective factor for multimorbidity, compared with the healthy individuals group.

Table 3 Association between latent multimorbidity membership and outcomes in individuals aged 50-64 and ≥65

Outcomes[a]	Aged 50-64		Aged ≥65	
	"Cardio-metabolic" class	"Respiratory-mental-articular" class	"Cardio-metabolic" class	"Respiratory-mental-articular" class
Loneliness (yes/no)	1.8 (1.7-2.0)	2.5 (2.0-3.0)	1.3 (1.2-1.5)	1.9 (1.7-2.3)
Ever smoked (yes/no)	1.1 (1.0-1.2)	1.5 (1.2-1.7)	1.0 (0.9-1.0)	1.8 (1.5-2.0)
Physical activity (yes/no)	0.4 (0.4-0.5)	0.5 (0.5-0.6)	0.5 (0.5-0.6)	0.4 (0.4-0.5)
Limitations in ADL (yes/no)	3.2 (2.9-3.6)	3.9 (3.3-4.7)	2.3 (2.1-2.5)	4.0 (3.5-4.6)
Self-rated health				
Good	1.0	1.0	1.0	1.0
Moderate	4.8 (4.3-5.2)	3.9 (3.2-4.7)	3.1 (2.9-3.4)	5.7 (4.8-6.8)
Poor	12.8 (11.3-14.4)	12.9 (10.5-16.0)	6.2 (5.6-6.9)	19.4 (16.2-23.4)
Memory: Immediate recall	0.9 (0.9-0.9)	0.9 (0.9-1.0)	0.9 (0.9-0.9)	0.9 (0.9-1.0)
Memory: Delayed recall	0.9 (0.9-0.9)	0.9 (0.9-1.0)	0.9 (0.9-0.9)	0.9 (0.9-1.0)
Verbal fluency	1.0 (1.0-1.0)	1.0 (1.0-1.0)	0.9 (0.9-1.0)	1.0 (1.0-1.0)

Note: The reference group for the multimorbidity group variable was the "Healthy" class
Relative Risk Ratios (95% confidence interval) from multinomial logistic regression models
Models were run in 100 imputed datasets and results combined using Rubin's rules
ADL Activities of Daily Living
[a]Adjusted for sex, age, marital status, education level, wealth and region

Discussion

To the best of our knowledge, this is the first multi-region study to use harmonized data to compare multimorbidity patterns across different regions from three distinct population-based cohorts. We identified three latent classes of multimorbidity based on the presence of eight NCDs: the "cardio-metabolic", the "respiratory-mental-articular" and the "healthy" class. The same clusters were identified in another study using SAGE original data, applying exploratory factor analysis (EFA) in a sample of 41 909 individuals aged 50 years or older [36]. Similarly, a study of a representative sample of Spanish community-dwelling adults over 50 years old also found three latent classes using eleven chronic conditions, showing similar diseases distributions among the multimorbidity clusters [37].

For peer review only - http://bmjopen.bmj.com/site/about/guidelines.xhtml

In our study, for both age groups the majority of the sample was classified into the "healthy" class, 87.16% and 67.51%, respectively. This latent group has previously been described in studies which applied LCA [11,13,37,38]. Likewise, the other two identified classes are similar to those reported in a systematic review based on 14 studies of multimorbidity patterns [39]. In this review, the most prevalent diseases in the "cardio-metabolic" group were diabetes, hypertension, heart diseases, hyperlipidemia, and obesity; and in the second group conditions such as mental disorders, thyroid disease, neurological disease, pain, asthma or chronic lung diseases, musculoskeletal disorders, obesity, and gastroesophageal reflux disease were included. Despite the fact that we included a smaller number of diseases, we found analogous patterns. In our study, 8.93% of the younger group (50-64) and 27.22% of the older were classified into the "cardio-metabolic" class, including individuals with higher prevalence of diabetes, hypertension, myocardial infarction or angina, and stroke. This clustering of diseases is similar to the metabolic syndrome, which has metabolically-related cardiovascular risk factors and greater risk of stroke and diabetes [40]. Lastly, the least prevalent group was the "respiratory-mental-articular" class, consisting of greater prevalence of joint disorders, asthma, chronic lung diseases, and depression. Association between depression and arthritis has commonly been reported, with socioeconomic and disease factors reported as being involved in its association, as well as systemic inflammation mechanisms [3]. Nevertheless, the links between depression and chronic lung diseases, and chronic lung diseases and arthritis, despite having been studied, remain unclear [40,41].

Analogous latent multimorbidity classes have been found among both age groups. Despite this, certain aspects should be pointed out. As expected, the proportion of participants classified into the "healthy" class was greater in participants aged 50-64 (87.16%) compared to those aged +65 years old (67.51%), illustrating higher multimorbidity in elderly individuals. The distribution of CC was also less clear in the older subsample. For example, both joint disorders and angina–myocardial infarction were similarly present in the "cardio-metabolic" and "respiratory-mental-articular" categories,

For peer review only - http://bmjopen.bmj.com/site/about/guidelines.xhtml

whereas in the younger participants (50-64) subsample we observed a more differentiated profile of those chronic conditions that cluster into one latent class. For example, respiratory-related diseases (asthma, chronic lung diseases) are highly presented in the "respiratory-mental-articular" class, while very infrequent among middle-aged people classified into the "cardio-metabolic" group. It is worth mentioning that although depression is frequently observed among participants classified into the "respiratory-mental-articular" class, it is not infrequent among people within the "cardio-vascular" class. This may be due to the relationship between mental and physical disorders, which has frequently been reported, suggesting a bidirectional association between them [42]. On the one hand, medical conditions could be accompanied by a high symptom burden, leading to depression, and, on the other, depression could be a risk factor for medical conditions, since depressive symptoms could increase the incidence of behaviors, such as smoking, alcohol intake, poor diet, or physical inactivity, which are risk factors for NCDs [3,42].

One important implication of our findings is the relatively high proportion of people aged 50-64 with multimorbidity. Thus, preventive and intervention programs are also needed for this population to mitigate the multimorbidity burden.

Our results show that these multimorbidity patterns are qualitatively different, but only if compared to the "healthy" class in terms of sociodemographic and economic characteristics, lifestyles, and health status variables. As has been reported in the literature, being older, woman, widowed, with a lower level of education and lower socioeconomic status are related to an increased risk of multimorbidity [3]. In addition, those individuals with multiple chronic conditions were more likely to have limitations in ADL, especially those classified into the "respiratory-mental-articular" group, similar to what was found in another study of multimorbidity [37]. Physical activity seems to be a protective factor for being classified into the "respiratory-mental-articular" class, whereas smokers were more likely to be classified into the "respiratory-mental-articular" class, but not the "cardio-metabolic" class. This is inconsistent with the literature, since cigarette smoking is considered a

For peer review only

For peer review only - http://bmjopen.bmj.com/site/about/guidelines.xhtml

major cause of cardiovascular diseases (CVDs). However, smoking is probably the most complex and least understood risk factor for CVDs [43].

One interesting finding is the association between cognition outcomes and multimorbidity in both age subsamples. Better performance in verbal memory and fluency was related to less risk of being classified into the multimorbidity groups, with similar results among latent classes. Impaired cognition has been associated with conditions such as arthritis [44], depression [45], and respiratory diseases [46], cardiovascular conditions, diabetes [47], hypertension [48], and coronary heart diseases [49].

Concerning the regional distribution of multimorbidity, Russia accounted for the highest burden as opposed to Africa, China, and India. The "cardio-metabolic" class is especially common in this country, with a prevalence of 18.82% in the younger and 48.34% in the older subsample. Prevalence of CVDs, such as hypertension, myocardial infarction or angina, and stroke, was also higher in Russia. This high proportion could be related to the high rate of alcohol consumption and rapid societal changes experienced in this country which might account for increased risk of circulatory diseases [50,51]. Followed by Russia, European regions showed higher rates of multimorbidity. NCDs such as hypertension, joint disorders, respiratory diseases, and depression were highly prevalent, especially in England and Southern Europe, where the "respiratory-mental-articular" class was highly prevalent in both age subsamples. The relationship between mood disorders such as depression and joint disorders has been previously reported in other studies, though the underlying cause remains unclear [36,37,39]. Notwithstanding, previous studies suggested that the emotional burden of joint disorders may contribute to the onset of psychiatric disorders [36,52].

LMICs such as Africa, China, and India showed lower rates of multimorbidity compared to Russia and other HICs. However, there was a wide variation in terms of some diseases, such as respiratory diseases and depression. Asthma and chronic lung diseases were highly prevalent in India and China,

For peer review only - http://bmjopen.bmj.com/site/about/guidelines.xhtml

influenced by factors such as increasing smoking rates, air pollution, and occupational lung diseases in these countries [53]. As reported in previous studies [54], depression was remarkably prevalent in India, whereas the lowest prevalence was observed in China. This is in line with previous epidemiological studies on the prevalence of depression in Chinese older people, suggesting differences in diagnostic criteria that make depression less diagnosed; somatic symptoms are more prevalent in this population instead of sadness, and lack of interest and energy. Moreover, stigma and prejudice in Chinese population might also contribute to under-reporting depressive symptoms [55,56]. Furthermore, the variation found across regions in terms of depression prevalence could be due to cultural differences in expressions or expectations of mood disorders or mental health[57].

The highest burden of multimorbidity in HICs could be explained by an increased level of development in the HICs. Notwithstanding, LMICs are experiencing a change in lifestyle and environmental exposures which contributes, as in HICs, to multimorbidity. Thus, the increased burden of NCDs, in addition to the existing burden of infectious diseases such as HIV/AIDS, worsens multimorbidity management [3] . Moreover, the differences found in the regional distribution of multimorbidity might be linked to different stages of development of their health systems, since there are differences between LMICs and HICs in terms of opportunities and barriers to improving the organization, integration, and delivery of multimorbidity care [3].

Strengths and limitations

A major strength of this study is the use of a large, harmonized, multi-regional database. Research on multimorbidity has typically been hampered by several factors, such as the exclusion of patients with multimorbidity from participation, targeting of research mostly on elderly individuals, and a shortage of studies focusing on LMICs. The ATHLOS study allowed us to compare two age groups (50-64 and 65 or older) as well as disease prevalence and clusters of conditions in regions with differing incomes in a very large, diverse population-based study of middle-aged and older adults.

For peer review only - http://bmjopen.bmj.com/site/about/guidelines.xhtml

Some limitations should be considered when interpreting our findings. First, the presence or absence of the NCDs was based on self-reported measures, and thus might be affected by measurement errors or lack of accuracy. Nevertheless, some authors sustain self-reported diagnostics as a well-established method for the measurement of multimorbidity in population-based studies [58–61]. Second, we could only focus on those diseases that were common across studies. Conditions such as obesity, cancer, kidney disease, and neurological illness were not evaluated. This might have led to a smaller number of latent classes, or to different patterns of multimorbidity.

Third, when performing LCA, the three-class solution was forced. In order to determine whether the latent classes were equivalent, invariance analysis should have been performed [62]. Nevertheless, this solution was forced as we aimed to do comparisons among age subsamples and regions in terms of disease prevalence as well as protective and risk factors.

Finally, the use of multiple imputations could carry some bias. Despite this, the use of multiple imputation procedures is widely advocated when missing data occur in one or more covariates in a regression model and under a MAR assumption [63,64].

The results of this study suggest that NCDs cluster together in non-random associations across several regions worldwide. The three qualitatively distinct entities are also linked to several sociodemographic and economic characteristics, lifestyles, and health status variables. A deeper understanding of the interactions across regions and the studied variables is needed. Knowledge regarding broad patterns of conditions may contribute to the creation and implementation of guidelines that consider clusters of conditions instead of single diseases, since multimorbidity has become an unavoidable reality. Future efforts should focus on the underlying mechanisms of these clusters as well as their stability over time using longitudinal data. Moreover, cohort and age effects should be explored as might influence the likelihood of reporting some diagnosis and hence result in different multimorbidity patterns.

For peer review only - http://bmjopen.bmj.com/site/about/guidelines.xhtml

Acknowledgments

- The English Longitudinal Study of Ageing (ELSA):

ELSA is supported by the U.S. National Institute of Aging, the National Centre for Social Research, the University College London (UCL), and the Institute for Fiscal Studies. The authors gratefully acknowledge the UK Data Service and UCL who provided data for this paper.

- The Study on Global Ageing and Adult Health (SAGE):

The SAGE study is funded by the U.S. National Institute on Aging and has received financial support through Interagency Agreements (OGHA 04034785; YA1323-08-CN-0020; Y1-AG-1005-01) and Grants (R01-AG034479; IR21-AG034263-0182). The authors gratefully acknowledge the World Health Organization who provided data for this paper.

- The Survey of Health, Ageing and Retirement in Europe (SHARE):

The SHARE study is funded by the European Commission through FP5 (QLK6-CT-2001-00360), FP6 (SHARE-I3: RII-CT-2006-062193, COMPARE: CIT5-CT-2005-028857, SHARELIFE: CIT4-CT-2006-028812) and FP7 (SHARE-PREP: N°211909, SHARE-LEAP: N°227822, SHARE M4: N°261982). Additional funding from the German Ministry of Education and Research, the Max Planck Society for the Advancement of Science, the U.S. National Institute on Aging (U01_AG09740-13S2, P01_AG005842, P01_AG08291, P30_AG12815, R21_AG025169, Y1-AG-4553-01, IAG_BSR06-11, OGHA_04-064, HHSN271201300071C) and from various national funding sources is gratefully acknowledged (see www.share-project.org).

Funding: This work was supported by the five-year Ageing Trajectories of Health: Longitudinal Opportunities and Synergies (ATHLOS) project. The ATHLOS project has received funding from the European Union's Horizon 2020 research and innovation program under grant agreement No

For peer review only - http://bmjopen.bmj.com/site/about/guidelines.xhtml

635316. DF's work has been supported by grant RTI2018-100927-J-I00 Retos Investigación from Ministerio de Ciencia e Innovación (MCI), by Marsden grant E2987-3648 (Royal Society of New Zealand), and by grant 2017 SGR 622 (GRBIO) from the Departament d'Economia i Coneixement de la Generalitat de Catalunya (Spain). This work, grant number RTI2018-100927-J-I00, is supported by the Ministerio de Ciencia e Innovación (MCI, Spain), by the Agencia Estatal de Investigación (AEI, Spain) and by the European Regional Development Fund FEDER (FEDER, UE).

Competing interests: The authors report no conflicts of interest.

Patient consent for publication: not required

Ethics approval: The study protocol was approved by the Committee on the Ethics of Clinical Research, CEIC Fundació Sant Joan de Déu (Protocol No: PIC-22-15). All data were anonymized and EHR confidentially was respected in accordance with national and international law.

Data sharing statement: The original studies data are available on their respective websites: the Study on Global Ageing and Adult Health - SAGE (https://www.who.int/healthinfo/sage/en/), the English Longitudinal Study of Ageing – ELSA (https://www.elsa-project.ac.uk/), and the Survey of Health, Ageing and Retirement in Europe – SHARE (http://www.share-project.org/home0.html). R codes for harmonizing the included variables, as well as the STATA codes for the performed analysis are available on https://github.com/athlosproject/athlos-project.github.io

For peer review only

Additional files:

Additional file 1: **Table S1.** Proportion of missingness in the variables of interest in Mexico; **Table S2.** Overall proportion of missingness for indicators of latent classes and variables included in the imputation model; **Table S3.** Proportion of missingness for indicators of latent classes and variables included in the imputation model for region 1 (SAGE study - Africa: Ghana, South Africa); **Table S4.** Proportion of missingness for indicators of latent classes and variables included in the imputation

model for region 2 (SAGE study - Eastern Asia: China); **Table S5.** Proportion of missingness for indicators of latent classes and variables included in the imputation model for region 2 (SAGE study - Southern Asia: India); **Table S6.** Proportion of missingness for indicators of latent classes and variables included in the imputation model for region 2 (SAGE study - Eastern Europe: the Russian Federation); **Table S7.** Proportion of missingness for indicators of latent classes and variables included in the imputation model for region 2 (ELSA study- England); **Table S8.** Proportion of missingness for indicators of latent classes and variables included in the imputation model for region 2 (SHARE study - Northern Europe: Denmark, Sweden); **Table S9.** Proportion of missingness for indicators of latent classes and variables included in the imputation model for region 2 (SHARE study - Southern Europe: Greece, Italy, Spain); **Table S10.** Proportion of missingness for indicators of latent classes and variables included in the imputation model for region 2 (SHARE study - Western Europe: Austria, Belgium, France, Germany, Israel, Netherlands, Switzerland).

Additional file 2: **Table S1.** Association between latent multimorbidity membership and outcomes in individuals aged 50-64 and ≥65

Author contributions:

IB: Participated in the database management, drafted the paper, carried out the statistical analyses and worked on the interpretation of data. She also gave final approval of the version to be published and agreed to be accountable for all aspects of the work in ensuring that questions related to the accuracy or integrity of any part of the work are appropriately investigated and resolved; **AS:** Participated in the study design, database management, statistical support and critical revision of the paper. He also gave final approval of the version to be published and agreed to be accountable for all aspects of the work in ensuring that questions related to the accuracy or integrity of any part of the work are appropriately investigated and resolved; **LE:** Participated in the interpretation of data and critical revision of the paper; **HN:** She participated in critical revision of the paper and gave final approval of the version to be published and agreed to be accountable for all aspects of the work in

For peer review only - http://bmjopen.bmj.com/site/about/guidelines.xhtml

ensuring that questions related to the accuracy or integrity of any part of the work are appropriately investigated and resolved; **MP:** Participated in the study design, and critical revision of the paper. He also gave final approval of the version to be published and agreed to be accountable for all aspects of the work in ensuring that questions related to the accuracy or integrity of any part of the work are appropriately investigated and resolved; **DF:** Participated in the study design, database management, statistical support and critical revision of the paper. He also gave final approval of the version to be published and agreed to be accountable for all aspects of the work in ensuring that questions related to the accuracy or integrity of any part of the work are appropriately investigated and resolved; **JMH:** Participated in the study design, acquisition of data, interpretation of data and critical revision of the paper. He also gave final approval of the version to be published and agreed to be accountable for all aspects of the work in ensuring that questions related to the accuracy or integrity of any part of the work are appropriately investigated and resolved; **BO:** Participated in the acquisition of data, study design, database management, and critical revision of the paper. She also gave final approval of the version to be published and agreed to be accountable for all aspects of the work in ensuring that questions related to the accuracy or integrity of any part of the work are appropriately investigated and resolved.

References

1 WHO. Ageing and Health. https://www.who.int/news-room/fact-sheets/detail/ageing-and-health (11 December 2018, date last accessed)

2 Wang H, Naghavi M, Allen C, *et al.* Global, regional, and national life expectancy, all-cause mortality, and cause-specific mortality for 249 causes of death, 1980–2015: a systematic analysis for the Global Burden of Disease Study 2015. *Lancet* 2016;**388**:1459–544. doi:10.1016/S0140-6736(16)31012-1

For peer review only - http://bmjopen.bmj.com/site/about/guidelines.xhtml

3 Multimorbidity: a priority for global health research. 2018.

4 Parekh AK, Barton MB. The challenge of multiple comorbidity for the us health care system. *JAMA - J Am Med Assoc* 2010;**303**:1303–4. doi:10.1001/jama.2010.381

5 Mugisha J, Rosenbaum S, Ward PB, *et al.* Chronic physical conditions, multimorbidity and physical activity across 46 low- and middle-income countries. *Int J Behav Nutr Phys Act* 2017;**14**. doi:10.1186/s12966-017-0463-5

6 Chatterji S, Byles J, Cutler D, *et al.* Health, functioning, and disability in older adults - Present status and future implications. *Lancet* 2015;**385**:563–75. doi:10.1016/S0140-6736(14)61462-8

7 Arokiasamy P, Uttamacharya U, Jain K, *et al.* The impact of multimorbidity on adult physical and mental health in low- and middle-income countries: What does the study on global ageing and adult health (SAGE) reveal? *BMC Med* 2015;**13**:1–16. doi:10.1186/s12916-015-0402-8

8 Bao J, Chua KC, Prina M, *et al.* Multimorbidity and care dependence in older adults: A longitudinal analysis of findings from the 10/66 study. *BMC Public Health* 2019;**19**:1–10. doi:10.1186/s12889-019-6961-4

9 Maximova T, Pati S, Biritwum RB, *et al.* The impact of multimorbidity on adult physical and mental health in low- and middle-income countries: what does the study on global ageing and adult health (SAGE) reveal? *BMC Med* 2015;**13**:1–16. doi:10.1186/s12916-015-0402-8

10 Afshar S, Roderick PJ, Kowal P, *et al.* *Applied demography and public health in the 21st century*. Cham, Switzerland: : Springer International Publishing AG 2016. doi:10.1249/mss.0000000000001652

11 Yen L, Valderas JM, McRae IS, *et al.* Multimorbidity and comorbidity of chronic diseases among the senior Australians: prevalence and patterns. *PLoS One* 2014;**9**:e83783.

For peer review only

doi:10.1371/journal.pone.0083783

12 Baldo V, Corti MC, Boccuzzo G, *et al.* Multimorbidity patterns in high-need, high-cost elderly patients. *PLoS One* 2018;**13**:e0208875. doi:10.1371/journal.pone.0208875

13 Larsen FB, Pedersen MH, Friis K, *et al.* A Latent class analysis of multimorbidity and the relationship to socio-demographic factors and health-related quality of life. A national population-based study of 162,283 Danish Adults. *PLoS One* 2017;**12**:1–17. doi:10.1371/journal.pone.0169426

14 Alcalá-Nalvaiz JT, Sicras-Mainar A, Calderón-Larrañaga A, *et al.* Multimorbidity Patterns in Primary Care: Interactions among Chronic Diseases Using Factor Analysis. *PLoS One* 2012;**7**:e32190. doi:10.1371/journal.pone.0032190

15 Guisado-Clavero M, Roso-Llorach A, López-Jimenez T, *et al.* Multimorbidity patterns in the elderly: A prospective cohort study with cluster analysis. *BMC Geriatr* 2018;**18**:1–11. doi:10.1186/s12877-018-0705-7

16 Sanchez-Niubo A, Egea-Cortés L, Olaya B et al. Cohort profile: the ageing trajectories of health - longitudinal ppportunities and synergies (ATHLOS) project. *Int J Epidemiol* 2019;**0**:1–11. doi:org/10.1093/ije/dyz077

17 Kowal P, Chatterji S, Naidoo N, *et al.* Data resource profile: The World Health Organization study on global AGEing and adult health (SAGE). *Int J Epidemiol* 2013;**41**:1639–49. doi:10.1093/ije/dys210

18 Steptoe A, Breeze E, Banks J, *et al.* Cohort profile: The English Longitudinal Study of Ageing. *Int J Epidemiol* 2013;**42**:1640–8. doi:10.1093/ije/dys168

19 Börsch-Supan A, Brandt M, Hunkler C, *et al.* Data resource profile: The survey of health, ageing and retirement in europe (SHARE). *Int J Epidemiol* 2013;**42**:992–1001.

For peer review only - http://bmjopen.bmj.com/site/about/guidelines.xhtml

doi:10.1093/ije/dyt088

20 IDA TWBI. World Bank Open Data. https://data.worldbank.org/ (5 March 2019, date last accessed)

21 Fortier I, Raina P, Van den Heuvel ER, Griffith LE, Craig C, Saliba M, Doiron D, Stolk RP, Knoppers BM, Ferretti V, Granda P BP. Maelstrom Research guidelines for rigorous retrospective data harmonization. 2017;:103–5. doi:10.1093/ije/dyw075

22 Ronald C. Kessler TBÜ. The World Mental Health (WMH) survey initiative version of the World Health Organization (WHO) Composite Diagnostic Interview (CIDI). *Int J Methods Psychiatr Res* 2004;**13**:93–121. doi:org/10.1002/mpr.168

23 Radloff L S. The CES-D scale: A self report depression scale for research in the general population. *Appl Psychol Meas* 1977;**1**:385–401. doi:10.1002/ardp.19552881111

24 Reischies F, Lobo A, Turrina C, *et al.* Development of the EURO–D scale – a European Union initiative to compare symptoms of depression in 14 European centres. *Br J Psychiatry* 2008;**174**:330–8. doi:10.1192/bjp.174.4.330

25 Zivin K, Llewellyn DJ, Lang IA, *et al.* Depression among older adults in the United States and England. *Am J Geriatr Psychiatry* 2010;**18**:1036–44. doi:10.1097/JGP.0b013e3181dba6d2

26 Prince MJ, Beekman ATF, Deeg DJH, *et al.* Depression symptoms in late life assessed during the EURO-D scale. Effect of age, gender and marital status in 14 European centres. *Br J Psychiatry* 1999;**174**:339–45. doi:10.1192/bjp.174.4.339

27 Morris JC, Heyman A, Mohs RC, Hughes JP, van Belle G, Fillenbaum G et al. The consortium to establish a registry for Alzheimer's disease (CERAD). Part I. Clinical and neuropsychological assesment of Alzheimer's disease. *Neurology* 1989;**39**:1159–65. doi:10.1212/wnl.39.9.1159

28 WHO. Country groupings.

For peer review only

https://www.who.int/quantifying_ehimpacts/global/ebdcountgroup/en/ (7 February 2019, date last accessed)

29 United Nations Statistical Division (UNSD). Countries or areas/geographical regions. https://unstats.un.org/unsd/methodology/m49/ (7 February 2019, date last accessed)

30 Lanza ST, Dziak JJ, Huang L WA, Collins L. *LCA Stata plugin users ' guide (version 1.2.1)*. Penn State: : University Park: The Methodology Center 2018.

31 Tein JY, Coxe S, Cham H. Statistical power to detect the correct number of classes in latent profile analysis. *Struct Equ Model* 2013;**20**:640–57. doi:10.1080/10705511.2013.824781

32 Nylund-Gibson K, Choi AY. Ten frequently asked questions about latent class analysis. *Transl Issues Psychol Sci* 2018;**4**:440–61. doi:10.1037/tps0000176

33 Masyn KE. Latent Class Analysis and Finite Mixture Modeling. Published Online First: 21 March 2013. doi:10.1093/OXFORDHB/9780199934898.013.0025

34 StataCorp. *Stata: Release 13. Statistical Software*. College St. 2013.

35 Rubin DB. *Multiple Imputation for nonresponse in surveys*. United States of America: : John Wiley & Sons 1987. doi:10.2307/3172772

36 Garin N, Olaya B, Perales J, *et al.* Multimorbidity patterns in a national representative sample of the Spanish adult population. *PLoS One* 2014;**9**:e84794. doi:10.1371/journal.pone.0084794

37 Olaya B, Moneta MV, Caballero FF, *et al.* Latent class analysis of multimorbidity patterns and associated outcomes in Spanish older adults: A prospective cohort study. *BMC Geriatr* 2017;**17**. doi:10.1186/s12877-017-0586-1

38 Whitson HE, Johnson KS, Sloane R, *et al.* Identifing patterns of multimorbidity in older americans: application of latent class analysis. *J Am Geriatr Soc* 2016;**64**:1668–73.

For peer review only

doi:10.1111/jgs.14201.Identifying

39 Prados-Torres A, Calderón-Larrañaga A, Hancco-Saavedra J, *et al.* Multimorbidity patterns: A systematic review. *J Clin Epidemiol* 2014;**67**:254–66. doi:10.1016/j.jclinepi.2013.09.021

40 Alberti KGM, Zimmet P, Shaw J. Metabolic syndrome -a new world-wide definition. A consensus statement from the International Diabetes Federation. *Abstr Appl Anal* 2006;**23**:469–80. doi:10.1155/2010/307409

41 Margaretten M, Julian L, Katz P, *et al.* Depression in patients with rheumatoid arthritis: description, causes and mechanisms. *Int J Clin Rheumtol* 2011;**6**:617–23. doi:10.2217/IJR.11.6.Depression

42 Druss BG, Walker ER. Mental disorders and medical comorbidity. 2011. https://www.rwjf.org/content/dam/farm/reports/issue_briefs/2011/rwjf69438/subassets/rwjf69438_1

43 Barbara Messner DB. Smoking and cardiovascular disease: Mechanisms of endothelial dysfunction and early atherogenesis. 2014;**34**:509–15. doi:10.1161/ATVBAHA.113.300156

44 Meade T, Manolios N, Cumming SR, *et al.* Cognitive impairment in rheumatoid arthritis: A systematic review. *Arthritis Care Res* 2018;**70**:39–52. doi:10.1002/acr.23243

45 Lam RW, Kennedy SH, McIntyre RS, *et al.* Cognitive dysfunction in major depressive disorder: effects on psychosocial functioning and implications for treatment. *Can J Psychiatry* 2014;**59**:649–54. doi:10.1177/070674371405901206

46 Dodd JW. Lung disease as a determinant of cognitive decline and dementia. *Alzheimer's Res Ther* 2015;**7**:1–8. doi:10.1186/s13195-015-0116-3

47 Rose Mayeda E, Whitmer RA, Yaffe K. Diabetes and cognition. *Clin Geriatr Med* 2015;**31**:101–ix. doi:10.1016/j.cger.2014.08.021

For peer review only - http://bmjopen.bmj.com/site/about/guidelines.xhtml

48 Walker KA, Power MC, Gottesman RF. Defining the relationship between hypertension, cognitive decline, and dementia: a review. 2018;**19**:24. doi:10.1007/s11906-017-0724-3

49 Deckers K, Schievink SHJ, Rodriquez MMF, *et al.* Coronary heart disease and risk for cognitive impairment or dementia: Systematic review and meta-analysis. *PLoS One* 2017;**12**:1–15. doi:10.1371/journal.pone.0184244

50 Leon DA, Shkolnikov VM, McKee M, *et al.* Alcohol increases circulatory disease mortality in Russia: Acute and chronic effects or misattribution of cause? *Int J Epidemiol* 2010;**39**:1279–90. doi:10.1093/ije/dyq102

51 Petrukhin IS, Lunina EY. Cardiovascular disease risk factors and mortality in Russia: Challenges and barriers. *Public Health Rev* 2012;**33**:436–49. doi:10.1007/BF03391645

52 Y. He, M. Zhang, E. H. B. Lin et al. Mental disorders among persons with arthritis: results from the World Mental Health Surveys. 2008;**38**:1639–50. doi:10.1017/S0033291707002474

53 Jamrozik E, Musk AW. Respiratory health issues in the Asia-Pacific region: An overview. *Respirology* 2011;**16**:3–12. doi:10.1111/j.1440-1843.2010.01844.x

54 Sandeep Grover, Alakananda Dutt AA. An overview of Indian research in depression. *Indian J Psychiatry* 2010;**52**:178–88. doi:10.4103/0019-5545.69231

55 Gu L, Xie J, Long J, *et al.* Epidemiology of major depressive disorder in Mainland China: A Systematic review. *PLoS One* 2013;**8**:e65356. doi:10.1371/journal.pone.0065356

56 Hou Z, Jiang W, Yin Y, *et al.* The current situation on major depressive disorder in China: Research on mechanisms and clinical practice. *Neurosci Bull* 2016;**32**:389–97. doi:10.1007/s12264-016-0037-6

57 Castro-Costa E, Dewey M, Stewart R, *et al.* Ascertaining late-life depressive symptoms in Europe: An evaluation of the survey version of the EURO-D scale in 10 nations. The SHARE

For peer review only - http://bmjopen.bmj.com/site/about/guidelines.xhtml

project. *Int J Methods Psychiatr Res* 2008;**17**:12–29. doi:10.1002/mpr.236

58 Huntley AL, Johnson R, Purdy S, *et al.* Measures of multimorbidity and morbidity burden for use in primary care and comminuty settings: A systematic review and guide. *Ann Fam Med* 2012;**10**:134–41. doi:10.1370/afm.1363

59 Dal Grande E, Fullerton S, Taylor AW. Reliability of self-reported health risk factors and chronic conditions questions collected using the telephone in South Australia, Australia. *BMC Med Res Methodol* 2012;**12**. doi:10.1186/1471-2288-12-108

60 Ortolá-Devesa JB, Egea-Caparrós JM, Huerta JM, *et al.* Accuracy of self-reported diabetes, hypertension, and hyperlipidemia in the adult spanish population. DINO study findings. *Rev Española Cardiol (English Ed* 2009;**62**:143–52. doi:10.1016/s1885-5857(09)71532-4

61 Martin LM, Leff M, Calonge N, *et al.* Validation of self-reported chronic conditions and health services in a managed care population. *Am J Prev Med* 2000;**18**:215–8. doi:10.1016/S0749-3797(99)00158-0

62 Finch H. A comparison of statistics for assessing model invariance in latent class analysis. *Open J Stat* 2015;**5**:191–210. doi:10.4236/ojs.2015.53022

63 White IR, Carlin JB. Bias and efficiency of multiple imputation compared with complete-case analysis for missing covariate values. *Stat Med* 2010;**29**:2920–31. doi:10.1002/sim.3944

64 Sterne JAC, White IR, Carlin JB, *et al.* Multiple imputation for missing data in epidemiological and clinical research: Potential and pitfalls. *BMJ* 2009;**339**:157–60. doi:10.1136/bmj.b2393

For peer review only

For peer review only - http://bmjopen.bmj.com/site/about/guidelines.xhtml

Figure 1 Prevalence of diseases in the three latent classes in the total sample and by regions (subsample 50-64 years)

Figure 2 Prevalence of diseases in the three latent classes in the total sample and by regions (subsample +65 years)

For peer review only

For peer review only - http://bmjopen.bmj.com/site/about/guidelines.xhtml

Multimorbidity latent classes

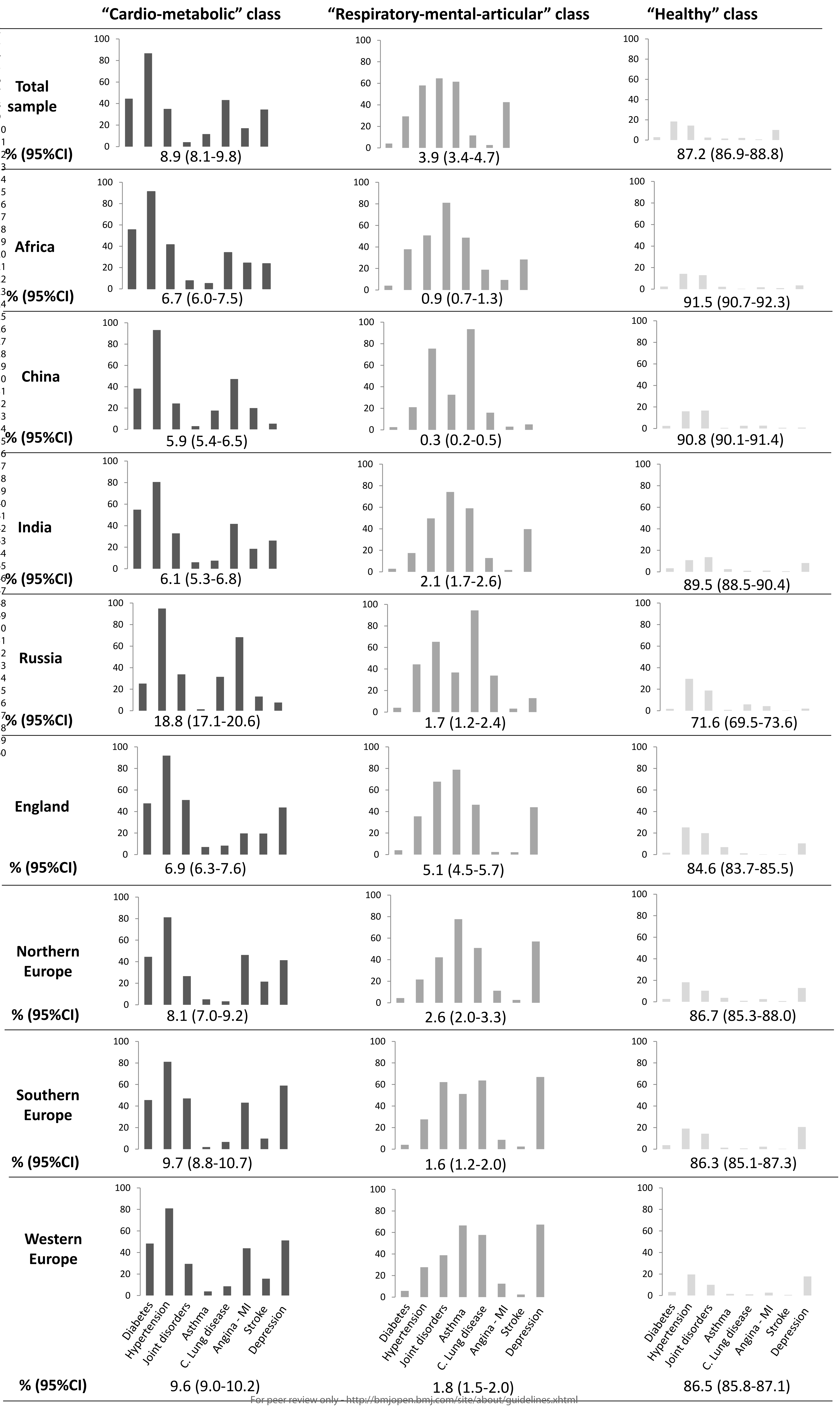

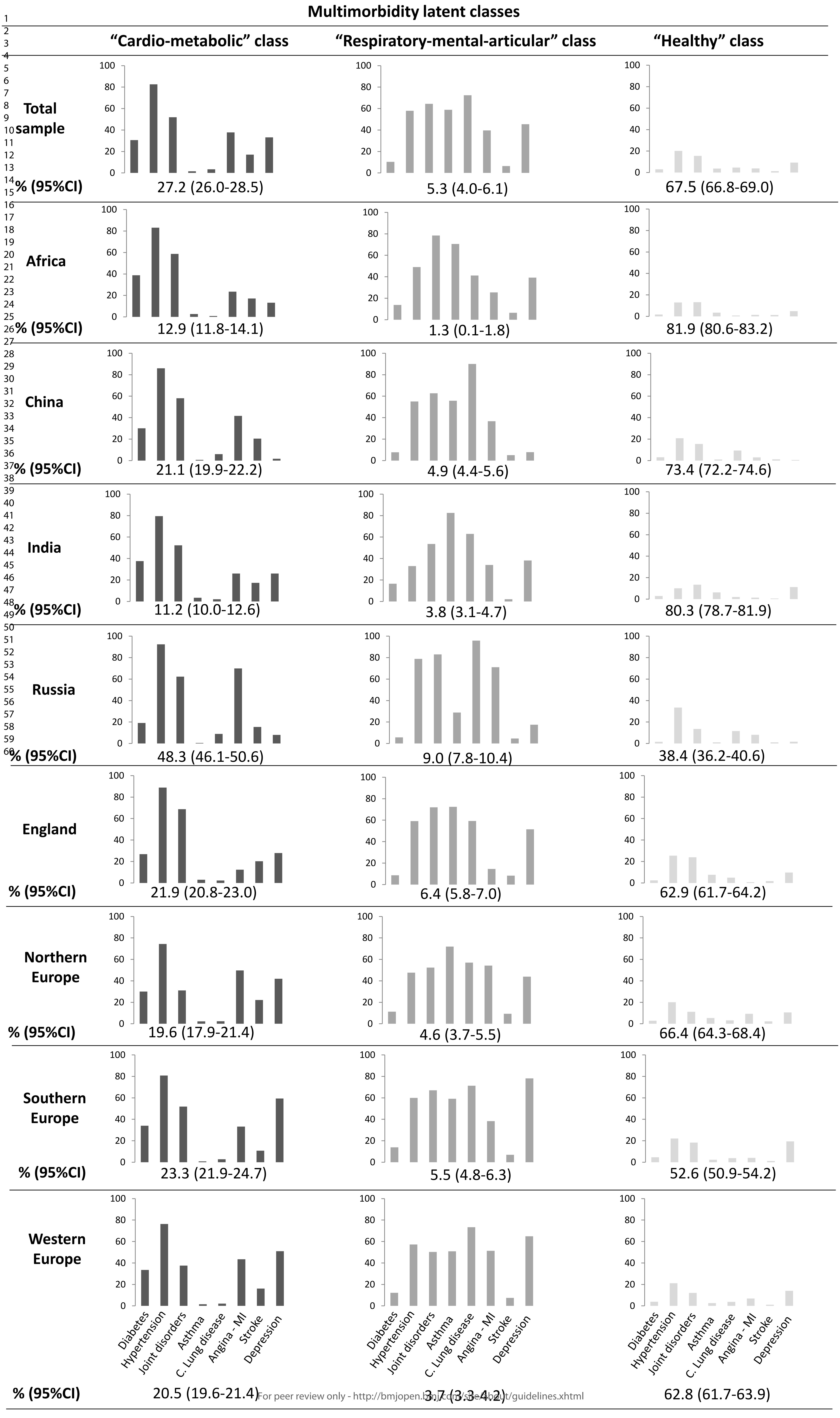
Multimorbidity latent classes
"Cardio-metabolic" class
"Respiratory-mental-articular" class
"Healthy" class
Total sample
% (95%CI)
27.2 (26.0-28.5)
5.3 (4.0-6.1)
67.5 (66.8-69.0)
Africa
% (95%CI)
12.9 (11.8-14.1)
1.3 (0.1-1.8)
81.9 (80.6-83.2)
China
% (95%CI)
21.1 (19.9-22.2)
4.9 (4.4-5.6)
73.4 (72.2-74.6)
India
% (95%CI)
11.2 (10.0-12.6)
3.8 (3.1-4.7)
80.3 (78.7-81.9)
Russia
% (95%CI)
48.3 (46.1-50.6)
9.0 (7.8-10.4)
38.4 (36.2-40.6)
England
% (95%CI)
21.9 (20.8-23.0)
6.4 (5.8-7.0)
62.9 (61.7-64.2)
Northern Europe
% (95%CI)
19.6 (17.9-21.4)
4.6 (3.7-5.5)
66.4 (64.3-68.4)
Southern Europe
% (95%CI)
23.3 (21.9-24.7)
5.5 (4.8-6.3)
52.6 (50.9-54.2)
Western Europe
% (95%CI)
20.5 (19.6-21.4)
3.7 (3.3-4.2)
62.8 (61.7-63.9)
Diabetes
Hypertension
Joint disorders
Asthma
C. Lung disease
Angina - MI
Stroke
Depression
0
20
40
60
80
100

Table S1. Proportion of missingness in the variables of interest in Mexico

	n (%) missingness
**(a) Indicators of multimorbidity classes**	
Diabetes	2814 (51.65)
Hypertension	2815 (51.67)
Joint disorders	2814 (51.65)
Asthma	2814 (51.65)
Chronic lung disease	2814 (51.65)
Myocardial infarction – Angina	2814 (51.65)
Stroke	2814 (51.65)
Depression	2815 (51.67)
**(b) Variables used in the regression model**	
Sex	2706 (49.67)
Age	2707 (49.69)
Country	0 (0.00)
Study	0 (0.00)
Marital status	2811 (51.60)
Education	3264 (59.91)
Wealth	1922 (35.28)
Loneliness	2819 (51.74)
Ever smoked	2814 (51.65)
Vigorous exercise in last 2 weeks	2814 (51.65)
Self-rated health	2811 (51.6)
ADL – Using the toilet	2814 (51.65)
ADL – Bathing or showering	2815 (51.67)
ADL – Getting dressed	0 (0.00)
ADL - Eating	2814 (51.65)
ADL – Getting in or out of bed	2813 (51.63)
ADL – Moving around the house	2817 (51.71)
Memory: Immediate recall	2854 (52.39)
Memory: Delayed recall	2854 (52.39)
Verbal fluency	2852 (52.35)

For peer review only

Table S2. Overall proportion of missingness for indicators of latent classes and variables included in the imputation model

(a) Indicators of multimorbidity classes	
	n (%) missingness
Diabetes	62 (0.08)
Hypertension	154 (0.21)
Joint disorders	31 (0.04)
Asthma	82 (0.11)
Chronic lung disease	51 (0.07)
Myocardial infarction – Angina	63 (0.08)
Stroke	41 (0.05)
Depression	1233 (1.70)
(b) Variables used in the regression model	
Sex	0 (0.00)
Age	0 (0.00)
Country	0 (0.00)
Study	0 (0.00)
Marital status	106 (0.14)
Education	11475 (15.91)
Wealth	844 (1.17)
Loneliness	10985 (15.23)
Ever smoked	201 (0.27)
Vigorous exercise in last 2 weeks	258 (0.35)
Self-rated health	218 (0.30)
ADL – Using the toilet	355 (0.49)
ADL – Bathing or showering	340 (0.47)
ADL – Getting dressed	304 (0.42)
ADL - Eating	400 (0.55)
ADL – Getting in or out of bed	310 (0.42)
ADL – Moving around the house	345 (0.47)
Memory: Immediate recall	1442 (1.99)
Memory: Delayed recall	1448 (2.00)
Verbal fluency	1641 (2.27)

For peer review only

Table S3. Proportion of missingness for indicators of latent classes and variables included in the imputation model for region 1 (SAGE study - Africa: Ghana, South Africa)

(a) Indicators of multimorbidity classes	
	n (%) missingness
Diabetes	1 (0.01)
Hypertension	1 (0.01)
Joint disorders	3 (0.03)
Asthma	1 (0.01)
Chronic lung disease	1 (0.01)
Myocardial infarction – Angina	3 (0.03)
Stroke	1 (0.01)
Depression	17 (0.21)
(b) Variables used in the regression model	
Sex	0 (0.00)
Age	0 (0.00)
Country	0 (0.00)
Study	0 (0.00)
Marital status	89 (1.11)
Education	3705 (46.6)
Wealth	23 (0.28)
Loneliness	48 (0.60)
Ever smoked	7 (0.08)
Vigorous exercise in last 2 weeks	12 (0.15)
Self-rated health	15 (0.18)
ADL – Using the toilet	40 (0.50)
ADL – Bathing or showering	32 (0.40)
ADL – Getting dressed	27 (0.33)
ADL - Eating	31 (0.38)
ADL – Getting in or out of bed	32 (0.40)
ADL – Moving around the house	35 (0.44)
Memory: Immediate recall	36 (0.45)
Memory: Delayed recall	31 (0.38)
Verbal fluency	9 (0.11)

For peer review only

Table S4. Proportion of missingness for indicators of latent classes and variables included in the imputation model for region 2 (SAGE study - Eastern Asia: China)

(a) Indicators of multimorbidity classes	
	n (%) missingness
Diabetes	35 (0.27)
Hypertension	127 (0.98)
Joint disorders	2 (0.01)
Asthma	53 (0.41)
Chronic lung disease	23 (0.17)
Myocardial infarction – Angina	34 (0.26)
Stroke	13 (0.10)
Depression	146 (1.13)
**(b) Variables used in the regression model**	
Sex	0 (0.00)
Age	0 (0.00)
Country	0 (0.00)
Study	0 (0.00)
Marital status	8 (0.06)
Education	3105 (24.18)
Wealth	18 (0.14)
Loneliness	74 (0.57)
Ever smoked	2 (0.01)
Vigorous exercise in last 2 weeks	45 (0.35)
Self-rated health	16 (0.12)
ADL – Using the toilet	51 (0.39)
ADL – Bathing or showering	52 (0.40)
ADL – Getting dressed	41 (0.31)
ADL - Eating	46 (0.35)
ADL – Getting in or out of bed	36 (0.28)
ADL – Moving around the house	48 (0.37)
Memory: Immediate recall	317 (2.46)
Memory: Delayed recall	399 (3.10)
Verbal fluency	335 (2.60)

For peer review only

Table S5. Proportion of missingness for indicators of latent classes and variables included in the imputation model for region 2 (SAGE study - Southern Asia: India)

(a) Indicators of multimorbidity classes	
	n (%) missingness
Diabetes	0 (0.00)
Hypertension	1 (0.01)
Joint disorders	1 (0.01)
Asthma	0 (0.00)
Chronic lung disease	0 (0.00)
Myocardial infarction – Angina	0 (0.00)
Stroke	1 (0.01)
Depression	1 (0.01)
(b) Variables used in the regression model	
Sex	0 (0.00)
Age	0 (0.00)
Country	0 (0.00)
Study	0 (0.00)
Marital status	0 (0.00)
Education	0 (0.00)
Wealth	38 (0.57)
Loneliness	10 (0.15)
Ever smoked	1 (0.01)
Vigorous exercise in last 2 weeks	0 (0.00)
Self-rated health	0 (0.00)
ADL – Using the toilet	19 (0.28)
ADL – Bathing or showering	12 (0.18)
ADL – Getting dressed	11 (0.16)
ADL - Eating	73 (1.11)
ADL – Getting in or out of bed	7 (0.10)
ADL – Moving around the house	24 (0.36)
Memory: Immediate recall	88 (1.34)
Memory: Delayed recall	88 (1.34)
Verbal fluency	71 (1.08)

For peer review only

Table S6. Proportion of missingness for indicators of latent classes and variables included in the imputation model for region 2 (SAGE study - Eastern Europe: the Russian Federation)

(a) Indicators of multimorbidity classes	
	n (%) missingness
Diabetes	5 (0.12)
Hypertension	4 (0.10)
Joint disorders	3 (0.07)
Asthma	6 (0.15)
Chronic lung disease	5 (0.12)
Myocardial infarction – Angina	5 (0.12)
Stroke	5 (0.12)
Depression	14 (0.36)
(b) Variables used in the regression model	
Sex	0 (0.00)
Age	0 (0.00)
Country	0 (0.00)
Study	0 (0.00)
Marital status	6 (0.15)
Education	41 (1.05)
Wealth	5 (0.12)
Loneliness	79 (2.03)
Ever smoked	1 (0.02)
Vigorous exercise in last 2 weeks	0 (0.00)
Self-rated health	5 (0.12)
ADL – Using the toilet	47 (1.20)
ADL – Bathing or showering	46 (1.18)
ADL – Getting dressed	27 (0.69)
ADL - Eating	52 (1.33)
ADL – Getting in or out of bed	37 (0.95)
ADL – Moving around the house	40 (1.02)
Memory: Immediate recall	153 (3.93)
Memory: Delayed recall	81 (2.08)
Verbal fluency	208 (5.35)

For peer review only

Table S7. Proportion of missingness for indicators of latent classes and variables included in the imputation model for region 2 (ELSA study- England)

(a) Indicators of multimorbidity classes	
	n (%) missingness
Diabetes	2 (0.01)
Hypertension	2 (0.01)
Joint disorders	3 (0.02)
Asthma	3 (0.02)
Chronic lung disease	3 (0.02)
Myocardial infarction – Angina	2 (0.01)
Stroke	2 (0.01)
Depression	372 (3.23)
(b) Variables used in the regression model	
Sex	0 (0.00)
Age	0 (0.00)
Country	0 (0.00)
Study	0 (0.00)
Marital status	0 (0.00)
Education	1023 (8.88)
Wealth	330 (2.86)
Loneliness	356 (3.09)
Ever smoked	173 (1.50)
Vigorous exercise in last 2 weeks	176 (1.52)
Self-rated health	175 (1.51)
ADL – Using the toilet	173 (1.50)
ADL – Bathing or showering	173 (1.50)
ADL – Getting dressed	173 (1.50)
ADL - Eating	173 (1.50)
ADL – Getting in or out of bed	173 (1.50)
ADL – Moving around the house	173 (1.50)
Memory: Immediate recall	360 (3.12)
Memory: Delayed recall	373 (3.23)
Verbal fluency	362 (3.14)

For peer review only

Table S8. Proportion of missingness for indicators of latent classes and variables included in the imputation model for region 2 (SHARE study - Northern Europe: Denmark, Sweden)

(a) Indicators of multimorbidity classes	
	n (%) missingness
Diabetes	2 (0.04)
Hypertension	2 (0.04)
Joint disorders	2 (0.04)
Asthma	2 (0.04)
Chronic lung disease	2 (0.04)
Myocardial infarction – Angina	2 (0.04)
Stroke	2 (0.04)
Depression	78 (1.70)
(b) Variables used in the regression model	
Sex	0 (0.00)
Age	0 (0.00)
Country	0 (0.00)
Study	0 (0.00)
Marital status	0 (0.00)
Education	52 (1.13)
Wealth	6 (0.13)
Loneliness	1384 (30.26)
Ever smoked	4 (0.08)
Vigorous exercise in last 2 weeks	6 (0.13)
Self-rated health	2 (0.04)
ADL – Using the toilet	3 (0.06)
ADL – Bathing or showering	3 (0.06)
ADL – Getting dressed	3 (0.06)
ADL - Eating	3 (0.06)
ADL – Getting in or out of bed	3 (0.06)
ADL – Moving around the house	3 (0.06)
Memory: Immediate recall	68 (1.48)
Memory: Delayed recall	66 (1.44)
Verbal fluency	83 (1.81)

For peer review only

Table S9. Proportion of missingness for indicators of latent classes and variables included in the imputation model for region 2 (SHARE study - Southern Europe: Greece, Italy, Spain)

(a) Indicators of multimorbidity classes	
	n (%) missingness
Diabetes	12 (0.16)
Hypertension	12 (0.16)
Joint disorders	12 (0.16)
Asthma	12 (0.16)
Chronic lung disease	12 (0.16)
Myocardial infarction – Angina	12 (0.16)
Stroke	12 (0.16)
Depression	194 (2.59)
(b) Variables used in the regression model	
Sex	0 (0.00)
Age	0 (0.00)
Country	0 (0.00)
Study	0 (0.00)
Marital status	1 (0.01)
Education	20 (0.26)
Wealth	95 (1.27)
Loneliness	2715 (36.37)
Ever smoked	3 (0.04)
Vigorous exercise in last 2 weeks	4 (0.05)
Self-rated health	0 (0.00)
ADL – Using the toilet	3 (0.04)
ADL – Bathing or showering	3 (0.04)
ADL – Getting dressed	3 (0.04)
ADL - Eating	3 (0.04)
ADL – Getting in or out of bed	3 (0.04)
ADL – Moving around the house	3 (0.04)
Memory: Immediate recall	125 (1.67)
Memory: Delayed recall	125 (1.67)
Verbal fluency	176 (2.35)

For peer review only

Table S10. Proportion of missingness for indicators of latent classes and variables included in the imputation model for region 2 (SHARE study - Western Europe: Austria, Belgium, France, Germany, Israel, Netherlands, Switzerland)

(a) Indicators of multimorbidity classes	
	n (%) missingness
Diabetes	5 (0.02)
Hypertension	5 (0.02)
Joint disorders	5 (0.02)
Asthma	5 (0.02)
Chronic lung disease	5 (0.02)
Myocardial infarction – Angina	5 (0.02)
Stroke	5 (0.02)
Depression	411 (2.36)
(b) Variables used in the regression model	
Sex	0 (0.00)
Age	0 (0.00)
Country	0 (0.00)
Study	0 (0.00)
Marital status	2 (0.01)
Education	165 (0.95)
Wealth	329 (1.89)
Loneliness	6319 (36.42)
Ever smoked	10 (0.05)
Vigorous exercise in last 2 weeks	15 (0.08)
Self-rated health	5 (0.02)
ADL – Using the toilet	19 (0.10)
ADL – Bathing or showering	19 (0.10)
ADL – Getting dressed	19 (0.10)
ADL - Eating	19 (0.10)
ADL – Getting in or out of bed	19 (0.10)
ADL – Moving around the house	19 (0.10)
Memory: Immediate recall	295 (1.7)
Memory: Delayed recall	285 (1.64)
Verbal fluency	397 (2.28)

For peer review only

Table S1. Association between latent multimorbidity membership and outcomes in individuals aged 50-64 and ≥65

Outcomes[a]	Aged 50-64		Aged ≥65	
	"Cardio-metabolic" class	"Respiratory-mental-articular" class	"Cardio-metabolic" class	"Respiratory-mental-articular" class
Sex				
Woman	1.0	1.0	1.0	1.0
Male	0.9 (0.8-1.0)	0.6 (0.5-0.7)	0.7 (0.7-0.8)	0.9 (0.8-1.0)
Age (years)	1.1 (1.1-1.1)	1.1 (1.0-1.1)	1.0 (1.0-1.0)	1.0 (1.0-1.0)
Marital status				
Single	1.0	1.0	1.0	1.0
Married	0.9 (0.8-1.1)	0.7 (0.5-1.0)	1.2 (1.00-1.4)	1.2 (0.9-1.6)
Divorced	1.1 (0.9-1.4)	1.7 (1.2-2.4)	1.1 (1.0-1.4)	1.8 (1.2-2.6)
Widowed	1.4 (1.1-1.7)	1.1 (0.8-1.6)	1.5 (1.3-1.8)	1.6 (1.2-2.3)
Education level				
Primary or less	1.0	1.0	1.0	1.0
Secondary	0.9 (0.9-1.0)	0.7 (0.6-0.8)	1.0 (0.9-1.0)	0.7 (0.6-0.8)
Tertiary	0.8 (0.7-0.9)	0.6 (0.5-0.7)	0.9 (0.8-1.0)	0.6 (0.5-0.7)
Wealth				
1st (worse)	1.0	1.0	1.0	1.0
2nd	1.0 (0.9-1.1)	0.8 (0.6-1.0)	1.1 (1.0-1.2)	1.1 (0.9-1.2)
3rd	0.9 (0.9-1.1)	0.7 (0.5-0.8)	1.1 (1.0-1.2)	1.0 (0.9-1.2)
4th	0.9 (0.8-1.0)	0.6 (0.5-0.8)	1.1 (1.0-1.2)	0.8 (0.7-1.0)
5th (best)	0.8 (0.7-0.9)	0.5 (0.4-0.6)	1.2 (1.0-1.2)	0.7 (0.6-0.9)
Region				
Africa	1.0	1.0	1.0	1.0
China	0.9 (0.8-1.0)	0.3 (0.2-0.5)	1.8 (1.6-2.1)	4.1 (3.0-5.7)
India	0.9 (0.8-1.1)	2.2 (1.5-3.2)	0.9 (0.8-1.0)	2.9 (2.0-4.2)
Russia	3.6 (3.0-4.2)	2.2 (1.4-3.5)	8.0 (7.0-9.2)	14.5 (10.3-20.3)
England	1.1 (0.9-1.3)	5.6 (4.1-7.7)	2.2 (2.0-2.5)	6.2 (4.5-8.5)
Northern Europe	1.3 (1.1-1.5)	2.8 (1.9-4.1)	1.9 (1.6-2.2)	4.2 (2.9-6.1)
Southern Europe	1.5 (1.3-1.8)	1.7 (1.2-2.5)	2.8 (2.5-3.2)	6.5 (4.7-9.0)
Western Europe	1.5 (1.3-1.7)	1.9 (1.3-2.6)	2.1 (1.9-2.3)	3.6 (2.6-5.0)
Loneliness (yes/no)	1.9 (1.8-2.1)	2.5 (2.1-3.0)	1.4 (1.4-1.6)	1.9 (1.7-2.2)
Ever smoked (yes/no)	1.0 (0.9-1.1)	1.7 (1.5-2.0)	0.8 (0.8-0.8)	1.4 (1.3-1.6)
Physical activity (yes/no)	0.5 (0.5-0.5)	0.6 (0.5-0.7)	0.5 (0.5-0.6)	0.5 (0.4-0.6)
Limitations in ADL (yes/no)	2.5 (2.3-2.7)	3.2 (2.7-3.7)	1.8 (1.7-1.9)	2.8 (2.5-3.1)
Self-rated health				
Good	1.0	1.0	1.0	1.0
Moderate	3.7 (3.3-3.9)	2.5 (2.1-2.9)	2.5 (2.3-2.7)	3.9 (3.3-4.6)
Poor	8.5 (7.7-9.4)	7.4 (6.1-8.8)	4.3 (4.6)	10.9 (9.3-12.8)
Memory: Immediate recall	0.9 (0.9-0.9)	0.9 (0.9-1.0)	0.9 (0.9-0.9)	0.9 (0.9-0.9)
Memory: Delayed recall	0.9 (0.9-0.9)	0.9 (0.8-0.9)	0.9 (0.9-0.9)	0.9 (0.9-0.9)
Verbal fluency	1.0 (1.0-1.0)	1.0 (1.0-1.0)	1.0 (0.9-1.00)	1.0 (0.9-1.0)

Note: The reference group for the multimorbidity group variable was the "Healthy" class
Relative Risk Ratios (95% confidence interval) from multinomial logistic regression models
Models were run in 100 imputed datasets and results combined using Rubin's rules
ADL Activities of Daily Living
[a]Unadjusted analyses.

For peer review only

STROBE CHECKLIST: "MULTIMORBIDITY PATTERNS IN LOW-MIDDLE AND HIGH INCOME REGIONS: A MULTI-REGION LATENT CLASS ANALYSIS USING ATHLOS HARMONIZED COHORTS"

1. Title and abstract

(a) Indicate the study's design with a commonly used term in the title or the abstract **(page 1 and 3)**

(b) Provide in the abstract an informative and balanced summary of what was done and what was found **(page 3)**

Introduction

2. Background/rationale: Explain the scientific background and rationale for the investigation being reported **(page 5-6)**

3. Objectives: State specific objectives, including any prespecified hypotheses **(page 6, lines 10-13)**

Methods

4. Study design: Present key elements of study design early in the paper **(page 6, lines 16-19)**

5. Setting: Describe the setting, locations, and relevant dates, including periods of recruitment, exposure, follow-up, and data collection **(page 6, lines 20-24, page 7, lines 1-3).**

6. Participants: Give the eligibility criteria, and the sources and methods of selection of Participants **(page 7, lines 4-9)**.

7. Variables: Clearly define all outcomes, exposures, predictors, potential confounders, and effect modifiers. Give diagnostic criteria, if applicable. **(page 7, lines 13- page 8)**.

8. Data sources/measurement: For each variable of interest, give sources of data and details of methods of assessment (measurement). Describe comparability of assessment methods if there is more than one group **(page 7, lines 13- page 8).**

9. Bias: Describe any efforts to address potential sources of bias **(page 9, lines 19-23).**

10. Study size: Explain how the study size was arrived at **(page 7, lines 4-9)**.

11. Quantitative variables: Explain how quantitative variables were handled in the analyses. If applicable, describe which groupings were chosen and why. **(page 7, lines 13- page 8)**.

12. Statistical methods **(page 9-10)**:

(a) Describe all statistical methods, including those used to control for confounding

(b) Describe any methods used to examine subgroups and interactions

(c) Explain how missing data were addressed

(d) If applicable, describe analytical methods taking account of sampling strategy

(e) Describe any sensitivity analyses

Results

13. Participants **(page 7, lines 4-9)**:

(a) Report numbers of individuals at each stage of study—eg numbers potentially eligible, examined for eligibility, confirmed eligible, included in the study, completing follow-up, and analysed
(b) Give reasons for non-participation at each stage
(c) Consider use of a flow diagram

14. Descriptive data **(page 10, lines 8-18; page 11, lines 1-7)**:

(a) Give characteristics of study participants (eg demographic, clinical, social) and information on exposures and potential confounders
(b) Indicate number of participants with missing data for each variable of interest **(Suplemmentary file 1)**

15. Outcome data: Report numbers of outcome events or summary measures **(pages 12-15)**.
16. Main results **(page 15, lines 14-21; to page 17)**:

(a) Give unadjusted estimates and, if applicable, confounder-adjusted estimates and their precision (eg, 95% confidence interval). Make clear which confounders were adjusted for and why they were included
(b) Report category boundaries when continuous variables were categorized
(c) If relevant, consider translating estimates of relative risk into absolute risk for a meaningful time period

17. Other analyses: Report other analyses done—eg analyses of subgroups and interactions, and sensitivity analyses. **Not applicable.**

Discussion

18. Key results: Summarise key results with reference to study objectives **(page 17, lines 5-8)**.
19. Limitations: Discuss limitations of the study, taking into account sources of potential bias or imprecision. Discuss both direction and magnitude of any potential bias **(page 21, lines 19-24, page 22, 1-14).**
20. Interpretation: Give a cautious overall interpretation of results considering objectives, limitations, multiplicity of analyses, results from similar studies, and other relevant evidence **(pages 18-21)**.
21. Generalisability: Discuss the generalisability (external validity) of the study results **(page 19, 13-15)**.

Other information

22. Funding: Give the source of funding and the role of the funders for the present study and, if applicable, for the original study on which the present article is based **(page 23, lines 20-22, page 24, lines 1-6)**.

For peer review only

BMJ Open

Multimorbidity patterns in low-middle and high income regions: A multi-region latent class analysis using ATHLOS harmonized cohorts.

Journal:	*BMJ Open*
Manuscript ID	bmjopen-2019-034441.R2
Article Type:	Original research
Date Submitted by the Author:	17-May-2020

Complete List of Authors:	Bayes-Marin, Ivet; Parc Sanitari Sant Joan de Déu, Research, Innovation and Teaching Unit; Centro de Investigación Biomédica en Red de Salud Mental, CIBERSAM<br>Sanchez-Niubo, Albert ; Parc Sanitari Sant Joan de Déu, Research, Innovation and Teaching Unit; Centro de Investigación Biomédica en Red de Salud Mental, CIBERSAM<br>Egea-Cortés, Laia; Parc Sanitari Sant Joan de Déu, Research, Innovation and Teaching Unit<br>Nguyen, Hai; King's College London, Health Service and Population Research Department<br>Prina, A.Matthew; King's College London,<br>Fernández, Daniel; Parc Sanitari Sant Joan de Déu, Research, Innovation and Teaching Unit; CIBERSAM<br>Haro, Josep; Parc Sanitari Sant Joan de Deu, CIBERSAM, University of Barcelona; King Sand University<br>Olaya, Beatriz; Parc Sanitari Sant Joan de Deu, Research and Development Unit

**Primary Subject Heading**:	Epidemiology
Secondary Subject Heading:	Global health
Keywords:	multimorbidity, non-communicable diseases (NCDs), low- and middle-income countries (LMICs), high-income country (HICs), Latent class analysis (LCA)

SCHOLARONE™
Manuscripts

I, the Submitting Author has the right to grant and does grant on behalf of all authors of the Work (as defined in the below author licence), an exclusive licence and/or a non-exclusive licence for contributions from authors who are: i) UK Crown employees; ii) where BMJ has agreed a CC-BY licence shall apply, and/or iii) in accordance with the terms applicable for US Federal Government officers or employees acting as part of their official duties; on a worldwide, perpetual, irrevocable, royalty-free basis to BMJ Publishing Group Ltd ("BMJ") its licensees and where the relevant Journal is co-owned by BMJ to the co-owners of the Journal, to publish the Work in this journal and any other BMJ products and to exploit all rights, as set out in our licence.

The Submitting Author accepts and understands that any supply made under these terms is made by BMJ to the Submitting Author unless you are acting as an employee on behalf of your employer or a postgraduate student of an affiliated institution which is paying any applicable article publishing charge ("APC") for Open Access articles. Where the Submitting Author wishes to make the Work available on an Open Access basis (and intends to pay the relevant APC), the terms of reuse of such Open Access shall be governed by a Creative Commons licence – details of these licences and which Creative Commons licence will apply to this Work are set out in our licence referred to above.

Other than as permitted in any relevant BMJ Author's Self Archiving Policies, I confirm this Work has not been accepted for publication elsewhere, is not being considered for publication elsewhere and does not duplicate material already published. I confirm all authors consent to publication of this Work and authorise the granting of this licence.

For peer review only

Original Research Article

Manuscript title:

Multimorbidity patterns in low-middle and high income regions: A multi-region latent class analysis using ATHLOS harmonized cohorts.

Author's full names and affiliations:

Ivet Bayes-Marin, [a,b,c] Albert Sanchez-Niubo, [a,b] Laia Egea-Cortés, [d] Hai Nguyen, [e] Matthew Prina, [e] Daniel Fernández, [a,b,f] Josep Maria Haro, [a,b,c] and Beatriz Olaya [a,b]

From the [a]Research, Innovation and Teaching Unit, Parc Sanitari Sant Joan de Déu, Sant Boi de Llobregat, Spain, [b]Centro de Investigación Biomédica en Red de Salud Mental, CIBERSAM, Madrid, Spain, [c]Department of Medicine, Universitat de Barcelona, Barcelona, Spain, [d]Center for Epidemiological Studies on Sexually Transmitted Infections and HIV/AIDS of Catalonia (CEEISCAT), Catalonia Public Health Agency (ASPCAT), Badalona, Spain, [e]Health Service and Population Research Department, Institute of Psychiatry, Psychology and Neuroscience, King's College London, London, United Kingdom, [f]Serra Húnter fellow. Department of Statistics and Operations Research, Polytechnic University of Catalonia, Barcelona, Spain.

Corresponding author details:

Correspondence: Albert Sanchez- Niubo. Research, Innovation and Teaching Unit, Parc Sanitari Sant Joan de Déu, Dr Antoni Pujadas 42, Sant Boi de Llobregat, Barcelona 08830, Spain. E-mail: albert.sanchez@pssjd.org

ORCID of the authors:

Ivet Bayes-Marin - 0000-0002-3816-5244

For peer review only

Albert Sanchez-Niubo - 0000-0003-0309-181X

Laia Egea-Cortés - 0000-0003-4002-8254

Hai Nguyen - 0000-0003-2171-1955

Matthew Prina - 0000-0001-6698-3263

Daniel Fernández - 0000-0003-0012-2094

Josep Maria Haro - 0000-0002-3984-277X

Beatriz Olaya - 0000-0003-2046-3929

Word count (excluding title page, abstract, references, figures and tables): 5183

Abstract

Objectives: Our aim was to determine clusters of non-communicable diseases (NCDs) in a very large, population-based sample of middle-aged and older adults from low- and middle-income (LMICs) and high-income (HICs) regions. Additionally, we explored the associations with several covariates.

Design: The total sample was 72 140 people aged 50+ from three population-based studies (ELSA, SHARE and SAGE) included in the ATHLOS project and representing eight regions with LMICs and HICs. Variables were previously harmonized using an ex-post strategy. Eight NCDs were used in latent class analysis. Multinomial models were made to calculate associations with covariates. All the analyses were stratified by age (50-64 and 65+ years old).

Results: Three clusters were identified: "cardio-metabolic" (8.93% in participants aged 50-64 and 27.22% in those aged 65+), "respiratory-mental-articular" (3.91% and 5.27%) and "healthy" (87.16% and 67.51%). In the younger group, Russia presented the highest prevalence of the "cardio-metabolic" group (18.8%) and England the "respiratory-mental-articular" (5.1%). In the older group, Russia had the highest proportion of both classes (48.3% and 9%). Both the younger and older African participants presented the highest proportion of the "healthy" class. Older age, being woman, widowed, and with low levels of education and income were related to an increased risk of

For peer review only - http://bmjopen.bmj.com/site/about/guidelines.xhtml

multimorbidity. Physical activity was a protective factor in both age groups and smoking a risk factor for the “respiratory-mental-articular”.

Conclusions: Multimorbidity is common worldwide, especially in HICs and Russia. Health policies in each country addressing coordination and support are needed to face the complexity of a pattern of growing multimorbidity.

Keywords: multimorbidity, non-communicable diseases (NCDs), low- and middle- income countries (LMICs), high-income countries (HICs), latent class analysis (LCA).

For peer review only

Strengths and limitations of this study

- This study used a large, harmonized, multi-regional database, which allowed us to compare two age groups as well as disease prevalence in regions with differing incomes.
- The presence or absence of the non-communicable diseases was based on self-reported measures, and thus might be affected by measurement errors or lack of accuracy.
- Only common diseases across studies were included in the analyses, so this might have led to a smaller number of latent classes, or to different clusters.
- When performing Latent Class Analysis, we forced the solution as we aimed to do comparisons among age subsamples and regions in terms of disease prevalence as well as protective and risk factors.
- The use of multiple imputations for missing data in the covariates could carry some bias.

Background

By 2050, the population aged 60 years and older is expected to reach 2 billion worldwide, compared to 900 million in 2015 [1]. Along with this rapid increase, the incidence of chronic conditions (CC) or non-communicable diseases (NCDs) is also on the rise, having become the leading cause of morbidity and disability worldwide [2].

For peer review only - http://bmjopen.bmj.com/site/about/guidelines.xhtml

Multimorbidity, defined as the co-existence of two or more CC, is more common in older adults and is often more prevalent in people of lower socioeconomic status [3]. Multimorbidity is thought to account for 65% of total health care expenses in high-income countries (HICs) because of the huge associated healthcare utilization [4]. Due to the increasing prevalence of multimorbidity, the managing of multiple conditions has become an unavoidable international research priority, because of the high impact on the quality of life of patients and caregivers and on healthcare systems [3].

Most studies on the prevalence of multimorbidity in older people come from HICs, while data from middle-aged adults and low- and middle- income countries (LMICs) are much more limited [5–8]. LMICs are experiencing an increase in life expectancy that, together with changes in lifestyle and environment exposures, are triggering changes in their disease burden profile [3,9]. Few studies have compared patterns of multimorbidity between HICs and LMICs. Afshar et al. [10] used population-based chronic disease data from the World Health Survey to compare multimorbidity prevalence across 27 LMICs and one HIC, and used gross domestic product (GDP) to study inter-country socioeconomic differences. They found high multimorbidity prevalence in all countries, and a positive but non-linear relationship between country GDP and multimorbidity prevalence, suggesting the influence of other factors, such as lifestyles, social conditions, and differences across health systems. Four latent classes were identified in a cross-sectional sample of Australian seniors aged 50 years and over, using self-reported diagnosis of eleven conditions, including cancer and Parkinson's disease [11]. Another study, focusing on complex health care needs of Italian elderly people, found five clusters using 15 diseases [12]. A study conducted in a sample of 162,283 people from a survey of Danish population identified seven latent classes considering 15 chronic diseases and seven age groups, ranging from 16 to 104 years [13]. These differences could be explained in light of variations in collection methods, data sources, populations, diseases included, and the analysis performed [11,14,15].

Similarly, the lack of study of differences in multimorbidity between HICs and LMICs may be due to the use of different methodologies, which might hinder comparisons of prevalence and multimorbidity patterns across countries. The integration of data from different studies would allow us to determine

For peer review only - http://bmjopen.bmj.com/site/about/guidelines.xhtml

differences across regions and cohorts, as well as to explore risk and protective factors involved in the clustering of CC, thereby improving our understanding of the problem and the creation of adapted medical guidelines.

This study aimed to: a) identify multimorbidity clusters in middle-aged (50-64) and older adults (+65) from different regions, classified as LMICs and HICs; b) investigate the associations between multimorbidity clusters and sociodemographic, economic, lifestyles and health status variables; and c) explore differences across regions.

Methods

Study design and data extraction

The present study used data from the Ageing Trajectories of Health: Longitudinal Opportunities and Synergies (ATHLOS) project [16]. Longitudinal data from 17 international cohort studies related to health and ageing were harmonized with the aim of obtaining an integrated dataset and achieving a better understanding of ageing and health processes.

We selected three studies due to their inclusion of the variables of interest and the possibility of comparing HICs and LMICs. Baseline samples of the following studies were included in the analyses: the World Health Organization's Study on Global Ageing and Adult Health (SAGE) [17], the English Longitudinal Study of Ageing (ELSA)[18], and the Survey of Health, Ageing and Retirement in Europe Study (SHARE) [19]. These panel studies included non-institutionalized people aged 50 years and older. SAGE comprises six LMICs according to The World Bank Classification [20], namely Ghana, South Africa, Mexico, India, China and Russia; ELSA includes the English population and SHARE covers eleven countries of the European Union and Israel at baseline, considered as HICs [20].

The analyses presented focused on people aged 50 years of age and older who were part of the core sample of each study and who completed a non-proxy interview at baseline. We excluded from the analyses those participants who participated via proxy due to cognitive problems or severe physical

For peer review only - http://bmjopen.bmj.com/site/about/guidelines.xhtml

limitations. Moreover, people with missing values in sex and age were excluded, resulting in a final sample of 72 140 individuals. Mexico was excluded from the analyses due the high percentage of missingness in the variables of interest (See Additional file 1: Table S1).

Patient and public involvement

No patient involved.

Variables

The following variables were the result of a stringent, ex-post harmonization process using systematic harmonization methodology and tools from Maelstrom Research [21].

Eight NCDs were used to conduct the analysis, including those that were available in the three studies: diabetes, hypertension, asthma, chronic lung disease, joint disorders (arthritis, rheumatism or osteoarthritis), angina or myocardial infarction, stroke, and depression. The presence or absence of these conditions was self-reported and based on a medical diagnosis. Depression was assessed with standardized tools, such as the Composite International Diagnostic Interview (CIDI) in the SAGE study, the Center for Epidemiologic Studies Depression Scale (CES-D) in ELSA, and the EURO-D in SHARE [22–24]. A dichotomous variable (yes/no) was created using the indicated cut-off score for each tool and population based on previous studies [22,25,26].

Self-reported demographic variables included age, sex, level of education (primary or less, secondary, and tertiary), marital status (single, married or currently cohabiting, separated or divorced, and widowed) and quintiles of household wealth (first quintile indicating lowest level). Life-styles and health behaviors were 'ever smoked' any type of tobacco and physical activity referring to the practice of vigorous exercise during the last two weeks, both coded as *yes* or *no*. Other health-related variables were self-rated health (good, moderate, or poor), presence or absence of loneliness feelings in the last week, difficulties in activities of daily living (ADL), cognitive performance, and number of diseases.

For peer review only - http://bmjopen.bmj.com/site/about/guidelines.xhtml

To assess difficulties in ADL, we used a set of daily self-care activities, which were common across studies, such as problems in using the toilet, bathing or showering, getting dressed, eating, moving, or getting in or out of bed. Each of the ADL difficulties was coded into a *yes/no* if the person answered 'severe' or 'extreme/cannot do it'. To build the set of ADL difficulties, we coded *yes* if the person reported at least one difficulty in any of the six items.

Immediate and delayed recall was assessed using the 10-word learning list task, and verbal fluency utilizing the animal naming test [27]. Continuous total scores were used to perform the analyses. Number of diseases was built by adding up the occurrences of all the above-mentioned NCDs.

Finally, a 7-level regional membership variable was created in order to analyze regional differences, based on the World Health Organization (WHO) and the United Nations Statistical Division (UNSD) regional classification [28,29]. Moreover, the World Bank Classification was used to classify these regions into HICs or LMICs [20]. SAGE includes Africa (Ghana and South Africa), China and India, all of them considered as LMICs. SHARE countries were grouped into three regions: Northern Europe (Denmark, Sweden), Southern Europe (Greece, Italy, and Spain) and Western Europe (Austria, Belgium, France, Germany, Israel, Netherlands, and Switzerland). ELSA and SHARE regions were considered as HICs. Ghana and South Africa were grouped together and named as Africa for practical purposes as well as due to their smaller sample size. These countries are not necessarily representative of the whole continent.

Statistical analysis

All the analyses were performed using data from the baseline. Descriptive statistics were used to summarize information regarding sociodemographic economic variables and disease prevalence among regions. Confidence intervals (95%IC) were calculated for categorical variables in order to make comparisons across regions.

For peer review only - http://bmjopen.bmj.com/site/about/guidelines.xhtml

For peer review only

Latent Class Analysis (LCA) was conducted stratified by age (50-64, +65). Eight NCDs (diabetes, hypertension, asthma, chronic lung disease, joint disorders, angina-myocardial infarction, stroke, and depression) were used as observed indicators without using covariates since we aimed to identify latent classes only based on disease variables. Region was used as cluster when conducting LCA in order to accurately describe disease proportions, indicating that the subjects were not independent random draws, but rather were nested within clusters [30] .

The optimal number of latent classes was determined using the adjusted Bayesian Information Criterion (aBIC), the consistent Akaike Information Criterion (CAIC), and the Entropy Index. Lower values of aBIC and CAIC indicate better fit, whereas entropy index values higher than 0.80 indicate that the latent classes are highly discriminating [31]. The average posterior probability indicates how well a model classifies individuals into their most likely class. Values higher than 0.70 indicate well-identified classes [32]. Additionally, interpretability and clinical judgment were used [32,33].

Missing data in one of the indicators was handled with the full-information maximum likelihood (FIML) technique, assuming missing-at-random (MAR) [34]. Missing data in the covariates was handled using multiple imputation by chained equations (MICE) assuming MAR [34]. The imputation model included the outcome (group membership in one of the latent classes) and all the variables used in the regression models. In the Additional file 1: Table S2-10 there is a report of those variables and the percentage of missingness of each region in the variables of interest.

Adjusted multinomial logistic regression models were used to assess the association between the outcome (multimorbidity classes, with the "Healthy" class as the reference category) and several variables: loneliness, ever smoked, physical activity, limitations in ADL, self-rated health, immediate recall, delayed recall and verbal fluency. The model was additionally adjusted for sex, age, marital status, education level, wealth, and the region at baseline. Due to potential collinearity between income and education, we checked the significance and magnitude of the correlation between both variables. The association was small, and thus both covariates were included as separate variables in

For peer review only - http://bmjopen.bmj.com/site/about/guidelines.xhtml

the models. Regression models were conducted separately in one hundred imputed datasets and results combined using Rubin's rules [35].

All analyses were conducted with Stata SE version 13.1 (College Station, TX). LCA analyses were performed using a Stata plugin [30].

Results

Descriptive analysis

In Table 1 are presented the main characteristics of the sample by region. The mean age ranged from 62 (SD=9.02) in Southern Asia to 65 years (10.18) in Russia and (10.26) in England. Some 54% were women, 72% were married or cohabitating, and 39% had secondary education. Russia presented the highest number of conditions (mean 1.66) compared to Africa (0.64), China (0.80), and India (0.72).

The most prevalent conditions in the total sample were hypertension (31.2%, 95%CI = 30.9-31.6) and joint disorders (22.4%, 95%CI = 22.0-22.7). Hypertension was particularly high in Russia (56.5%, 95%CI = 54.9-58.1) compared to the other regions. Diabetes prevalence was greater in Southern (11.9%, 95%CI = 11.2-12.7) and Western Europe (10.4%, 95%CI = 9.9-10.9), whereas Africa and China presented the lowest proportions. Similarly, myocardial infarction–angina was highly prevalent in Russia (33.1%, 95%CI = 31.6-34.6), followed by countries of Northern (13.8%, 95%CI = 12.8-14.8), Southern (11.7%, 95%CI = 11.0-12.4) and Western Europe (13.1%, 95%CI =12.6-13.6).

For peer review only

Table 1 Main characteristics of the total sample and by regions

**Region**	Total	Africa[a]	China[b]	India[b]	Russia[b]	England[c]	Northern Europe[d]	Southern Europe[e]	Western Europe[f]
	N=72140	n=7950	n=12840	n=6558	n=3887	n=11 517	n=4573	n=7465	n=17 350
**Age, mean (SD)**	64.05 (9.96)	63.60 (10.26)	63.07 (9.31)	61.80 (9.02)	65.06 (10.18)	65.06 (10.26)	64.78 (10.34)	65.02 (10.17)	64.34 (9.96)
**Woman, % (95% CI)**	54.0 (53.6-54.4)	52.3 (51.2-53.4)	52.9 (52.1-54.8)	49.6 (48.4-50.8)	64.6 (63.0-66.1)	54.6 (53.7-55.5)	53.2 (51.8-54.7)	55.4 (54.2-56.5)	54.1 (53.4-54.9)
**Marital status, % (95% CI)**									
Single	4.1 (4.0-4.3)	6.7 (6.2-7.3)	0.9 (0.7-1.6)	1.0 (0.8-1.2)	2.8 (2.3-3.4)	5.0 (4.6-5.4)	5.5 (4.8-6.2)	6.3 (5.8-6.9)	4.9 (4.6-5.3)
Married	71.5 (71.1-72.0)	54.5 (53.4-55.6)	83.5 (82.8-84.1)	74.1 (73.1-75.2)	56.1 (54.5-57.7)	69.1 (68.2-69.9)	72.4 (71.0-73.7)	73.3 (72.3-74.3)	73.3 (72.7-74.0)
Divorced	5.9 (5.7-6.2)	10.4 (9.7-11.1)	1.7 (1.5-2.0)	0.6 (0.5-0.9)	8.3 (7.4-9.2)	9.0 (8.5-9.5)	9.6 (8.8-10.5)	2.6 (2.3-3.0)	6.8 (6.5-7.2)
Widowed	18.4 (18.1-18.6)	27.3 (26.3-28.3)	13.9 (13.3-14.6)	24.3 (23.2-25.3)	32.7 (31.2-34.2)	16.9 (16.3-17.6)	12.5 (11.6-13.5)	17.8 (16.9-18.6)	14.9 (14.4-15.4)
**Education level % (95% CI)**									
Primary or less	33.4 (33.0-33.7)	30.5 (29.5-31.5)	37.9 (37.1-38.8)	25.5 (24.5-26.6)	9.9 (9.0-10.9)	42.4 (41.5-43.4)	29.6 (28.3-30.9)	59.4 (58.3-60.5)	23.2 (22.6-23.8)
Secondary	38.7 (38.4-39.1)	19.0 (18.2-19.9)	33.2 (32.4-34.0)	18.2 (17.3-19.2)	69.3 (67.9-70.8)	37.5 (36.7-38.4)	45.1 (43.6-46.5)	31.3 (30.2-32.3)	55.1 (54.3-55.8)
Tertiary	12.0 (11.9-12.2)	3.9 (3.5-4.3)	4.7 (4.3-5.0)	5.0 (4.4-5.5)	19.7 (18.5-21.0)	11.1 (10.6-11.7)	24.2 (23.0-25.5)	9.1 (8.4-9.7)	20.8 (20.2-21.4)
**Wealth (quintiles% (95% CI)**									
1st (worse)	19.3 (19.1-19.6)	19.3 (18.4-20.2)	19.8 (19.1-20.5)	16.2 (15.3-17.1)	18.2 (17.0-19.5)	19.0 (18.3-19.7)	20.7 (19.6-21.9)	20.3 (19.4-21.2)	19.9 (19.3-20.5)
2nd	19.7 (19.4-20.0)	19.7 (18.4-20.2)	19.7 (19.0-20.4)	18.6 (17.6-19.5)	19.8 (18.5-21.1)	19.3 (18.6-20.1)	20.4 (19.3-21.6)	20.4 (19.5-21.3)	19.9 (19.4-20.6)
3rd	19.7 (19.4-19.9)	19.8 (18.9-20.7)	20.1 (19.4-20.8)	18.4 (17.5-19.4)	20.3 (19.0-21.6)	19.7 (18.9-20.4)	20.1 (19.0-21.3)	19.8 (18.9-20.7)	19.5 (18.9-20.1)
4th	19.9 (19.7-20.3)	20.5 (19.6-21.4)	20.5 (19.8-21.2)	21.5 (20.5-22.5)	20.0 (18.8-21.3)	19.6 (18.9-20.3)	19.7 (18.5-20.8)	19.4 (18.6-20.4)	19.3 (18.8-19.9)
5th (best)	20.1 (19.8-20.4)	20.4 (19.5-21.3)	19.7 (19.0-20.4)	24.8 (23.8-25.9)	21.6 (20.3-22.9)	19.6 (18.9-20.3)	18.9 (17.8-20.1)	18.9 (18.0-19.8)	19.4 (18.8-20.0)
**Nº diseases, *mean (SD)***	1.02 (1.14)	0.64 (0.94)	0.80 (0.99)	0.72 (0.97)	1.66 (1.38)	1.19 (1.13)	1.02 (1.10)	1.28 (1.25)	1.10 (1.16)
**Diseases, % (95% CI)**									
Diabetes	8.5 (8.3-8.7)	6.6 (6.1-7.2)	6.5 (6.1-7.0)	7.3 (6.7-7.9)	9.0 (8.1-10.0)	7.4 (6.9-7.9)	8.2 (7.4-9.0)	11.9 (11.2-12.7)	10.4 (9.9-10.9)
Hypertension	31.2 (30.9-31.6)	21.5 (20.6-22.4)	27.4 (26.6-28.2)	17.5 (16.6-18.5)	56.5 (54.9-58.1)	37.8 (36.9-38.7)	29.3 (28.0-30.7)	35.6 (34.5-36.7)	32.3 (31.6-33.0)
Joint disorders	22.4 (22.0-22.7)	17.8 (16.9-18.6)	22.1 (21.4-22.8)	17.9 (17.0-18.9)	35.2 (33.7-36.7)	32.5 (31.6-33.3)	15.7 (14.6-16.8)	26.3 (25.3-27.3)	16.8 (16.3-17.4)
Asthma	5.5 (5.3-5.6)	4.1 (3.7-4.5)	2.4 (2.2-2.7)	6.9 (6.3-7.6)	3.4 (2.9-4.0)	11.7 (11.1-12.3)	7.6 (6.8-8.4)	4.1 (3.7-4.6)	4.1 (3.8-4.4)
Chronic lung disease	6.1 (5.9-6.3)	1.5 (1.2-1.7)	8.6 (8.1-9.1)	4.1 (3.6-4.6)	17.9 (16.8-19.2)	6.5 (6.1-7.0)	4.5 (3.9-5.2)	5.4 (4.9-6.0)	4.9 (4.6-5.3)
MI - Angina	10.0 (9.8-10.3)	4.5 (4.1-5.0)	8.8 (8.3-9.3)	4.9 (4.4-5.5)	33.1 (31.6-34.6)	3.3 (3.0-3.6)	13.8 (12.8-14.8)	11.7 (11.0-12.4)	13.1 (12.6-13.6)
Stroke	3.8 (3.7-3.9)	3.2 (2.8-3.6)	3.5 (3.2-3.8)	2.2 (1.9-2.6)	6.0 (5.3-6.8)	4.5 (4.1-4.9)	5.0 (4.4-5.7)	3.1 (2.8-3.6)	3.9 (3.7-4.2)
Depression	15.3 (15.0-15.5)	5.8 (5.3-6.3)	1.2 (1.1-1.4)	12.1 (11.3-12.9)	5.2 (4.5-5.9)	16.5 (15.8-17.2)	18.8 (17.7-20.0)	31.7 (30.6-32.7)	25.0 (24.3-25.6)

Note. [a]SAGE study - Africa: Ghana, South Africa; [b]SAGE study; [c]ELSA study- England; [d]SHARE study - Northern Europe: Denmark, Sweden; [e]SHARE study - Southern Europe: Greece, Italy, Spain; [f]SHARE study - Western Europe: Austria, Belgium, France, Germany, Israel, Netherlands, Switzerland.

The analyses were performed before multiple imputation procedure

For peer review only

For peer review only - http://bmjopen.bmj.com/site/about/guidelines.xhtml

Joint disorders were more prevalent in Russia (35.2%, 95%CI = 33.7-36.7) and England (32.5%, 95%CI =31.6-33.3). The prevalence of asthma was greater in England than other regions (11.7%, 95%CI = 11.1-12.3) and chronic lung disease was greater in Russia (17.9%, 95%CI = 16.8-19.2).

As for the prevalence of depression, European countries presented the highest values, especially in Southern (31.7%, 95%CI = 30.6-32.7) and Western Europe (25.0%, 95%CI = 24.3-25.6), whereas LMICs showed very low proportions, especially in China, where only 1.2% of people aged 50+ presented depression.

Multimorbidity patterns

Table 2 displays the aBIC, CAIC, and entropy values, proportions, and average posterior probability of each latent class, for a two- to five-class model in both age subsamples. In the younger subsample (50-64), the five-class solution yielded the lowest aBIC and CAIC values and the highest entropy value (0.67). However, it was dismissed because one of the latent classes was very infrequent and the posterior probabilities were far below 0.70. Similarly, the four-class model was rejected for an inadequate posterior probability value in one of the classes (0.52). The model finally selected was the three-class model. The three-class solution was also chosen for the older age group because of lower posterior probability values in the four- and five-class models in spite of lower aBIC and CAIC values.

We named each latent class according to the most prevalent diseases within each latent class. Figure 1 shows the distribution of each condition across the three latent classes (“cardio-metabolic”, “respiratory-mental-articular”, and “healthy” class) in the total sample and by regions. The “cardio-metabolic” class presented excess prevalence of diabetes, hypertension, myocardial infarction or angina, and stroke, comprising 8.93% of the total sample in the younger group and 27.22% in the older group. The “respiratory-mental-articular” class, which comprised 3.91% and 5.27% of each sample, respectively, showed greater prevalence of joint disorders, asthma, chronic lung diseases, and

For peer review only - http://bmjopen.bmj.com/site/about/guidelines.xhtml

For peer review only

depression. Finally, the “healthy” class presented low prevalence of conditions, comprising 87.16% of the sample in the first age group and 67.51% in the second group.

For peer review only

For peer review only - http://bmjopen.bmj.com/site/about/guidelines.xhtml

Table 2 Comparison between models in individuals aged 50-64 and +65

	Aged 50-64					Aged ≥65				
No. of latent classes	Information Criteria Indices		Classification Quality	Latent classes, n (%)	Average posterior probability	Information Criteria Indices		Classification Quality	Latent classes, n (%)	Average posterior probability
	aBIC	CAIC	Entropy			aBIC	CAIC	Entropy		
2	1512.91	1583.94	0.51	33023 (82.15)<br>7177 (17.85)	.88<br>.75	1603.30	1674.33	0.39	21113 (66.10)<br>10827 (33.90)	.83<br>.75
**3**	**875.23**	**983.86**	**0.63**	**3589 (8.93)**<br>**1571 (3.91)**<br>**35040 (87.16)**	**.76**<br>**.67**<br>**.87**	**1032.83**	**1141.46**	**0.50**	**8693 (27.22)**<br>**1684 (5.27)**<br>**21563 (67.51)**	**.71**<br>**.68**<br>**.81**
4	777.14	923.37	0.43	25701 (63.93)<br>626 (1.56)<br>7754 (19.29)<br>6119 (15.22)	.52<br>.72<br>.80<br>.68	817.60	963.83	0.63	9557 (29.92)<br>1474 (4.61)<br>17220 (53.91)<br>3689 (11.55)	.65<br>.78<br>.86<br>.77
5	661.04	844.87	0.67	4578 (11.39)<br>247 (0.61)<br>32423 (80.65)<br>1359 (3.38)<br>1593 (3.96)	.64<br>.67<br>.85<br>.48<br>.64	689.22	873.05	0.59	11094 (34.73)<br>1094 (3.43)<br>14155 (44.32)<br>1148 (3.59)<br>4449 (13.93)	.77<br>.71<br>.76<br>.63<br>.72

Note: Boldface indicates the final selected model. *aBIC* adjusted Bayesian Information Criterion, *CAIC* Consistent Akaike Information Criterion

For peer review only

Differences in the proportions of multimorbidity classes were found across regions (Figure 1). The "cardio-metabolic" class (18.8%, 95%CI =17.1-20.6) was significantly greater in Russia than in other regions, and England (5.1%, 95%CI =4.5-5.7) showed a higher proportion of individuals classified into the "respiratory-mental-articular" class. The "healthy" class was higher in Africa (91.5%, 95%CI = 90.7-92.3), China (90.8%, 95%CI = 90.1-91.4), and India (89.5%, 95%CI = 88.5-90.4), and remarkably lower in Russia (71.6%, 95%CI = 69.5-73.6) compared to other regions.

----- FIGURE 1 HERE -----

Similar results were found for the older group (Figure 2). In Russia, the "cardio-metabolic" class was significantly higher than in other regions (48.3%, 95%CI = 46.1-50.6) whereas the "healthy" class was the least frequent class compared with the rest of regions (38.4%, 95%CI = 36.2-40.6), followed by Southern Europe (52.6%, 95%CI = 50.9-54.2). Africa and India showed lower proportions of individuals classified into the "cardio-metabolic" class (12.9%, 95%CI = 11.8-14.1 and 11.2%, 95%CI = 10.0-12.6, respectively).

----- FIGURE 2 HERE -----

Association between multimorbidity classes and covariates

In the Additional file 2: Table S1 are presented the unadjusted relative risk ratios (RRR) for both age subsamples. The "healthy" class was used as the reference group. In the case of the younger subsample, and compared with the "healthy" class, individuals classified into the "cardio-metabolic" and "respiratory-mental-articular" classes were more likely to be older (RRR = 1.09, 95%CI = 1.08-1.10; RRR = 1.06, 95%CI = 1.04-1.07, respectively) and being widowed (RRR = 1.4, 95%CI = 1.1-1.7) and divorced in the "respiratory-mental-articular" class (RRR = 1.7, 95%CI = 1.2-2.4). Being a man, having tertiary education, and high levels of wealth had a protective effect for being in both multimorbidity groups, compared with the "healthy" class. Similarly, those individuals from the older subsample who

For peer review only - http://bmjopen.bmj.com/site/about/guidelines.xhtml

For peer review only

were in the fourth and fifth quintile (RRR = 0.8, 95%CI = 0.7-1.0) were less likely to be classified into the “respiratory-mental-articular” class, compared with the “healthy” group.

Regarding the association of regions and multimorbidity groups, some differences were found in the younger subsample. Taking Africa as the reference category, participants from Russia were more likely to be classified into the “cardio-metabolic” class (RRR = 3.6, 95%CI = 3.0-4.2), whereas individuals from England (RRR = 5.6, 95%CI = 4.1-7.7), Northern Europe (RRR = 2.8, 95%CI = 1.9-4.1) and India (RRR = 2.2, 95%CI = 1.5-3.2) showed higher risk of being in the “respiratory-mental-articular” class. In the case of the older subsample, all regions had greater risk of being classified into the “respiratory-mental-articular” class, compared to the “healthy” class, especially participants from Russia (RRR = 14.5, 95%CI = 10.3-20.3), compared to Africa.

Table 3 shows the adjusted relative risk ratios (RRR) for both age subsamples, taking the “healthy” class as the reference group. Both multimorbidity classes (cardio-metabolic and respiratory-mental-articular) were associated with all the covariates in the younger group, except for smoking status in the “cardio-metabolic” class.

In the younger individuals subsample, both latent classes were more likely to be associated with the presence of feelings of loneliness (RRR = 1.8, 95%CI = 1.7-2.0; RRR = 2.5, 95%CI = 2.0-3.0), limitations in ADL (RRR = 3.2, 95%CI = 2.9-3.6; RRR = 3.9, 95%CI = 3.3-4.7) and worse health status (RRR = 12.8, 95%CI = 11.3-14.4; RRR = 12.9, 95%CI = 10.5-16.0). Physical activity had a protective effect for being in these classes and having smoked was a risk factor only for being classified into the “respiratory-mental-articular” class (RRR = 1.5, 95%CI = 1.2-1.7). Conversely, those older individuals who had ever smoked had a higher risk of being in the “respiratory-mental-articular” group (RRR = 1.8, 95%CI = 1.5-2.0) and a lower risk of being classified into the “cardio-metabolic” class (RRR = 1.0, 95%CI = 0.9-1.0). For both age subsamples, better performance in verbal memory was significantly associated with less risk of

For peer review only

For peer review only - http://bmjopen.bmj.com/site/about/guidelines.xhtml

being classified into the two multimorbidity classes. Similarly, higher scores in verbal fluency were a protective factor for multimorbidity, compared with the healthy individuals group.

Table 3 Association between latent multimorbidity membership and outcomes in individuals aged 50-64 and ≥65

Variables[a]	Aged 50-64		Aged ≥65	
	"Cardio-metabolic" class	"Respiratory-mental-articular" class	"Cardio-metabolic" class	"Respiratory-mental-articular" class
Loneliness (yes/no)	1.8 (1.7-2.0)	2.5 (2.0-3.0)	1.3 (1.2-1.5)	1.9 (1.7-2.3)
Ever smoked (yes/no)	1.1 (1.0-1.2)	1.5 (1.2-1.7)	1.0 (0.9-1.0)	1.8 (1.5-2.0)
Physical activity (yes/no)	0.4 (0.4-0.5)	0.5 (0.5-0.6)	0.5 (0.5-0.6)	0.4 (0.4-0.5)
Limitations in ADL (yes/no)	3.2 (2.9-3.6)	3.9 (3.3-4.7)	2.3 (2.1-2.5)	4.0 (3.5-4.6)
Self-rated health				
Good	1.0	1.0	1.0	1.0
Moderate	4.8 (4.3-5.2)	3.9 (3.2-4.7)	3.1 (2.9-3.4)	5.7 (4.8-6.8)
Poor	12.8 (11.3-14.4)	12.9 (10.5-16.0)	6.2 (5.6-6.9)	19.4 (16.2-23.4)
Memory: Immediate recall	0.9 (0.9-0.9)	0.9 (0.9-1.0)	0.9 (0.9-0.9)	0.9 (0.9-1.0)
Memory: Delayed recall	0.9 (0.9-0.9)	0.9 (0.9-1.0)	0.9 (0.9-0.9)	0.9 (0.9-1.0)
Verbal fluency	1.0 (1.0-1.0)	1.0 (1.0-1.0)	0.9 (0.9-1.0)	1.0 (1.0-1.0)

Note: The reference group for the multimorbidity group variable was the "Healthy" class
Relative Risk Ratios (95% confidence interval) from multinomial logistic regression models
Models were run in 100 imputed datasets and results combined using Rubin's rules
ADL Activities of Daily Living
[a]Adjusted for sex, age, marital status, education level, wealth and region

Discussion

To the best of our knowledge, this is the first multi-region study to use harmonized data to compare multimorbidity patterns across different regions from three distinct population-based cohorts. We identified three latent classes of multimorbidity based on the presence of eight NCDs: the "cardio-metabolic", the "respiratory-mental-articular" and the "healthy" class. The same clusters were identified in another study using SAGE original data, applying exploratory factor analysis (EFA) in a sample of 41 909 individuals aged 50 years or older [36]. Similarly, a study of a representative sample of Spanish community-dwelling adults over 50 years old also found three latent classes using eleven chronic conditions, showing similar diseases distributions among the multimorbidity clusters [37].

In our study, for both age groups the majority of the sample was classified into the "healthy" class, 87.16% and 67.51%, respectively. This latent group has previously been described in studies which

For peer review only - http://bmjopen.bmj.com/site/about/guidelines.xhtml

applied LCA [11,13,37,38]. Likewise, the other two identified classes are similar to those reported in a systematic review based on 14 studies of multimorbidity patterns [39]. In this review, the most prevalent diseases in the “cardio-metabolic” group were diabetes, hypertension, heart diseases, hyperlipidemia, and obesity; and in the second group conditions such as mental disorders, thyroid disease, neurological disease, pain, asthma or chronic lung diseases, musculoskeletal disorders, obesity, and gastroesophageal reflux disease were included. Despite the fact that we included a smaller number of diseases, we found analogous patterns. In our study, 8.93% of the younger group (50-64) and 27.22% of the older were classified into the “cardio-metabolic” class, including individuals with higher prevalence of diabetes, hypertension, myocardial infarction or angina, and stroke. This clustering of diseases is similar to the metabolic syndrome, which has metabolically-related cardiovascular risk factors and greater risk of stroke and diabetes [40]. Lastly, the least prevalent group was the “respiratory-mental-articular” class, consisting of greater prevalence of joint disorders, asthma, chronic lung diseases, and depression. Association between depression and arthritis has commonly been reported, with socioeconomic and disease factors reported as being involved in its association, as well as systemic inflammation mechanisms [3]. Nevertheless, the links between depression and chronic lung diseases, and chronic lung diseases and arthritis, despite having been studied, remain unclear [40,41].

Analogous latent multimorbidity classes have been found among both age groups. Despite this, certain aspects should be pointed out. As expected, the proportion of participants classified into the “healthy” class was greater in participants aged 50-64 (87.16%) compared to those aged +65 years old (67.51%), illustrating higher multimorbidity in elderly individuals. The distribution of CC was also less clear in the older subsample. For example, both joint disorders and angina–myocardial infarction were similarly present in the “cardio-metabolic” and “respiratory-mental-articular” categories, whereas in the younger participants (50-64) subsample we observed a more differentiated profile of those chronic conditions that cluster into one latent class. For example, respiratory-related diseases (asthma, chronic

For peer review only - http://bmjopen.bmj.com/site/about/guidelines.xhtml

lung diseases) are highly presented in the “respiratory-mental-articular” class, while very infrequent among middle-aged people classified into the “cardio-metabolic” group. It is worth mentioning that although depression is frequently observed among participants classified into the “respiratory-mental-articular” class, it is not infrequent among people within the “cardio-vascular” class. This may be due to the relationship between mental and physical disorders, which has frequently been reported, suggesting a bidirectional association between them [42]. On the one hand, medical conditions could be accompanied by a high symptom burden, leading to depression, and, on the other, depression could be a risk factor for medical conditions, since depressive symptoms could increase the incidence of behaviors, such as smoking, alcohol intake, poor diet, or physical inactivity, which are risk factors for NCDs [3,42].

One important implication of our findings is the relatively high proportion of people aged 50-64 with multimorbidity. Thus, preventive and intervention programs are also needed for this population to mitigate the multimorbidity burden.

Our results show that these multimorbidity patterns are qualitatively different, but only if compared to the “healthy” class in terms of sociodemographic and economic characteristics, lifestyles, and health status variables. As has been reported in the literature, being older, woman, widowed, with a lower level of education and lower socioeconomic status are related to an increased risk of multimorbidity [3]. In addition, those individuals with multiple chronic conditions were more likely to have limitations in ADL, especially those classified into the “respiratory-mental-articular” group, similar to what was found in another study of multimorbidity [37]. Physical activity seems to be a protective factor for being classified into the “respiratory-mental-articular” class, whereas smokers were more likely to be classified into the “respiratory-mental-articular” class, but not the “cardio-metabolic” class. This is inconsistent with the literature, since cigarette smoking is considered a major cause of cardiovascular diseases (CVDs). However, smoking is probably the most complex and least understood risk factor for CVDs [43].

For peer review only - http://bmjopen.bmj.com/site/about/guidelines.xhtml

One interesting finding is the association between cognition outcomes and multimorbidity in both age subsamples. Better performance in verbal memory and fluency was related to less risk of being classified into the multimorbidity groups, with similar results among latent classes. Impaired cognition has been associated with conditions such as arthritis [44], depression [45], and respiratory diseases [46], cardiovascular conditions, diabetes [47], hypertension [48], and coronary heart diseases [49].

Concerning the regional distribution of multimorbidity, Russia accounted for the highest burden as opposed to Africa, China, and India. The "cardio-metabolic" class is especially common in this country, with a prevalence of 18.82% in the younger and 48.34% in the older subsample. Prevalence of CVDs, such as hypertension, myocardial infarction or angina, and stroke, was also higher in Russia. This high proportion could be related to the high rate of alcohol consumption and rapid societal changes experienced in this country which might account for increased risk of circulatory diseases [50,51]. Followed by Russia, European regions showed higher rates of multimorbidity. NCDs such as hypertension, joint disorders, respiratory diseases, and depression were highly prevalent, especially in England and Southern Europe, where the "respiratory-mental-articular" class was highly prevalent in both age subsamples. The relationship between mood disorders such as depression and joint disorders has been previously reported in other studies, though the underlying cause remains unclear [36,37,39]. Notwithstanding, previous studies suggested that the emotional burden of joint disorders may contribute to the onset of psychiatric disorders [36,52].

LMICs such as Africa, China, and India showed lower rates of multimorbidity compared to Russia and other HICs. However, there was a wide variation in terms of some diseases, such as respiratory diseases and depression. Asthma and chronic lung diseases were highly prevalent in India and China, influenced by factors such as increasing smoking rates, air pollution, and occupational lung diseases in these countries [53]. As reported in previous studies [54], depression was remarkably prevalent in India, whereas the lowest prevalence was observed in China. This is in line with previous epidemiological studies on the prevalence of depression in Chinese older people, suggesting differences in diagnostic

For peer review only - http://bmjopen.bmj.com/site/about/guidelines.xhtml

criteria that make depression less diagnosed; somatic symptoms are more prevalent in this population instead of sadness, and lack of interest and energy. Moreover, stigma and prejudice in Chinese population might also contribute to under-reporting depressive symptoms [55,56]. Furthermore, the variation found across regions in terms of depression prevalence could be due to cultural differences in expressions or expectations of mood disorders or mental health [57].

The highest burden of multimorbidity in HICs could be explained by an increased level of development in the HICs. Notwithstanding, LMICs are experiencing a change in lifestyle and environmental exposures which contributes, as in HICs, to multimorbidity. Thus, the increased burden of NCDs, in addition to the existing burden of infectious diseases such as HIV/AIDS, worsens multimorbidity management [3] . Moreover, the differences found in the regional distribution of multimorbidity might be linked to different stages of development of their health systems, since there are differences between LMICs and HICs in terms of opportunities and barriers to improving the organization, integration, and delivery of multimorbidity care [3].

Strengths and limitations

A major strength of this study is the use of a large, harmonized, multi-regional database. Research on multimorbidity has typically been hampered by several factors, such as the exclusion of patients with multimorbidity from participation, targeting of research mostly on elderly individuals, and a shortage of studies focusing on LMICs. The ATHLOS study allowed us to compare two age groups (50-64 and 65 or older) as well as disease prevalence and clusters of conditions in regions with differing incomes in a very large, diverse population-based study of middle-aged and older adults.

Some limitations should be considered when interpreting our findings. Firstly, the presence or absence of the NCDs was based on self-reported measures, and thus might be affected by measurement errors or lack of accuracy. Nevertheless, some authors sustain self-reported diagnostics as a well-established method for the measurement of multimorbidity in population-based studies [58]. Secondly,

For peer review only - http://bmjopen.bmj.com/site/about/guidelines.xhtml

participants with an incipient neurodegenerative disease may have been included in our analytical sample. However, we excluded those participants who completed a proxy interview due to cognitive problems, such as neurodegenerative diseases, which could affect the reliability of the data. Nonetheless, participants with an incipient neurodegenerative disease may have been included in our analytical sample because of the lack of strong diagnostic criteria for dementia in the included studies. Thirdly, we could only focus on those diseases that were common across studies. Conditions such as obesity, cancer, kidney disease, and neurological illness were not evaluated. This might have led to a smaller number of latent classes, or to different patterns of multimorbidity. Fourthly, when performing LCA, the three-class solution was forced. In order to determine whether the latent classes were equivalent, invariance analysis should have been performed [59]. Nevertheless, this solution was forced as we aimed to do comparisons among age subsamples and regions in terms of disease prevalence as well as protective and risk factors. Finally, the use of multiple imputations could carry some bias. Despite this, the use of multiple imputation procedures is widely advocated when missing data occur in one or more covariates in a regression model and under a MAR assumption [60,61].

The results of this study suggest that NCDs cluster together in non-random associations across several regions worldwide. The three qualitatively distinct entities are also linked to several sociodemographic and economic characteristics, lifestyles, and health status variables. A deeper understanding of the interactions across regions and the studied variables is needed. Knowledge regarding broad patterns of conditions may contribute to the creation and implementation of guidelines that consider clusters of conditions instead of single diseases, since multimorbidity has become an unavoidable reality. Future efforts should focus on the underlying mechanisms of these clusters as well as their stability over time using longitudinal data. Moreover, cohort and age effects should be explored as might influence the likelihood of reporting some diagnosis and hence result in different multimorbidity patterns.

For peer review only - http://bmjopen.bmj.com/site/about/guidelines.xhtml

Acknowledgments

- The English Longitudinal Study of Ageing (ELSA):

ELSA is supported by the U.S. National Institute of Aging, the National Centre for Social Research, the University College London (UCL), and the Institute for Fiscal Studies. The authors gratefully acknowledge the UK Data Service and UCL who provided data for this paper.

- The Study on Global Ageing and Adult Health (SAGE):

The SAGE study is funded by the U.S. National Institute on Aging and has received financial support through Interagency Agreements (OGHA 04034785; YA1323-08-CN-0020; Y1-AG-1005-01) and Grants (R01-AG034479; IR21-AG034263-0182). The authors gratefully acknowledge the World Health Organization who provided data for this paper.

- The Survey of Health, Ageing and Retirement in Europe (SHARE):

The SHARE study is funded by the European Commission through FP5 (QLK6-CT-2001-00360), FP6 (SHARE-I3: RII-CT-2006-062193, COMPARE: CIT5-CT-2005-028857, SHARELIFE: CIT4-CT-2006-028812) and FP7 (SHARE-PREP: N°211909, SHARE-LEAP: N°227822, SHARE M4: N°261982). Additional funding from the German Ministry of Education and Research, the Max Planck Society for the Advancement of Science, the U.S. National Institute on Aging (U01_AG09740-13S2, P01_AG005842, P01_AG08291, P30_AG12815, R21_AG025169, Y1-AG-4553-01, IAG_BSR06-11, OGHA_04-064, HHSN271201300071C) and from various national funding sources is gratefully acknowledged (see www.share-project.org).

Funding: This work was supported by the five-year Ageing Trajectories of Health: Longitudinal Opportunities and Synergies (ATHLOS) project and the Centro de Investigación Biomédica en Red de Salud Mental (CIBERSAM). The ATHLOS project has received funding from the European Union's

For peer review only - http://bmjopen.bmj.com/site/about/guidelines.xhtml

Horizon 2020 research and innovation program under grant agreement No 635316. DF's work has been supported by grant RTI2018-100927-J-I00 Retos Investigación from Ministerio de Ciencia e Innovación (MCI), by Marsden grant E2987-3648 (Royal Society of New Zealand), and by grant 2017 SGR 622 (GRBIO) from the Departament d'Economia i Coneixement de la Generalitat de Catalunya (Spain). This work, grant number RTI2018-100927-J-I00, is supported by the Ministerio de Ciencia e Innovación (MCI, Spain), by the Agencia Estatal de Investigación (AEI, Spain) and by the European Regional Development Fund FEDER (FEDER, UE). BO's work is supported by the PERIS program 2016-2020 "Ajuts per a la Incorporació de Científics i Tecnòlegs" [grant number SLT006/17/00066], with the support of the Health Department from the Generalitat de Catalunya.

Competing interests: The authors report no conflicts of interest.

Patient consent for publication: not required

Ethics approval: The study protocol was approved by the Committee on the Ethics of Clinical Research, CEIC Fundació Sant Joan de Déu (Protocol No: PIC-22-15). All data were anonymized and EHR confidentially was respected in accordance with national and international law.

Data sharing statement: The original studies data are available on their respective websites: the Study on Global Ageing and Adult Health - SAGE (https://www.who.int/healthinfo/sage/en/), the English Longitudinal Study of Ageing – ELSA (https://www.elsa-project.ac.uk/), and the Survey of Health, Ageing and Retirement in Europe – SHARE (http://www.share-project.org/home0.html). R codes for harmonizing the included variables, as well as the STATA codes for the performed analysis are available on https://github.com/athlosproject/athlos-project.github.io

Additional files:

Additional file 1: **Table S1.** Proportion of missingness in the variables of interest in Mexico; **Table S2.** Overall proportion of missingness for indicators of latent classes and variables included in the imputation model; **Table S3.** Proportion of missingness for indicators of latent classes and variables included in the imputation model for region 1 (SAGE study - Africa: Ghana, South Africa); **Table S4.**

For peer review only - http://bmjopen.bmj.com/site/about/guidelines.xhtml

Proportion of missingness for indicators of latent classes and variables included in the imputation model for region 2 (SAGE study - Eastern Asia: China); **Table S5.** Proportion of missingness for indicators of latent classes and variables included in the imputation model for region 2 (SAGE study - Southern Asia: India); **Table S6.** Proportion of missingness for indicators of latent classes and variables included in the imputation model for region 2 (SAGE study - Eastern Europe: the Russian Federation); **Table S7.** Proportion of missingness for indicators of latent classes and variables included in the imputation model for region 2 (ELSA study- England); **Table S8.** Proportion of missingness for indicators of latent classes and variables included in the imputation model for region 2 (SHARE study - Northern Europe: Denmark, Sweden); **Table S9.** Proportion of missingness for indicators of latent classes and variables included in the imputation model for region 2 (SHARE study - Southern Europe: Greece, Italy, Spain); **Table S10.** Proportion of missingness for indicators of latent classes and variables included in the imputation model for region 2 (SHARE study - Western Europe: Austria, Belgium, France, Germany, Israel, Netherlands, Switzerland).

Additional file 2: **Table S1.** Association between latent multimorbidity membership and outcomes in individuals aged 50-64 and ≥65

Author contributions:

IB: Participated in the database management, drafted the paper, carried out the statistical analyses and worked on the interpretation of data. She also gave final approval of the version to be published and agreed to be accountable for all aspects of the work in ensuring that questions related to the accuracy or integrity of any part of the work are appropriately investigated and resolved; **AS:** Participated in the study design, database management, statistical support and critical revision of the paper. He also gave final approval of the version to be published and agreed to be accountable for all aspects of the work in ensuring that questions related to the accuracy or integrity of any part of the work are appropriately investigated and resolved; **LE:** Participated in the interpretation of data and critical revision of the paper; **HN:** She participated in critical revision of the paper and gave final approval of the version to be published and agreed to be accountable for all aspects of the work in ensuring that questions related to the accuracy or integrity of any part of the work are appropriately investigated and resolved; **MP:** Participated in the study design, and critical revision of the paper. He also gave final approval of the version to be published and agreed to be accountable for all aspects of the work in ensuring that questions related to the accuracy or integrity of any part of the work are appropriately investigated and resolved; **DF:** Participated in the study design, database management,

For peer review only - http://bmjopen.bmj.com/site/about/guidelines.xhtml

statistical support and critical revision of the paper. He also gave final approval of the version to be published and agreed to be accountable for all aspects of the work in ensuring that questions related to the accuracy or integrity of any part of the work are appropriately investigated and resolved; **JMH:** Participated in the study design, acquisition of data, interpretation of data and critical revision of the paper. He also gave final approval of the version to be published and agreed to be accountable for all aspects of the work in ensuring that questions related to the accuracy or integrity of any part of the work are appropriately investigated and resolved; **BO:** Participated in the acquisition of data, study design, database management, and critical revision of the paper. She also gave final approval of the version to be published and agreed to be accountable for all aspects of the work in ensuring that questions related to the accuracy or integrity of any part of the work are appropriately investigated and resolved.

References

1 WHO. Ageing and Health. https://www.who.int/news-room/fact-sheets/detail/ageing-and-health (11 December 2018, date last accessed)

2 Wang H, Naghavi M, Allen C, *et al.* Global, regional, and national life expectancy, all-cause mortality, and cause-specific mortality for 249 causes of death, 1980–2015: a systematic analysis for the Global Burden of Disease Study 2015. *Lancet* 2016;**388**:1459–544. doi:10.1016/S0140-6736(16)31012-1

3 Multimorbidity: a priority for global health research. 2018.

4 Parekh AK, Barton MB. The challenge of multiple comorbidity for the us health care system. *JAMA - J Am Med Assoc* 2010;**303**:1303–4. doi:10.1001/jama.2010.381

5 Mugisha J, Rosenbaum S, Ward PB, *et al.* Chronic physical conditions, multimorbidity and

For peer review only - http://bmjopen.bmj.com/site/about/guidelines.xhtml

physical activity across 46 low- and middle-income countries. *Int J Behav Nutr Phys Act* 2017;**14**. doi:10.1186/s12966-017-0463-5

6 Chatterji S, Byles J, Cutler D, *et al.* Health, functioning, and disability in older adults - Present status and future implications. *Lancet* 2015;**385**:563–75. doi:10.1016/S0140-6736(14)61462-8

7 Arokiasamy P, Uttamacharya U, Jain K, *et al.* The impact of multimorbidity on adult physical and mental health in low- and middle-income countries: What does the study on global ageing and adult health (SAGE) reveal? *BMC Med* 2015;**13**:1–16. doi:10.1186/s12916-015-0402-8

8 Bao J, Chua KC, Prina M, *et al.* Multimorbidity and care dependence in older adults: A longitudinal analysis of findings from the 10/66 study. *BMC Public Health* 2019;**19**:1–10. doi:10.1186/s12889-019-6961-4

9 Maximova T, Pati S, Biritwum RB, *et al.* The impact of multimorbidity on adult physical and mental health in low- and middle-income countries: what does the study on global ageing and adult health (SAGE) reveal? *BMC Med* 2015;**13**:1–16. doi:10.1186/s12916-015-0402-8

10 Afshar S, Roderick PJ, Kowal P, *et al. Applied demography and public health in the 21st century*. Cham, Switzerland: : Springer International Publishing AG 2016. doi:10.1249/mss.0000000000001652

11 Yen L, Valderas JM, McRae IS, *et al.* Multimorbidity and comorbidity of chronic diseases among the senior Australians: prevalence and patterns. *PLoS One* 2014;**9**:e83783. doi:10.1371/journal.pone.0083783

12 Baldo V, Corti MC, Boccuzzo G, *et al.* Multimorbidity patterns in high-need, high-cost elderly patients. *PLoS One* 2018;**13**:e0208875. doi:10.1371/journal.pone.0208875

13 Larsen FB, Pedersen MH, Friis K, *et al.* A Latent class analysis of multimorbidity and the

For peer review only

For peer review only - http://bmjopen.bmj.com/site/about/guidelines.xhtml

relationship to socio-demographic factors and health-related quality of life. A national population-based study of 162,283 Danish Adults. *PLoS One* 2017;**12**:1–17. doi:10.1371/journal.pone.0169426

14 Alcalá-Nalvaiz JT, Sicras-Mainar A, Calderón-Larrañaga A, *et al.* Multimorbidity Patterns in Primary Care: Interactions among Chronic Diseases Using Factor Analysis. *PLoS One* 2012;**7**:e32190. doi:10.1371/journal.pone.0032190

15 Guisado-Clavero M, Roso-Llorach A, López-Jimenez T, *et al.* Multimorbidity patterns in the elderly: A prospective cohort study with cluster analysis. *BMC Geriatr* 2018;**18**:1–11. doi:10.1186/s12877-018-0705-7

16 Sanchez-Niubo A, Egea-Cortés L, Olaya B et al. Cohort profile: the ageing trajectories of health - longitudinal ppportunities and synergies (ATHLOS) project. *Int J Epidemiol* 2019;**0**:1–11. doi:org/10.1093/ije/dyz077

17 Kowal P, Chatterji S, Naidoo N, *et al.* Data resource profile: The World Health Organization study on global AGEing and adult health (SAGE). *Int J Epidemiol* 2013;**41**:1639–49. doi:10.1093/ije/dys210

18 Steptoe A, Breeze E, Banks J, *et al.* Cohort profile: The English Longitudinal Study of Ageing. *Int J Epidemiol* 2013;**42**:1640–8. doi:10.1093/ije/dys168

19 Börsch-Supan A, Brandt M, Hunkler C, *et al.* Data resource profile: The survey of health, ageing and retirement in europe (SHARE). *Int J Epidemiol* 2013;**42**:992–1001. doi:10.1093/ije/dyt088

20 IDA TWBI. World Bank Open Data. https://data.worldbank.org/ (5 March 2019, date last accessed)

21 Fortier I, Raina P, Van den Heuvel ER, Griffith LE, Craig C, Saliba M, Doiron D, Stolk RP,

For peer review only

For peer review only - http://bmjopen.bmj.com/site/about/guidelines.xhtml

Knoppers BM, Ferretti V, Granda P BP. Maelstrom Research guidelines for rigorous retrospective data harmonization. 2017;:103–5. doi:10.1093/ije/dyw075

22 Ronald C. Kessler TBÜ. The World Mental Health (WMH) survey initiative version of the World Health Organization (WHO) Composite Diagnostic Interview (CIDI). *Int J Methods Psychiatr Res* 2004;**13**:93–121. doi:org/10.1002/mpr.168

23 Radloff L S. The CES-D scale: A self report depression scale for research in the general population. *Appl Psychol Meas* 1977;**1**:385–401. doi:10.1002/ardp.19552881111

24 Reischies F, Lobo A, Turrina C, *et al.* Development of the EURO–D scale – a European Union initiative to compare symptoms of depression in 14 European centres. *Br J Psychiatry* 2008;**174**:330–8. doi:10.1192/bjp.174.4.330

25 Zivin K, Llewellyn DJ, Lang IA, *et al.* Depression among older adults in the United States and England. *Am J Geriatr Psychiatry* 2010;**18**:1036–44. doi:10.1097/JGP.0b013e3181dba6d2

26 Prince MJ, Beekman ATF, Deeg DJH, *et al.* Depression symptoms in late life assessed during the EURO-D scale. Effect of age, gender and marital status in 14 European centres. *Br J Psychiatry* 1999;**174**:339–45. doi:10.1192/bjp.174.4.339

27 Morris JC, Heyman A, Mohs RC, Hughes JP, van Belle G, Fillenbaum G et al. The consortium to establish a registry for Alzheimer's disease (CERAD). Part I. Clinical and neuropsychological assesment of Alzheimer's disease. *Neurology* 1989;**39**:1159–65. doi:10.1212/wnl.39.9.1159

28 WHO. Country groupings. https://www.who.int/quantifying_ehimpacts/global/ebdcountgroup/en/ (7 February 2019, date last accessed)

29 United Nations Statistical Division (UNSD). Countries or areas/geographical regions. https://unstats.un.org/unsd/methodology/m49/ (7 February 2019, date last accessed)

For peer review only - http://bmjopen.bmj.com/site/about/guidelines.xhtml

30 Lanza ST, Dziak JJ, Huang L WA, Collins L. *LCA Stata plugin users ' guide (version 1.2.1)*. Penn State: : University Park: The Methodology Center 2018.

31 Tein JY, Coxe S, Cham H. Statistical power to detect the correct number of classes in latent profile analysis. *Struct Equ Model* 2013;**20**:640–57. doi:10.1080/10705511.2013.824781

32 Nylund-Gibson K, Choi AY. Ten frequently asked questions about latent class analysis. *Transl Issues Psychol Sci* 2018;**4**:440–61. doi:10.1037/tps0000176

33 Masyn KE. Latent Class Analysis and Finite Mixture Modeling. Published Online First: 21 March 2013. doi:10.1093/OXFORDHB/9780199934898.013.0025

34 StataCorp. *Stata: Release 13. Statistical Software*. College St. 2013.

35 Rubin DB. *Multiple Imputation for nonresponse in surveys*. United States of America: : John Wiley & Sons 1987. doi:10.2307/3172772

36 Garin N, Olaya B, Perales J, *et al.* Multimorbidity patterns in a national representative sample of the Spanish adult population. *PLoS One* 2014;**9**:e84794. doi:10.1371/journal.pone.0084794

37 Olaya B, Moneta MV, Caballero FF, *et al.* Latent class analysis of multimorbidity patterns and associated outcomes in Spanish older adults: A prospective cohort study. *BMC Geriatr* 2017;**17**. doi:10.1186/s12877-017-0586-1

38 Whitson HE, Johnson KS, Sloane R, *et al.* Identifing patterns of multimorbidity in older americans: application of latent class analysis. *J Am Geriatr Soc* 2016;**64**:1668–73. doi:10.1111/jgs.14201.Identifying

39 Prados-Torres A, Calderón-Larrañaga A, Hancco-Saavedra J, *et al.* Multimorbidity patterns: A systematic review. *J Clin Epidemiol* 2014;**67**:254–66. doi:10.1016/j.jclinepi.2013.09.021

40 Alberti KGM, Zimmet P, Shaw J. Metabolic syndrome -a new world-wide definition. A

For peer review only

For peer review only - http://bmjopen.bmj.com/site/about/guidelines.xhtml

consensus statement from the International Diabetes Federation. *Abstr Appl Anal* 2006;**23**:469–80. doi:10.1155/2010/307409

41 Margaretten M, Julian L, Katz P, *et al.* Depression in patients with rheumatoid arthritis: description, causes and mechanisms. *Int J Clin Rheumtol* 2011;**6**:617–23. doi:10.2217/IJR.11.6.Depression

42 Druss BG, Walker ER. Mental disorders and medical comorbidity. 2011. https://www.rwjf.org/content/dam/farm/reports/issue_briefs/2011/rwjf69438/subassets/rwjf69438_1

43 Barbara Messner DB. Smoking and cardiovascular disease: Mechanisms of endothelial dysfunction and early atherogenesis. 2014;**34**:509–15. doi:10.1161/ATVBAHA.113.300156

44 Meade T, Manolios N, Cumming SR, *et al.* Cognitive impairment in rheumatoid arthritis: A systematic review. *Arthritis Care Res* 2018;**70**:39–52. doi:10.1002/acr.23243

45 Lam RW, Kennedy SH, McIntyre RS, *et al.* Cognitive dysfunction in major depressive disorder: effects on psychosocial functioning and implications for treatment. *Can J Psychiatry* 2014;**59**:649–54. doi:10.1177/070674371405901206

46 Dodd JW. Lung disease as a determinant of cognitive decline and dementia. *Alzheimer's Res Ther* 2015;**7**:1–8. doi:10.1186/s13195-015-0116-3

47 Rose Mayeda E, Whitmer RA, Yaffe K. Diabetes and cognition. *Clin Geriatr Med* 2015;**31**:101–ix. doi:10.1016/j.cger.2014.08.021

48 Walker KA, Power MC, Gottesman RF. Defining the relationship between hypertension, cognitive decline, and dementia: a review. 2018;**19**:24. doi:10.1007/s11906-017-0724-3

49 Deckers K, Schievink SHJ, Rodriquez MMF, *et al.* Coronary heart disease and risk for cognitive impairment or dementia: Systematic review and meta-analysis. *PLoS One* 2017;**12**:1–15.

For peer review only - http://bmjopen.bmj.com/site/about/guidelines.xhtml

doi:10.1371/journal.pone.0184244

50 Leon DA, Shkolnikov VM, McKee M, *et al.* Alcohol increases circulatory disease mortality in Russia: Acute and chronic effects or misattribution of cause? *Int J Epidemiol* 2010;**39**:1279–90. doi:10.1093/ije/dyq102

51 Petrukhin IS, Lunina EY. Cardiovascular disease risk factors and mortality in Russia: Challenges and barriers. *Public Health Rev* 2012;**33**:436–49. doi:10.1007/BF03391645

52 Y. He, M. Zhang, E. H. B. Lin et al. Mental disorders among persons with arthritis: results from the World Mental Health Surveys. 2008;**38**:1639–50. doi:10.1017/S0033291707002474

53 Jamrozik E, Musk AW. Respiratory health issues in the Asia-Pacific region: An overview. *Respirology* 2011;**16**:3–12. doi:10.1111/j.1440-1843.2010.01844.x

54 Sandeep Grover, Alakananda Dutt AA. An overview of Indian research in depression. *Indian J Psychiatry* 2010;**52**:178–88. doi:10.4103/0019-5545.69231

55 Gu L, Xie J, Long J, *et al.* Epidemiology of major depressive disorder in Mainland China: A Systematic review. *PLoS One* 2013;**8**:e65356. doi:10.1371/journal.pone.0065356

56 Hou Z, Jiang W, Yin Y, *et al.* The current situation on major depressive disorder in China: Research on mechanisms and clinical practice. *Neurosci Bull* 2016;**32**:389–97. doi:10.1007/s12264-016-0037-6

57 Castro-Costa E, Dewey M, Stewart R, *et al.* Ascertaining late-life depressive symptoms in Europe: An evaluation of the survey version of the EURO-D scale in 10 nations. The SHARE project. *Int J Methods Psychiatr Res* 2008;**17**:12–29. doi:10.1002/mpr.236

58 Martin LM, Leff M, Calonge N, *et al.* Validation of self-reported chronic conditions and health services in a managed care population. *Am J Prev Med* 2000;**18**:215–8. doi:10.1016/S0749-3797(99)00158-0

For peer review only - http://bmjopen.bmj.com/site/about/guidelines.xhtml

59 Finch H. A comparison of statistics for assessing model invariance in latent class analysis. *Open J Stat* 2015;**5**:191–210. doi:10.4236/ojs.2015.53022

60 White IR, Carlin JB. Bias and efficiency of multiple imputation compared with complete-case analysis for missing covariate values. *Stat Med* 2010;**29**:2920–31. doi:10.1002/sim.3944

61 Sterne JAC, White IR, Carlin JB, *et al.* Multiple imputation for missing data in epidemiological and clinical research: Potential and pitfalls. *BMJ* 2009;**339**:157–60. doi:10.1136/bmj.b2393

Figure 1 Prevalence of diseases in the three latent classes in the total sample and by regions (subsample 50-64 years)

Figure 2 Prevalence of diseases in the three latent classes in the total sample and by regions (subsample +65 years)

For peer review only

For peer review only - http://bmjopen.bmj.com/site/about/guidelines.xhtml

Multimorbidity latent classes

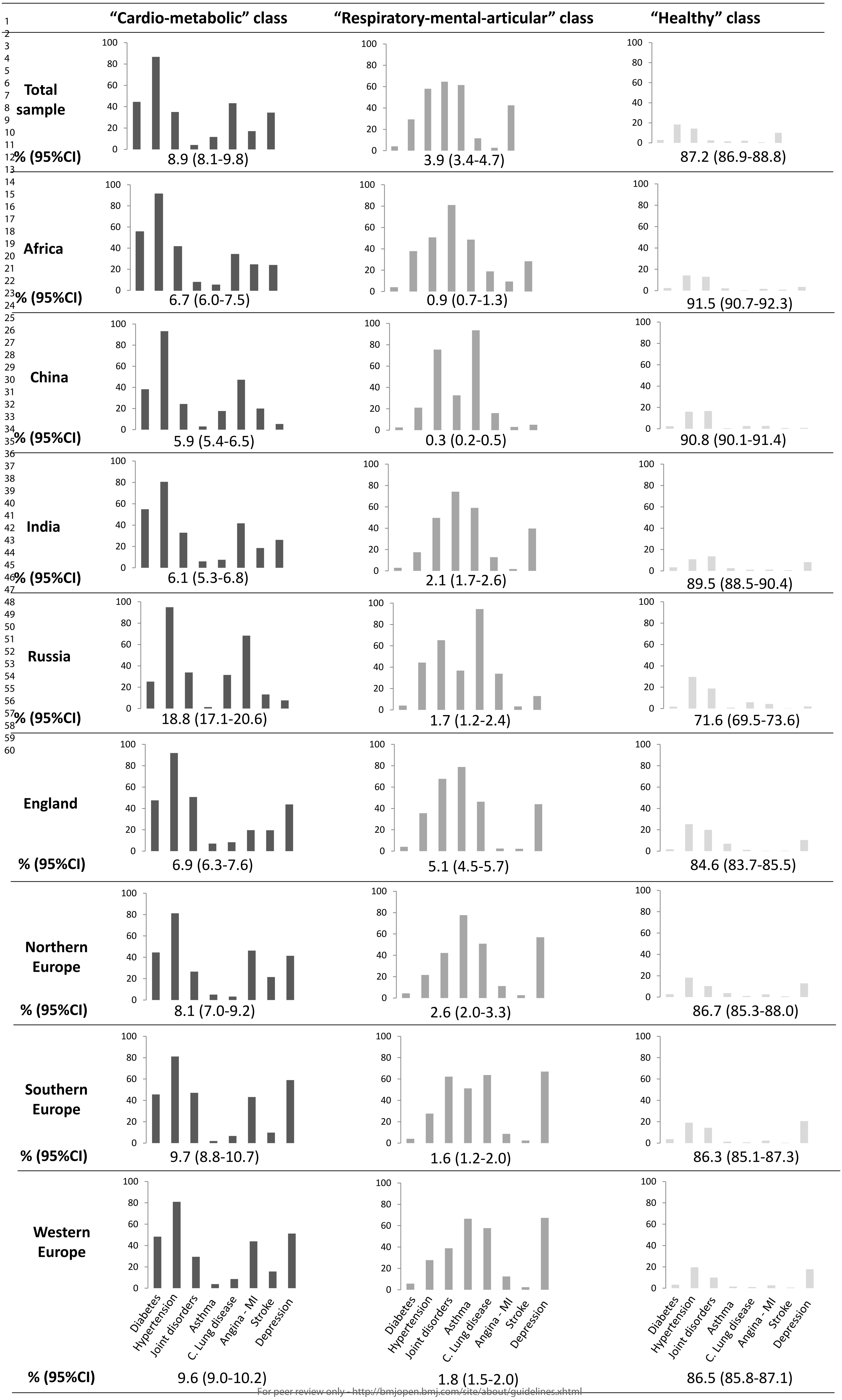

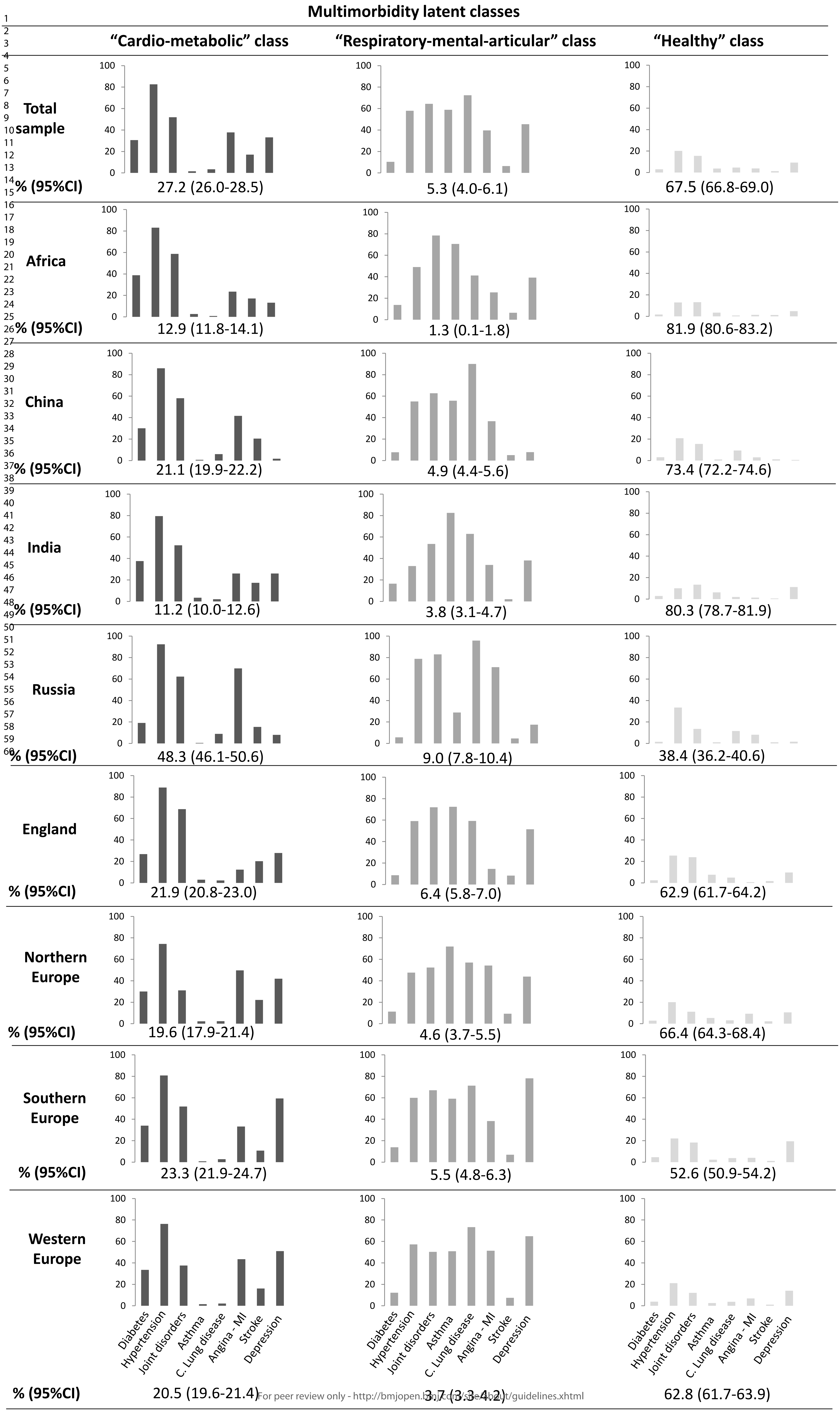

For peer review only - http://bmjopen.bmj.com/site/about/guidelines.xhtml

Table S1. Proportion of missingness in the variables of interest in Mexico

	n (%) missingness
**(a) Indicators of multimorbidity classes**	
Diabetes	2814 (51.65)
Hypertension	2815 (51.67)
Joint disorders	2814 (51.65)
Asthma	2814 (51.65)
Chronic lung disease	2814 (51.65)
Myocardial infarction – Angina	2814 (51.65)
Stroke	2814 (51.65)
Depression	2815 (51.67)
**(b) Variables used in the regression model**	
Sex	2706 (49.67)
Age	2707 (49.69)
Country	0 (0.00)
Study	0 (0.00)
Marital status	2811 (51.60)
Education	3264 (59.91)
Wealth	1922 (35.28)
Loneliness	2819 (51.74)
Ever smoked	2814 (51.65)
Vigorous exercise in last 2 weeks	2814 (51.65)
Self-rated health	2811 (51.6)
ADL – Using the toilet	2814 (51.65)
ADL – Bathing or showering	2815 (51.67)
ADL – Getting dressed	0 (0.00)
ADL - Eating	2814 (51.65)
ADL – Getting in or out of bed	2813 (51.63)
ADL – Moving around the house	2817 (51.71)
Memory: Immediate recall	2854 (52.39)
Memory: Delayed recall	2854 (52.39)
Verbal fluency	2852 (52.35)

For peer review only

Table S2. Overall proportion of missingness for indicators of latent classes and variables included in the imputation model

(a) Indicators of multimorbidity classes	
	n (%) missingness
Diabetes	62 (0.08)
Hypertension	154 (0.21)
Joint disorders	31 (0.04)
Asthma	82 (0.11)
Chronic lung disease	51 (0.07)
Myocardial infarction – Angina	63 (0.08)
Stroke	41 (0.05)
Depression	1233 (1.70)
(b) Variables used in the regression model	
Sex	0 (0.00)
Age	0 (0.00)
Country	0 (0.00)
Study	0 (0.00)
Marital status	106 (0.14)
Education	11475 (15.91)
Wealth	844 (1.17)
Loneliness	10985 (15.23)
Ever smoked	201 (0.27)
Vigorous exercise in last 2 weeks	258 (0.35)
Self-rated health	218 (0.30)
ADL – Using the toilet	355 (0.49)
ADL – Bathing or showering	340 (0.47)
ADL – Getting dressed	304 (0.42)
ADL - Eating	400 (0.55)
ADL – Getting in or out of bed	310 (0.42)
ADL – Moving around the house	345 (0.47)
Memory: Immediate recall	1442 (1.99)
Memory: Delayed recall	1448 (2.00)
Verbal fluency	1641 (2.27)

For peer review only

Table S3. Proportion of missingness for indicators of latent classes and variables included in the imputation model for region 1 (SAGE study - Africa: Ghana, South Africa)

(a) Indicators of multimorbidity classes	
	n (%) missingness
Diabetes	1 (0.01)
Hypertension	1 (0.01)
Joint disorders	3 (0.03)
Asthma	1 (0.01)
Chronic lung disease	1 (0.01)
Myocardial infarction – Angina	3 (0.03)
Stroke	1 (0.01)
Depression	17 (0.21)
(b) Variables used in the regression model	
Sex	0 (0.00)
Age	0 (0.00)
Country	0 (0.00)
Study	0 (0.00)
Marital status	89 (1.11)
Education	3705 (46.6)
Wealth	23 (0.28)
Loneliness	48 (0.60)
Ever smoked	7 (0.08)
Vigorous exercise in last 2 weeks	12 (0.15)
Self-rated health	15 (0.18)
ADL – Using the toilet	40 (0.50)
ADL – Bathing or showering	32 (0.40)
ADL – Getting dressed	27 (0.33)
ADL - Eating	31 (0.38)
ADL – Getting in or out of bed	32 (0.40)
ADL – Moving around the house	35 (0.44)
Memory: Immediate recall	36 (0.45)
Memory: Delayed recall	31 (0.38)
Verbal fluency	9 (0.11)

For peer review only

Table S4. Proportion of missingness for indicators of latent classes and variables included in the imputation model for region 2 (SAGE study - Eastern Asia: China)

(a) Indicators of multimorbidity classes	
	n (%) missingness
Diabetes	35 (0.27)
Hypertension	127 (0.98)
Joint disorders	2 (0.01)
Asthma	53 (0.41)
Chronic lung disease	23 (0.17)
Myocardial infarction – Angina	34 (0.26)
Stroke	13 (0.10)
Depression	146 (1.13)
(b) Variables used in the regression model	
Sex	0 (0.00)
Age	0 (0.00)
Country	0 (0.00)
Study	0 (0.00)
Marital status	8 (0.06)
Education	3105 (24.18)
Wealth	18 (0.14)
Loneliness	74 (0.57)
Ever smoked	2 (0.01)
Vigorous exercise in last 2 weeks	45 (0.35)
Self-rated health	16 (0.12)
ADL – Using the toilet	51 (0.39)
ADL – Bathing or showering	52 (0.40)
ADL – Getting dressed	41 (0.31)
ADL - Eating	46 (0.35)
ADL – Getting in or out of bed	36 (0.28)
ADL – Moving around the house	48 (0.37)
Memory: Immediate recall	317 (2.46)
Memory: Delayed recall	399 (3.10)
Verbal fluency	335 (2.60)

For peer review only

Table S5. Proportion of missingness for indicators of latent classes and variables included in the imputation model for region 2 (SAGE study - Southern Asia: India)

(a) Indicators of multimorbidity classes	
	n (%) missingness
Diabetes	0 (0.00)
Hypertension	1 (0.01)
Joint disorders	1 (0.01)
Asthma	0 (0.00)
Chronic lung disease	0 (0.00)
Myocardial infarction – Angina	0 (0.00)
Stroke	1 (0.01)
Depression	1 (0.01)
(b) Variables used in the regression model	
Sex	0 (0.00)
Age	0 (0.00)
Country	0 (0.00)
Study	0 (0.00)
Marital status	0 (0.00)
Education	0 (0.00)
Wealth	38 (0.57)
Loneliness	10 (0.15)
Ever smoked	1 (0.01)
Vigorous exercise in last 2 weeks	0 (0.00)
Self-rated health	0 (0.00)
ADL – Using the toilet	19 (0.28)
ADL – Bathing or showering	12 (0.18)
ADL – Getting dressed	11 (0.16)
ADL - Eating	73 (1.11)
ADL – Getting in or out of bed	7 (0.10)
ADL – Moving around the house	24 (0.36)
Memory: Immediate recall	88 (1.34)
Memory: Delayed recall	88 (1.34)
Verbal fluency	71 (1.08)

For peer review only

Table S6. Proportion of missingness for indicators of latent classes and variables included in the imputation model for region 2 (SAGE study - Eastern Europe: the Russian Federation)

(a) Indicators of multimorbidity classes	
	n (%) missingness
Diabetes	5 (0.12)
Hypertension	4 (0.10)
Joint disorders	3 (0.07)
Asthma	6 (0.15)
Chronic lung disease	5 (0.12)
Myocardial infarction – Angina	5 (0.12)
Stroke	5 (0.12)
Depression	14 (0.36)
**(b) Variables used in the regression model**	
Sex	0 (0.00)
Age	0 (0.00)
Country	0 (0.00)
Study	0 (0.00)
Marital status	6 (0.15)
Education	41 (1.05)
Wealth	5 (0.12)
Loneliness	79 (2.03)
Ever smoked	1 (0.02)
Vigorous exercise in last 2 weeks	0 (0.00)
Self-rated health	5 (0.12)
ADL – Using the toilet	47 (1.20)
ADL – Bathing or showering	46 (1.18)
ADL – Getting dressed	27 (0.69)
ADL - Eating	52 (1.33)
ADL – Getting in or out of bed	37 (0.95)
ADL – Moving around the house	40 (1.02)
Memory: Immediate recall	153 (3.93)
Memory: Delayed recall	81 (2.08)
Verbal fluency	208 (5.35)

For peer review only

Table S7. Proportion of missingness for indicators of latent classes and variables included in the imputation model for region 2 (ELSA study- England)

	n (%) missingness
**(a) Indicators of multimorbidity classes**	
Diabetes	2 (0.01)
Hypertension	2 (0.01)
Joint disorders	3 (0.02)
Asthma	3 (0.02)
Chronic lung disease	3 (0.02)
Myocardial infarction – Angina	2 (0.01)
Stroke	2 (0.01)
Depression	372 (3.23)
**(b) Variables used in the regression model**	
Sex	0 (0.00)
Age	0 (0.00)
Country	0 (0.00)
Study	0 (0.00)
Marital status	0 (0.00)
Education	1023 (8.88)
Wealth	330 (2.86)
Loneliness	356 (3.09)
Ever smoked	173 (1.50)
Vigorous exercise in last 2 weeks	176 (1.52)
Self-rated health	175 (1.51)
ADL – Using the toilet	173 (1.50)
ADL – Bathing or showering	173 (1.50)
ADL – Getting dressed	173 (1.50)
ADL - Eating	173 (1.50)
ADL – Getting in or out of bed	173 (1.50)
ADL – Moving around the house	173 (1.50)
Memory: Immediate recall	360 (3.12)
Memory: Delayed recall	373 (3.23)
Verbal fluency	362 (3.14)

For peer review only

Table S8. Proportion of missingness for indicators of latent classes and variables included in the imputation model for region 2 (SHARE study - Northern Europe: Denmark, Sweden)

(a) Indicators of multimorbidity classes	
	n (%) missingness
Diabetes	2 (0.04)
Hypertension	2 (0.04)
Joint disorders	2 (0.04)
Asthma	2 (0.04)
Chronic lung disease	2 (0.04)
Myocardial infarction – Angina	2 (0.04)
Stroke	2 (0.04)
Depression	78 (1.70)
(b) Variables used in the regression model	
Sex	0 (0.00)
Age	0 (0.00)
Country	0 (0.00)
Study	0 (0.00)
Marital status	0 (0.00)
Education	52 (1.13)
Wealth	6 (0.13)
Loneliness	1384 (30.26)
Ever smoked	4 (0.08)
Vigorous exercise in last 2 weeks	6 (0.13)
Self-rated health	2 (0.04)
ADL – Using the toilet	3 (0.06)
ADL – Bathing or showering	3 (0.06)
ADL – Getting dressed	3 (0.06)
ADL - Eating	3 (0.06)
ADL – Getting in or out of bed	3 (0.06)
ADL – Moving around the house	3 (0.06)
Memory: Immediate recall	68 (1.48)
Memory: Delayed recall	66 (1.44)
Verbal fluency	83 (1.81)

For peer review only

Table S9. Proportion of missingness for indicators of latent classes and variables included in the imputation model for region 2 (SHARE study - Southern Europe: Greece, Italy, Spain)

(a) Indicators of multimorbidity classes	
	n (%) missingness
Diabetes	12 (0.16)
Hypertension	12 (0.16)
Joint disorders	12 (0.16)
Asthma	12 (0.16)
Chronic lung disease	12 (0.16)
Myocardial infarction – Angina	12 (0.16)
Stroke	12 (0.16)
Depression	194 (2.59)
(b) Variables used in the regression model	
Sex	0 (0.00)
Age	0 (0.00)
Country	0 (0.00)
Study	0 (0.00)
Marital status	1 (0.01)
Education	20 (0.26)
Wealth	95 (1.27)
Loneliness	2715 (36.37)
Ever smoked	3 (0.04)
Vigorous exercise in last 2 weeks	4 (0.05)
Self-rated health	0 (0.00)
ADL – Using the toilet	3 (0.04)
ADL – Bathing or showering	3 (0.04)
ADL – Getting dressed	3 (0.04)
ADL - Eating	3 (0.04)
ADL – Getting in or out of bed	3 (0.04)
ADL – Moving around the house	3 (0.04)
Memory: Immediate recall	125 (1.67)
Memory: Delayed recall	125 (1.67)
Verbal fluency	176 (2.35)

For peer review only

Table S10. Proportion of missingness for indicators of latent classes and variables included in the imputation model for region 2 (SHARE study - Western Europe: Austria, Belgium, France, Germany, Israel, Netherlands, Switzerland)

(a) Indicators of multimorbidity classes	
	n (%) missingness
Diabetes	5 (0.02)
Hypertension	5 (0.02)
Joint disorders	5 (0.02)
Asthma	5 (0.02)
Chronic lung disease	5 (0.02)
Myocardial infarction – Angina	5 (0.02)
Stroke	5 (0.02)
Depression	411 (2.36)
(b) Variables used in the regression model	
Sex	0 (0.00)
Age	0 (0.00)
Country	0 (0.00)
Study	0 (0.00)
Marital status	2 (0.01)
Education	165 (0.95)
Wealth	329 (1.89)
Loneliness	6319 (36.42)
Ever smoked	10 (0.05)
Vigorous exercise in last 2 weeks	15 (0.08)
Self-rated health	5 (0.02)
ADL – Using the toilet	19 (0.10)
ADL – Bathing or showering	19 (0.10)
ADL – Getting dressed	19 (0.10)
ADL - Eating	19 (0.10)
ADL – Getting in or out of bed	19 (0.10)
ADL – Moving around the house	19 (0.10)
Memory: Immediate recall	295 (1.7)
Memory: Delayed recall	285 (1.64)
Verbal fluency	397 (2.28)

For peer review only

Table S1. Association between latent multimorbidity membership and outcomes in individuals aged 50-64 and ≥65

Outcomes[a]	Aged 50-64		Aged ≥65	
	"Cardio-metabolic" class	"Respiratory-mental-articular" class	"Cardio-metabolic" class	"Respiratory-mental-articular" class
Sex				
Woman	1.0	1.0	1.0	1.0
Male	0.9 (0.8-1.0)	0.6 (0.5-0.7)	0.7 (0.7-0.8)	0.9 (0.8-1.0)
Age (years)	1.1 (1.1-1.1)	1.1 (1.0-1.1)	1.0 (1.0-1.0)	1.0 (1.0-1.0)
Marital status				
Single	1.0	1.0	1.0	1.0
Married	0.9 (0.8-1.1)	0.7 (0.5-1.0)	1.2 (1.00-1.4)	1.2 (0.9-1.6)
Divorced	1.1 (0.9-1.4)	1.7 (1.2-2.4)	1.1 (1.0-1.4)	1.8 (1.2-2.6)
Widowed	1.4 (1.1-1.7)	1.1 (0.8-1.6)	1.5 (1.3-1.8)	1.6 (1.2-2.3)
Education level				
Primary or less	1.0	1.0	1.0	1.0
Secondary	0.9 (0.9-1.0)	0.7 (0.6-0.8)	1.0 (0.9-1.0)	0.7 (0.6-0.8)
Tertiary	0.8 (0.7-0.9)	0.6 (0.5-0.7)	0.9 (0.8-1.0)	0.6 (0.5-0.7)
Wealth				
1st (worse)	1.0	1.0	1.0	1.0
2nd	1.0 (0.9-1.1)	0.8 (0.6-1.0)	1.1 (1.0-1.2)	1.1 (0.9-1.2)
3rd	0.9 (0.9-1.1)	0.7 (0.5-0.8)	1.1 (1.0-1.2)	1.0 (0.9-1.2)
4th	0.9 (0.8-1.0)	0.6 (0.5-0.8)	1.1 (1.0-1.2)	0.8 (0.7-1.0)
5th (best)	0.8 (0.7-0.9)	0.5 (0.4-0.6)	1.2 (1.0-1.2)	0.7 (0.6-0.9)
Region				
Africa	1.0	1.0	1.0	1.0
China	0.9 (0.8-1.0)	0.3 (0.2-0.5)	1.8 (1.6-2.1)	4.1 (3.0-5.7)
India	0.9 (0.8-1.1)	2.2 (1.5-3.2)	0.9 (0.8-1.0)	2.9 (2.0-4.2)
Russia	3.6 (3.0-4.2)	2.2 (1.4-3.5)	8.0 (7.0-9.2)	14.5 (10.3-20.3)
England	1.1 (0.9-1.3)	5.6 (4.1-7.7)	2.2 (2.0-2.5)	6.2 (4.5-8.5)
Northern Europe	1.3 (1.1-1.5)	2.8 (1.9-4.1)	1.9 (1.6-2.2)	4.2 (2.9-6.1)
Southern Europe	1.5 (1.3-1.8)	1.7 (1.2-2.5)	2.8 (2.5-3.2)	6.5 (4.7-9.0)
Western Europe	1.5 (1.3-1.7)	1.9 (1.3-2.6)	2.1 (1.9-2.3)	3.6 (2.6-5.0)
Loneliness (yes/no)	1.9 (1.8-2.1)	2.5 (2.1-3.0)	1.4 (1.4-1.6)	1.9 (1.7-2.2)
Ever smoked (yes/no)	1.0 (0.9-1.1)	1.7 (1.5-2.0)	0.8 (0.8-0.8)	1.4 (1.3-1.6)
Physical activity (yes/no)	0.5 (0.5-0.5)	0.6 (0.5-0.7)	0.5 (0.5-0.6)	0.5 (0.4-0.6)
Limitations in ADL (yes/no)	2.5 (2.3-2.7)	3.2 (2.7-3.7)	1.8 (1.7-1.9)	2.8 (2.5-3.1)
Self-rated health				
Good	1.0	1.0	1.0	1.0
Moderate	3.7 (3.3-3.9)	2.5 (2.1-2.9)	2.5 (2.3-2.7)	3.9 (3.3-4.6)
Poor	8.5 (7.7-9.4)	7.4 (6.1-8.8)	4.3 (4.6)	10.9 (9.3-12.8)
Memory: Immediate recall	0.9 (0.9-0.9)	0.9 (0.9-1.0)	0.9 (0.9-0.9)	0.9 (0.9-0.9)
Memory: Delayed recall	0.9 (0.9-0.9)	0.9 (0.8-0.9)	0.9 (0.9-0.9)	0.9 (0.9-0.9)
Verbal fluency	1.0 (1.0-1.0)	1.0 (1.0-1.0)	1.0 (0.9-1.00)	1.0 (0.9-1.0)

Note: The reference group for the multimorbidity group variable was the "Healthy" class
Relative Risk Ratios (95% confidence interval) from multinomial logistic regression models
Models were run in 100 imputed datasets and results combined using Rubin's rules
ADL Activities of Daily Living
[a]Unadjusted analyses.

For peer review only

STROBE CHECKLIST: "MULTIMORBIDITY PATTERNS IN LOW-MIDDLE AND HIGH INCOME REGIONS: A MULTI-REGION LATENT CLASS ANALYSIS USING ATHLOS HARMONIZED COHORTS"

1. Title and abstract

(a) Indicate the study's design with a commonly used term in the title or the abstract **(page 1 and 3)**

(b) Provide in the abstract an informative and balanced summary of what was done and what was found **(page 3)**

Introduction

2. Background/rationale: Explain the scientific background and rationale for the investigation being reported **(page 5-6)**

3. Objectives: State specific objectives, including any prespecified hypotheses **(page 6, lines 10-13)**

Methods

4. Study design: Present key elements of study design early in the paper **(page 6, lines 16-19)**

5. Setting: Describe the setting, locations, and relevant dates, including periods of recruitment, exposure, follow-up, and data collection **(page 6, lines 20-24, page 7, lines 1-3).**

6. Participants: Give the eligibility criteria, and the sources and methods of selection of Participants **(page 7, lines 4-9)**.

7. Variables: Clearly define all outcomes, exposures, predictors, potential confounders, and effect modifiers. Give diagnostic criteria, if applicable. **(page 7, lines 13- page 8)**.

8. Data sources/measurement: For each variable of interest, give sources of data and details of methods of assessment (measurement). Describe comparability of assessment methods if there is more than one group **(page 7, lines 13- page 8).**

9. Bias: Describe any efforts to address potential sources of bias **(page 9, lines 19-23).**

10. Study size: Explain how the study size was arrived at **(page 7, lines 4-9)**.

11. Quantitative variables: Explain how quantitative variables were handled in the analyses. If applicable, describe which groupings were chosen and why. **(page 7, lines 13- page 8)**.

12. Statistical methods **(page 9-10)**:

(a) Describe all statistical methods, including those used to control for confounding

(b) Describe any methods used to examine subgroups and interactions

(c) Explain how missing data were addressed

(d) If applicable, describe analytical methods taking account of sampling strategy

(e) Describe any sensitivity analyses

Results

13. Participants **(page 7, lines 4-9)**:

For peer review only

(a) Report numbers of individuals at each stage of study—eg numbers potentially eligible, examined for eligibility, confirmed eligible, included in the study, completing follow-up, and analysed
(b) Give reasons for non-participation at each stage
(c) Consider use of a flow diagram

14. Descriptive data **(page 10, lines 8-18; page 11, lines 1-7)**:

(a) Give characteristics of study participants (eg demographic, clinical, social) and information on exposures and potential confounders
(b) Indicate number of participants with missing data for each variable of interest **(Suplemmentary file 1)**

15. Outcome data: Report numbers of outcome events or summary measures **(pages 12-15)**.
16. Main results **(page 15, lines 14-21; to page 17)**:

(a) Give unadjusted estimates and, if applicable, confounder-adjusted estimates and their precision (eg, 95% confidence interval). Make clear which confounders were adjusted for and why they were included
(b) Report category boundaries when continuous variables were categorized
(c) If relevant, consider translating estimates of relative risk into absolute risk for a meaningful time period

17. Other analyses: Report other analyses done—eg analyses of subgroups and interactions, and sensitivity analyses. **Not applicable.**

Discussion

18. Key results: Summarise key results with reference to study objectives **(page 17, lines 5-8)**.
19. Limitations: Discuss limitations of the study, taking into account sources of potential bias or imprecision. Discuss both direction and magnitude of any potential bias **(page 21, lines 19-24, page 22, 1-14).**
20. Interpretation: Give a cautious overall interpretation of results considering objectives, limitations, multiplicity of analyses, results from similar studies, and other relevant evidence **(pages 18-21)**.
21. Generalisability: Discuss the generalisability (external validity) of the study results **(page 19, 13-15)**.

Other information

22. Funding: Give the source of funding and the role of the funders for the present study and, if applicable, for the original study on which the present article is based **(page 23, lines 20-22, page 24, lines 1-6)**.

For peer review only
